# Extendable stapling of unprotected peptides by crosslinking two amines with *o*-phthalaldehyde

**DOI:** 10.1038/s41467-022-27985-7

**Published:** 2022-01-14

**Authors:** Bo Li, Lan Wang, Xiangxiang Chen, Xin Chu, Hong Tang, Jie Zhang, Gang He, Li Li, Gong Chen

**Affiliations:** 1 State Key Laboratory and Institute of Elemento-Organic Chemistry, College of Chemistry, Nankai University, Tianjin, 300071 China; 2 Beijing Key Laboratory of Active Substances Discovery and Druggability Evaluation, Institute of Materia Medica, Chinese Academy of Medical Sciences, Peking Union Medical College, Beijing, 100050 China

**Keywords:** Peptides, Synthetic chemistry methodology

## Abstract

Peptide modification methods that do not rely on the cysteine residue are underdeveloped, and their development could greatly expand the current toolbox for peptide chemistry. During the course of preliminary investigations into the classical *ortho*-phthalaldehyde (OPA)-amine-thiol condensation reaction, we found that in the absence of thiol, OPA readily condenses with two primary alkyl amines to form a class of underexplored isoindolin-1-imine compounds under mild aqueous conditions. From the intramolecular version of this OPA-2amines reaction, an efficient and selective methodology using mild reaction conditions has been developed for stapling unprotected peptides via crosslinking of two amino groups in both an end-to-side and side-to-side fashion. The stapling method is superfast and broadly applicable for various peptide substrates with the reacting amino groups separated by a wide range of different amino acid units. The macrocyclization reactions of selected substrates are completed within 10 seconds at 5 mM concentration and within 2 minutes at 50 μM concentration. Importantly, the resulting cyclized peptides with an isoindolinimine linkage can be extended in a one-pot sequential addition manner with several different electron-deficient π electrophiles, thereby generating more complex structures.

## Abstract (web summary)

Methods for peptide stapling, or covalently linking amino acid residues to create a non-linear structure, mostly rely on cysteine residues, which imposes a significant practical limitation. Here the authors disclose a method to chemoselectively macrocyclize two free-amine-containing residues in mild, peptide-relevant conditions, using a commercially available reagent.

## Introduction

**Fig. 1:**
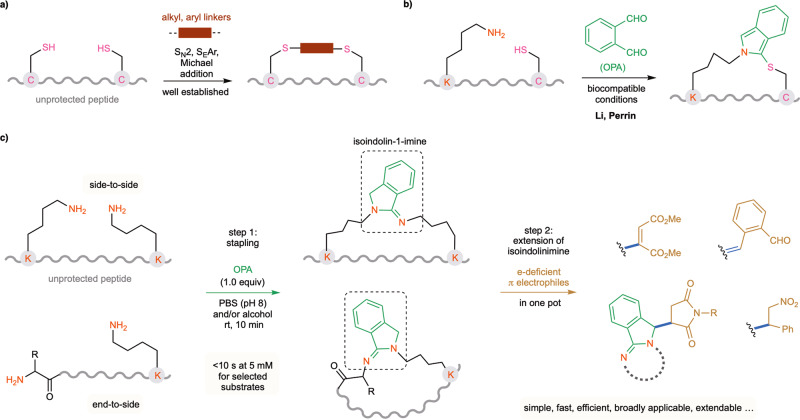
Stapling of unprotected peptides via non-Cys residues under mild aqueous conditions. **a** Common strategies for peptide stapling via two cysteine residues. **b** OPA-mediated peptide stapling via cysteine and lysine residues. **c** OPA-mediated peptide stapling via two amine groups (this work). C: cysteine, K: lysine. The thiol group of cysteine is marked in magenta. The amino group is marked in red. The linkers joining the two cysteines are marked in cayenne. The OPA reagent and isoindolinimine core are marked in green. The π electrophiles are marked in mocha. The linkages between isoindolinimine and electrophiles are marked in blue.

There is increasing recognition of the importance of peptides in drug discovery^1^. However, to modulate their structures and physiochemical properties, further modification of natural peptides or those generated by the standard synthetic protocols is usually required^2,3^. In this regard, the chemoselective crosslinking or stapling of two peptide handles has proven to be highly effective for the macrocyclization of peptides into more conformationally constrained versions^4–9^. Traditional approaches, which have been successful in the past, have involved bioorthogonal coupling reactions using noncanonical amino acid (AA) units, such as alkyne-azide cycloaddition and metal-catalyzed alkene metathesis. However, an approach that would involve the stapling of native peptides in their unprotected form would be much more desirable owing to the easy accessibility of such substrates and their high compatibility with biological systems^10–14^. Most of the research thus far has focused on the linking of highly reactive cysteine (Cys) residues (Fig. 1a)^15–21^. A commonly used peptide stapling technique involves the bis-alkylation or arylation of two Cys residues with linkers bearing two electrophilic sites. Recently, the groups of Li and Perrin independently reported a powerful method for the macrocyclization of native peptides between Cys and lysine (Lys) residues using a simple *ortho*-phthalaldehyde (OPA) reagent (Fig. 1b)^22,23^. While Cys has proven to be a very reliable anchor for peptide modification, it is often absent from the accessible peptides of interest and needs to be incorporated by additional synthetic manipulations. The current toolbox for stapling native peptides could be greatly expanded by exploring other common amino acid residues as potential cross-linking anchors^24–28^. Useful methods for such noncysteine stapling are still very scarce^5,29–31^. Here, we show that the primary amino groups from two AA residues can undergo a condensation reaction with OPA as the linker, forming a class of underexplored isoindolinimine structures under mild aqueous conditions (Fig. 1c). This chemoselective intramolecular OPA-2amines reaction resulted in the fast and efficient macrocyclization of native peptides in both N-end to side chain (end-to-side) and side chain to side chain (side-to-side) fashions under mild aqueous conditions with a broad substrate scope. Moreover, the resulting isoindolinimine linkage allows further extension via a one-pot process by reaction with various π electrophiles in high efficiency and selectivity, allowing secondary and even tertiary modification as well as the rapid construction of complex structures.

## Results

### Reinvestigation of the OPA-amine condensation reaction

**Fig. 2:**
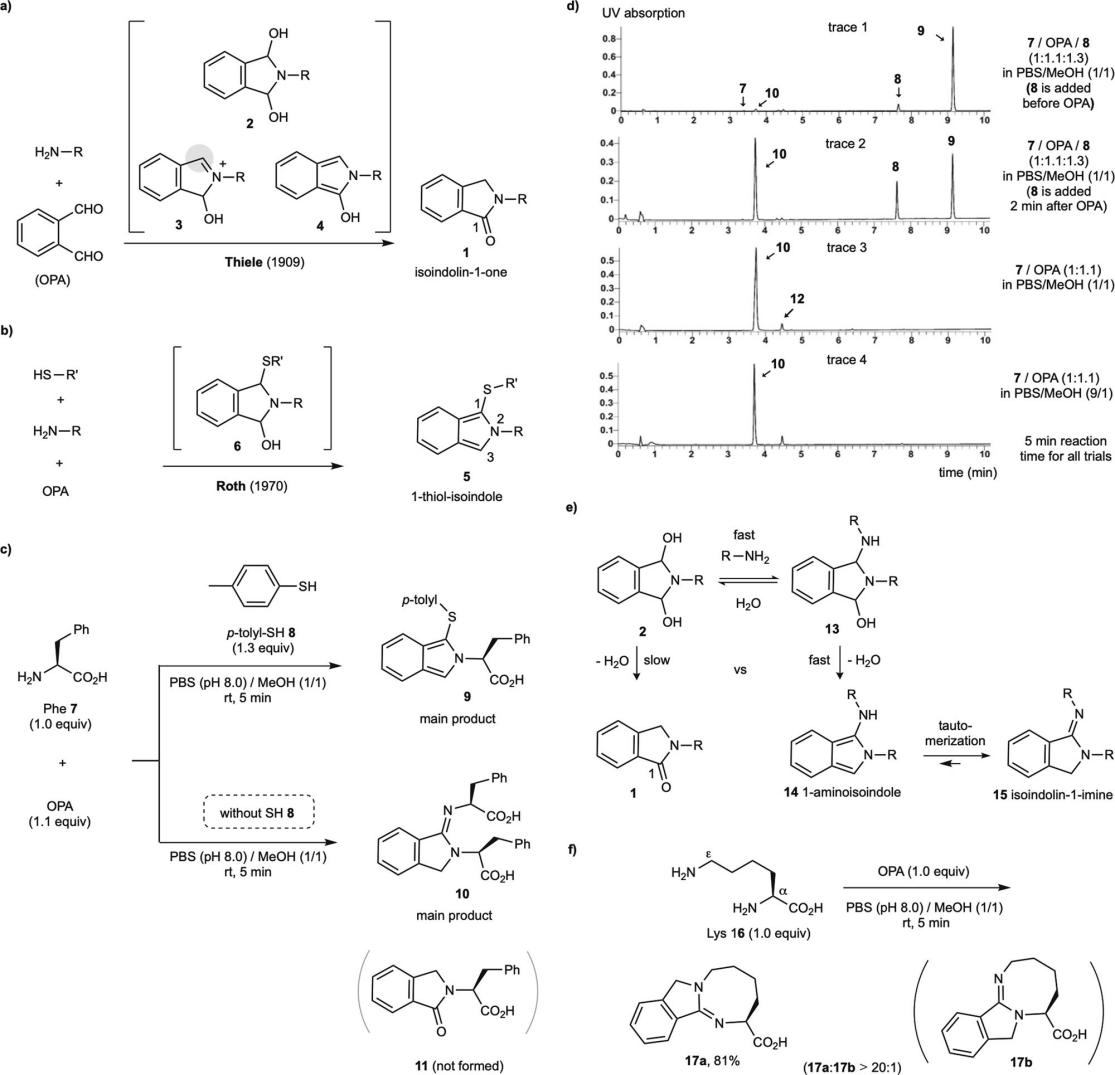
Reinvestigation of the OPA-amine condensation reaction under mild aqueous conditions in the presence and absence of thiol. **a** Two-component reaction (OPA-amine). **b** Three-component reaction (OPA-amine-thiol). **c** Comparison of reactions of amine and OPA with or without thiol. **d** LC-MS analysis of the reaction of Phe **7** and OPA with or without thiol **8**. Unprotected AA substrates, such as Phe **7**, gave a very weak UV signal on LC analysis. OPA (retention time: 2–3 min) also gave a weak UV signal on LC. **e** Proposed mechanism for the formation of isoindolin-1-imine. **f** Facile end-to-side cyclization of Lys with OPA (OPA-2amines). See Supplementary Figs 1–6 for the NMR-based structure assignment of **17a**. All reactions were conducted at a 0.05 mmol scale. Yields were based on an isolated product.

OPA has been used as a color and fluorescence indicator for sensing ammonia and amine compounds, such as α-amino acids (AAs) for over a century^32,33^. In 1909, Thiele was the first to report the formation of isoindolin-1-one **1** (also called phthalimidine) by the reaction of OPA with aniline in an organic solvent at room temperature (rt) (Fig. 2a)^34^. The reaction of OPA with amines proceeds by initial formation of the cyclic hemiaminal **2**, yielding iminium intermediate **3** after the loss of water. Several mechanisms, including protonation of isoindolinol intermediate **4**, a [1,3]-hydride shift, and the [1,3]-H sigmatropic rearrangement of **3**, have been proposed for the conversion of **3** to **1**^35–37^. The significance of the OPA-amine reaction was recently demonstrated by Li for the selective modification of Lys in peptides and proteins^38^. In the seminal discovery by Roth from 1970, it was found that a mixture of OPA, primary amine, and 2-mercaptoethanol quickly reacted in an alkaline aqueous medium to afford a strongly fluorescent product (Fig. 2b)^39^, which was later identified by Simons as the 1-thio-isoindole **5**^40^. Simons subsequent discovery that **5** reacted with dimethyl acetylenedicarboxylate (DMAC) to form a 1-thio-3-alkenylisoindole addition product with high efficiency hinted at the usefulness of this reaction type for further functionalization^41^. The three-component OPA-amine-thiol condensation reaction is believed to proceed through cyclic intermediate **6**, which undergoes dehydration to form **5**.

Owing to its rapidity and robustness, the Roth method has gained wide popularity to derivatize AA samples for chromatographic analysis. In addition, it has found usefulness to selectively modify Lys and Cys residues of proteins^42,43^. For example, Porttoghese developed an OPA-β-funaltrexamine conjugate as a fluorogenic affinity-labeling ligand of opioid receptors^42^. From these background works, Li and Perin developed the macrocyclization of unprotected peptides under biocompatible conditions by chemoselectively crosslinking Cys and Lys residues^22,23^. Furthermore, while there is much research mostly prior to 1990 describing the reaction of OPA with various partners in organic solvents, OPA chemistry under mild aqueous conditions that are potentially suitable for biomedical applications has not been vigorously investigated.

Recently, we discovered that the Roth method for HPLC analysis of AA could be repurposed for quantitative chiroptical sensing of AA using UV and circular dichroism (CD) spectroscopy^44^. As shown in Fig. 2c,d, the reaction of phenylalanine **7** (Phe, 1.0 equiv) with OPA (1.1 equiv) and *p*-toluenethiol **8** (1.3 equiv) in a phosphate-buffered saline (PBS) buffer (pH 8.0) and MeOH solvent mixture at room temperature (rt) quickly formed 1-thio-isoindole product **9** in high yield (see liquid chromatography (LC) trace 1). Besides MeOH, other polar organic solvents, such as EtOH, CH_3_CN, and DMSO, could also be used with success. The enantiomeric excess (ee) value could be determined from the UV and CD spectra of **9**, rather than by measuring its fluorescence signal. Similar to Roth’s original study^39^, the fluorescence signal of the reaction mixture was influenced by the addition sequence of the various reagents. If thiol **8** was incorporated a couple of minutes after the addition of OPA and Phe **7**, the signal was much weaker than if thiol is first added prior to incorporation of OPA. To the best of our knowledge, a detailed explanation for this observation has still not been offered^32,33^, so we set out to further investigate the OPA condensation reactions by LC-MS analysis. As mentioned, delayed addition of thiol **8** to the mixture of OPA and **7** in a 1:1 PBS buffer (pH 8.0) and MeOH solvent mixture led to reduced **9** and formation of the new isoindolinimine adduct **10**, formed from the reaction of one OPA and two Phe molecules (LC trace 2). Moreover, mixing 1.0 equiv of Phe **7** with 1.1 equiv of OPA in the absence of any thiol quickly and selectively formed **10** as the main product in 5 min (LC trace 3). A minor side product **12** with a molecular weight equivalent to the addition product of **10** and another molecule of OPA (double-OPA product) was generated in a trace amount, while no isoindolin-1-one **11** was detected. The mechanism for the formation of isoindolinimine likely starts with the reaction of a primary amine with **2**, forming aminal **13** (Fig. 2e). Intermediate **13** dehydrates to generate 1-aminoisoindole **14**, and then quickly tautomerizes to form isoindolin-1-imine **15**. If an amine is absent, then **2** slowly dehydrates to give **1**. As shown in Fig. 2f, Lys **16** (1.0 equiv) reacts with OPA (1.0 equiv) in a 1:1 PBS buffer/MeOH mixture to quickly generate cyclic product **17a** in excellent yield and regioselectivity (**17a**/**17b** > 20/1), with the structure confirmed by NMR analysis. Such chemoselectivity might be attributed to the intrinsically stronger nucleophilicity of the side chain ε amine group in Lys compared to the α NH_2_ group, which is deactivated by the electron-withdrawing carboxylate group. The slightly basic conditions and the MeOH co-solvent might have enhanced the formation of unprotonated Lys side chain in the reaction system^45^.

### End-to-side stapling of peptides by OPA-2amines condensation

**Fig. 3:**
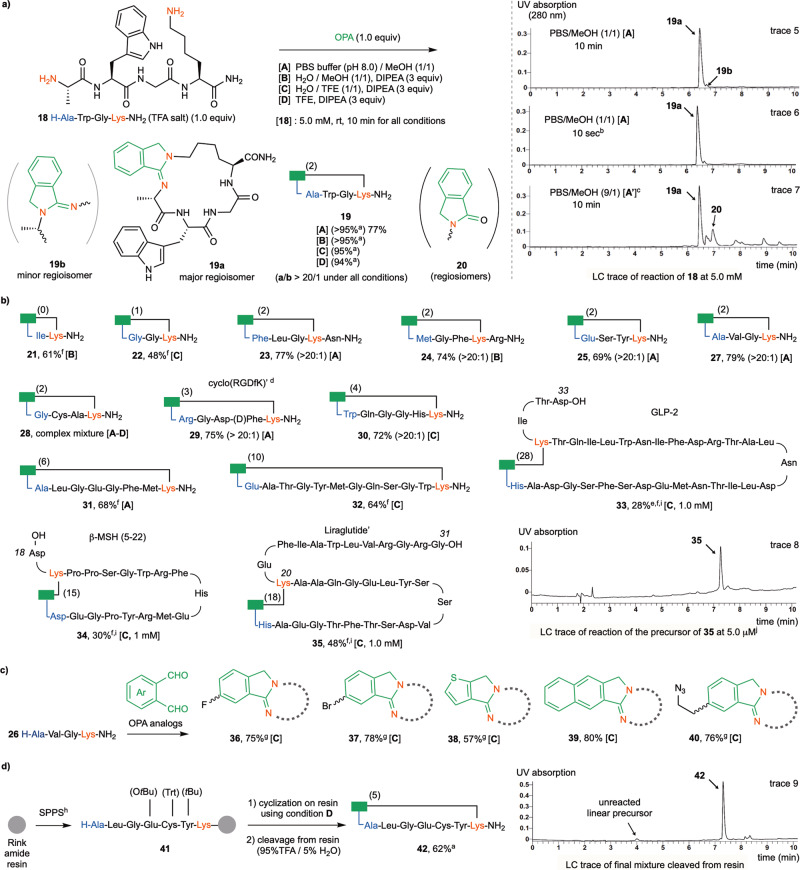
OPA-2amines reaction for end-to-side stapling of unprotected peptides. **a** Reaction of model substrate **18** with OPA. **b** Influence of spacing units and amino acid composition. **c** Cyclization of H-Ala-Val-Gly-Lys-NH_2_ **26** with OPA analogs. **d** Cyclization on solid phase. Yield is based on the HPLC isolated product of reaction conducted at 5.0 mM concentration and 0.001-0.05 mmol scale unless otherwise specified. The number of spacing AA units is shown in parentheses next to the green rectangle symbol that represents the isoindolinimine motif. The ratio of two regioisomers is shown in parentheses next to product yield. ^a^LC-estimated yield. ^b^A small aliquot of the reaction mixture was taken and quenched with mixed solvents of H_2_O/MeOH/HCO_2_H before subjection to LC-MS analysis. ^c^Condition **A’** is same as **A** except the ratio of buffer and MeOH. ^d^Cyclo(RGDfK) is a cyclic pentapeptide joined by five backbone amide bonds. ^e^The acyclic isoindolinone side product was formed in about 40% LC yield. ^f^The ratio of regioisomers was difficult to determine by LC. ^g^The product was obtained as a mixture of regioisomers. ^h^See Supplementary Figs 29 and 30 for details of SPPS on resin. ^i^Reaction was conducted at 1.0 mM for 10 min. ^j^Reaction was conducted at 5.0 μM for 10 min. Thr(ol): L-threoninol. The uncapped N-terminal AA units are marked in ocean blue, the lysine units and amino groups are marked in red, the OPA reagent and isoindolinimine core are marked in green.

Having established the condensation reaction of Lys as a proof of concept for the intramolecular condensation between OPA and two amino groups, we next investigated whether the OPA-2amines reaction can be applied in a macrocyclic fashion to crosslink the N-terminal amino group and the Lys side chain of linear peptides (Fig. 3a), easily prepared via solid-phase peptide synthesis (SPPS). We were gratified to find that the model substrate tetrapeptide H-Ala-Trp-Gly-Lys-NH_2_ **18** at a 5.0 mM concentration quickly reacted with OPA (1.0 equiv) in a 1:1 mixture of PBS (pH 8.0) buffer and MeOH at rt (condition [**A**]), generating cyclized product **19a** in 77% yield and with excellent regioselectivity (**19a**/**19b** > 20/1, see LC trace 5). To our surprise, LC-MS analysis indicated the reaction was completed in less than 10 seconds at 5 mM concentration (LC trace 6). The same reaction of **18** at 50 μM was completed within 2 min (see Supplementary Fig. 8). A small amount of side product (~10% based on LC estimation) featuring the condensation of **19a** with additional OPA molecule (double OPA product) was formed when the concentration of substrate was increased to 50 mM (see Supplementary Fig. 66). Little intermolecular dimerization product was formed even when the reaction concentration was increased to 100 mM. Notably, the pH value of the medium had a strong impact on the reaction. While the reactions gave very similar results at pH values between 8.0 and 10.0, the LC yield of **19a** slightly dropped to 83% at pH 7.0 and to 34% at pH 5.0 along with the significant increase of side product **20** under otherwise identical conditions. Little product was formed at pH below 3.0 (see Supplementary Fig. 68).

Interestingly, unlike in the reaction of Lys with OPA, lowering the MeOH content in the solvent mixture to a concentration such as 10% led to increased formation of the isoindolinone side product **20**, along with other unidentified side products (condition [**A’**], LC trace 7). Other alcohol co-solvents, such as EtOH and trifluoroethanol (TFE), were also effective for promoting the desired macrocyclization. The reaction with DMSO, DMF, CH_3_CN, dioxane, or THF as cosolvents gave a considerable amount of **20** and other side products (see Supplementary Fig. 63 for the detailed evaluation of organic cosolvents). The buffer could be substituted by adding diisopropylethylamine (DIPEA, 3 equiv, for neutralization of the residual TFA in peptide substrate) to a 1:1 mixture of H_2_O and MeOH, also efficiently generating **19a** (condition [**B**]). Alternatively, TFE could be used as an efficient cosolvent for peptide substrates with low solubility in methanol (condition [**C**]), although pure TFE was also a good solvent for the reaction (condition [**D**]). Mechanistically, we suspect that alcohols might facilitate the dehydration of aminal intermediate **13** to form **14** via solvent-bridged proton transfer (see Supplementary Fig. 65) for our proposed mechanistic model). In comparison to the 1-thio-isoindole motif such as in **5**, which is relatively prone to oxidation by air, the isoindolinimine moiety in compound **19a** proved to be very robust in aqueous solvents at rt under air atmosphere over a wide pH range (1-12). Heating **19a** in PBS buffer (pH 7.3) at 60 °C for 12 h gave little decomposed product. However, significant amounts of the acyclic isoindolinone side product (>30%) was formed when **19a** was heated in the same buffer at 100 °C for 2 h (see Supplementary Fig. 69 for details).

The OPA-2amines-mediated end-to-side macrocyclization was efficient for peptide substrates of a wide range of ring sizes and AA compositions, affording the products in excellent regioselectivity in 10 min (Fig. 3b). Most of the reactions were completed within 2 min. Two examples that demonstrate the flexibility permitted by the reaction with regards to the number of spacing AA units are seen in the formation in high yield of **21** bearing no spacing AA units and **35** bearing more than 10 AA units between the N-terminus and the Lys residue. Except for Cys, all unprotected proteinogenic AA units, such as Arg (**24**), His (**30**), Trp (**33**), Tyr (**24**), Ser (**24**), and Glu (**31**), were well tolerated. D-AA units, such as D-Phe (**28**), could also be incorporated. Notably, the reaction of substrates containing Cys resulted in a complex mixture of products (e.g., **28**). Except for Pro, which bears a secondary amino group, a variety of proteinogenic AAs can be placed at the N-terminus. The conversion of large peptide substrates was more favorable with TFE as a cosolvent (condition [**C**]), owing to its superior solubilizing ability compared to MeOH.

We next set out to synthesize crosslinked peptides with potential biomimetic activity or active medical properties. For example, compound **29** was prepared in excellent yield to mimic the integrin inhibitor cyclo(RGDfK). The reaction of the 18-mer human β-melanocyte-stimulating hormone (β-MSH 5-22) afforded cyclized product **34** in 30% yield. An analog of the diabetes drug Liraglutide (without the lipophilic conjugate on the side chain of Lys20) gave **35** in 48% yield at 1 mM reaction concentration. Based on LC analysis, a slightly cleaner transformation was observed when the reaction was conducted at 5 μM (LC trace 8). Finally, the reaction of 33-mer glucagon-like peptide 2 (GLP-2), featuring 28 spacing AA units, gave **33** in 28% yield, along with acyclic isoindolinone side products. Using peptide substrate H-Ala-Val-Gly-Lys-NH_2_ **26**, the macrocyclization reaction was also amenable to a range of OPA analogs (see **36**-**40**, Fig. 3c). Importantly, using **41** as a model compound, we showed that OPA-2amines peptide stapling can be performed in conjunction with solid-phase peptide synthesis (Fig. 3d). Treatment of partially protected peptide substrate **41** on resin with OPA in TFE (condition [**D**]) followed by TFA-mediated deprotection and cleavage gave **42** bearing a free Cys residue in excellent conversion and selectivity (LC trace 9).

### Side-to-side stapling of peptides by OPA-2amines condensation

**Fig. 4:**
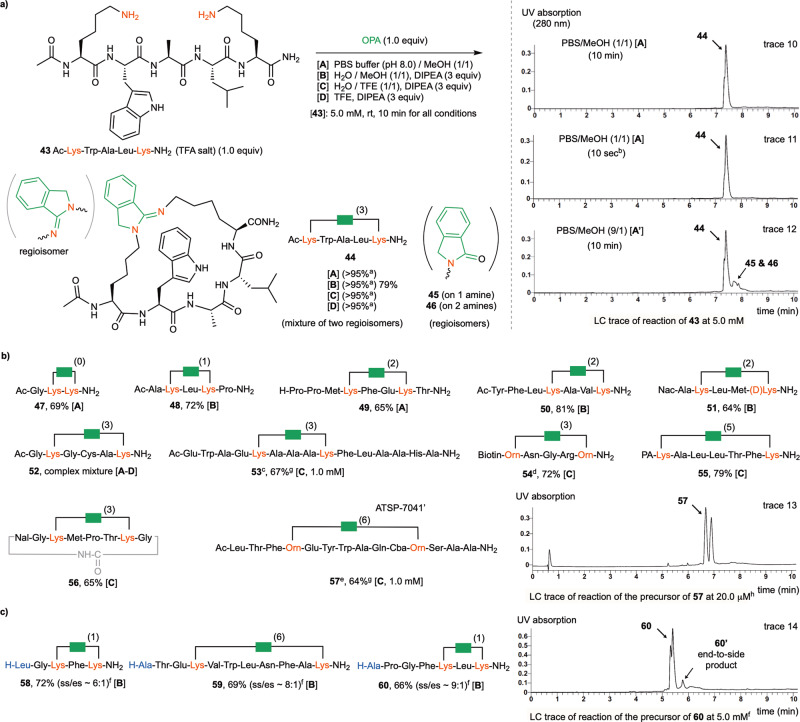
OPA-2amines reaction for side-to-side stapling of peptides. **a** Model reaction of **43**. **b** Influence of spacing units and amino acid composition. **c**) Reaction of substrates with free N-terminal NH_2_. Yield is based on HPLC isolated product of reactions conducted at 5.0 mM concentration and 0.01-0.05 mmol scale unless otherwise specified. All products were obtained as a mixture of regioisomers. ^a^ LC-estimated yield. ^b^A small aliquot of the reaction mixture was taken and quenched with mixed solvents of H_2_O/MeOH/HCO_2_H before subjection to LC-MS analysis. ^c^Adaption from an α helical sequence of RNase A. ^d^Biotin was attached to the N-terminus. ^e^Adaption from peptide ATSP-7041. ^f^ The structures of the major side-to-side (ss) stapled products were confirmed by comparison with the samples prepared from properly protected substrates, see Supplementary Fig. 48 for details. The minor peaks were assigned as the end-to-side (es) products according to molecular weight and their structures have not been vigorously confirmed. The ss/es ratio was estimated by LC-MS analysis. ^g^Reaction was conducted at 1.0 mM for 10 min. ^h^Reaction was conducted at 20.0 μM for 10 min. Nal: L-1-Naphthylalanine. PA: picolinic acid. The uncapped N-terminal AA units are marked in ocean blue, the lysine units and amino groups are marked in red, the OPA reagent and isoindolinimine core are marked in green.

As shown in Fig. 4a, the OPA-2amines reaction could also enable highly efficient side chain to side chain (side-to-side) stapling of linear peptides via two Lys side chains under the standard conditions already described in end-to-side stapling. For example, the reaction of pentapeptide **43** with an Ac-protected N-terminus with OPA (1.0 equiv) in 10 min formed the cyclic product **44** in excellent yield (LC trace 10). Notably, the product was obtained as a mixture of two regioisomers in a close ratio due to the similar reactivity of the two Lys side chains. As seen in the end-to-side stapling of **17**, stapling of **43** at 5 mM can be completed within 10 seconds (LC trace 11). Stapling of **43** at 50 μM for 2 min gave almost identical results (see Supplementary Fig. 32). Stapling of **43** also worked well at relatively high concentration, such as 50 mM, whereas a small amount of double-OPA side product (~5% based on LC analysis) was formed when the concentration was further increased to 100 mM (see Supplementary Fig. 67). Similar to end-to-side stapling, using alcohol as a cosolvent facilitated the desired macrocyclization. A considerable amount of acyclic isoindolinone side products **45** and **46** were formed when the ratio of MeOH vs buffer was lowered from 1:1 to 1:9 (condition [**A’**], LC trace 12).

Substrates with a wide range of spacing units between the two Lys residues were well tolerated, and all proteinogenic AAs except for Cys are compatible (Fig. 4b). The reaction was adaptable to various unnatural AA units such as ornithine (Orn, **57**) and capping groups such as biotin (**54**) could be incorporated. As shown by **49**, the secondary amine group in N-terminal Pro remained unaffected by the reaction. The utility of the side-to-side peptide stapling technique was demonstrated by the synthesis of various biomimetic analogs. For example, the reaction of a 15-mer peptide sequence derived from RNase A gave **53** in excellent yield^46^. Moreover, the macrocyclization of a 14-mer sequence derived from ATSP-7041^47^, a peptide inhibitor of the p53-MDM2 interaction, at 1 mM gave **57** in 64% yield via side-to-side stapling of two Orn side chains. As shown in LC trace 13, slightly cleaner transformation was observed when the reaction were conducted at 20 μM concentration. The macrocyclization reaction can be used to generate complex bicyclic products as exemplified by the reaction of **56**, a macrolactam peptide substrate with two Lys, which undergoes cyclization to afford the bicyclic product in excellent yield and selectivity. As shown by **58**-**60**, the stapling of substrates bearing two Lys side chains and unblocked N-terminal amine groups proceeded with high side-to-side vs end-to-side selectivity, indicating the higher reactivity of the Lys side chain than the N-terminus in our reaction system (Fig. 3c)^45^.

### Further extension of isoindolinimine products

**Fig. 5:**
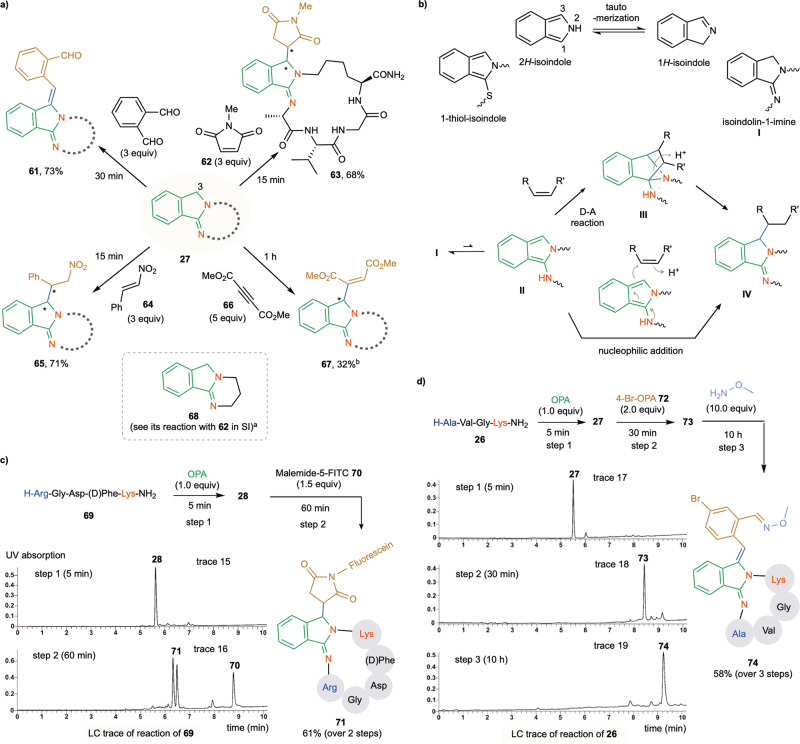
Facile extension of stapled product with electron-deficient π electrophiles. All reactions were conducted under condition [**C**] (H_2_O/TFE (1:1), DIPEA, rt) at 5.0 mM concentration. **a** Extension of **27**. Isolated yield over two steps in one pot. Stereochemistry of the double modification products has not been determined. **b** Possible reaction pathways of the isoindolin-1-imine moiety. **c** Fluorescent labeling of **69** via double modification in one pot. **d** Triple modification of **25** via sequential additions in one pot. ^a^ See Supplementary Figures 51–54 for a detailed analysis of the reaction of model substrate **68** with **62**. The uncapped N-terminal AA units are marked in ocean blue, the lysine units and amino groups are marked in red, the OPA reagent and isoindolinimine core are marked in green, and the linkages between isoindolinimine and electrophiles are marked in blue.

In addition to the excellent stability of the macrocyclic isoindolinimine products in an aqueous medium at rt over a wide range of pH values, they could also undergo further chemoselective reaction with certain electron-deficient π electrophiles under the same stapling conditions, enabling the highly efficient and facile construction of complex structures from simple linear precursors in a one-pot fashion (Fig. 5a). For example, when **26** was reacted with a large excess of OPA (3.0 equiv) in a H_2_O/TFE mixture (condition [**C**]) for 30 min, the double-OPA macrocyclization product **61** was cleanly formed in 73% yield. However, the success of the condensation reaction depends strongly on the nature of the aldehyde reaction partner, and other aldehydes, such as benzaldehyde, methylglyoxal, and glyoxylic acid, gave little **61**-like product (see Supplementary Fig. 50). Similar to the reactivity of the 1-thio-isoindole product generated by the OPA-amine-thiol reaction, **26** can likewise undergo further addition reactions with appropriate electrophiles. For example, treatment of **27** with N-methyl maleimide **62** (3.0 equiv)^22^ for 15 min gave the C_3_-alkylation product **63** in 68% yield. Alkyne DMAC **66** can act in a manner analogous to maleimide for the one-pot addition reaction, but the reactivity towards DMAC strongly depends on the reaction partner. Hence, whereas **27** reacts sluggishly giving **67** in low yield, under the same conditions the linear substrate H-Phe-Leu-Gly-Lys-Asn-NH_2_ reacts to give the addition product in good yield (67%, see Supplementary Fig. 59 for details). Moreover, β-nitrostyrene **64** is also an efficient acceptor for the sequential reaction using **27** as the substrate, forming **65** in high yield. It should be noted that similar results were obtained for this one-pot procedure using either condition [**A**] or [**B**]. Reactions of **27** with methyl acrylate, vinyl sulfones, and 4-phenyl-3H-1,2,4-triazoline-3,5(4H)-diones gave little desired products, whereas the reaction with aryl diazonium salts gave a complex mixture (see Supplementary Fig. 62).

In general, isoindoles are more reactive than indoles. The plain isoindole predominately exists in the 2*H*-isoindole form, which can tautomerize to 1*H*-isoindole at very small concentration via equilibrium (Fig. 5b)^48^. However, the ratio of the two tautomers can vary depending on the substitutions on isoindoles. In contrast with the 1-thiol-isoindole core generated by the OPA-thiol-amine reaction, the isoindole core generated by the OPA-2amines reaction predominantly exists in the isoindolin-1-imine form **I**, which can be viewed as a variant of 1*H*-isoindole. Previously studies have shown that 2*H*-isoindoles can undergo Diels-Alder (D-A) type of reaction with dienophiles across the C1-C3 and electrophilic substitution reaction at C1/C3^49–51^. We propose that isoindolinimine **I** can tautomerize to 1-aminoisoindoline **II**, which is generated in small concentration but likely much more reactive that **II**. **II** can undergo Diels-Alder reaction with dienophile such as maleimides to generate cycloadduct **III**, which immediately rearranges to give the final C3 substitution product **IV** possibly due to the strain around the C1 aminal center of **III**^50^. NMR and LC-MS-based analysis of the model reaction of **68** with **62** did not identify any stable cycloadduct intermediate in our hands (see Supplementary Fig. 54 for details). Alternatively, **II** can undergo nucleophilic addition to alkene electrophiles to directly give **IV**. Nucleophilic addition of **II** to the aldehyde group of OPA followed by dehydration is likely responsible for the formation of the corresponding C3 condensation products (see **61**)^51^.

As outlined in Fig. 5c, sequential addition of OPA and FITC-conjugated maleimide **70** to the RGD-containing sequence **69** gave the fluorescently labeled product **71** in 61% yield over two steps (LC trace 16). The usefulness of our one-pot approach could even be extended to a three-step sequential addition of OPA, 4-bromo-OPA **72**, and methoxyamine to substrate **26**, giving the triple-modified product **74** in good yield (Fig. 5d).

Through reinvestigating the classical OPA-amine-thiol condensation chemistry, we found that OPA quickly reacted with two primary alkyl amino groups to selectively form isoindolinimine products, whose chemical reactivity has been rarely explored. The OPA-2amines-mediated macrocyclization reaction provides a practical and broadly applicable method for the fast stapling of unprotected peptides via the reaction of the amino groups from two AA residues and an OPA or OPA analogs in both side-to-side and end-to-side fashions under mild conditions. Moreover, the resulting isoindolinimine linkage can undergo further reaction with electron-deficient π electrophiles under the same cyclization conditions in one pot. Overall, the reported stapling methodology and one-pot sequential addition reactions offer a powerful strategy to selectively modify unprotected peptides and construct diverse macrocyclic structures with readily accessible peptide precursors and inexpensive reagents.

## Methods

### Typical procedure for both end-to-side and side-to-side peptide stapling

To a solution of linear peptide precursor **18** (32.7 mg, 0.05 mmol, 1.0 equiv) in 10.0 mL of PBS buffer (pH 8.0) / MeOH (1:1), OPA was added (1.0 equiv, 50 μL of 1 M stock solution in MeOH). The reaction mixture was stirred in an air atmosphere for 10 min at rt. The reaction mixture was then purified by semipreparative HPLC in two batches with a reverse-phase C18 column (H_2_O and MeCN with 0.1% HCO_2_H as eluents) to give the product **19a** as a yellowish powder after lyophilization (23.2 mg, 77%).

## Supplementary information

Supplementary Info

## Data Availability

Detailed synthetic procedures, additional control experiments, compound characterization, LC-MS trace, and NMR spectra are available within the Article and its Supplementary Information.
