# Supplementary material for: Extendable stapling of unprotected peptides by crosslinking two amines with o-phthalaldehyde
Source: Nat Commun. 2022 Jan 14;13:311. doi: 10.1038/s41467-022-27985-7 (PMC8760283; doi:10.1038/s41467-022-27985-7)
Supplement: Supplementary file 1 — Supplementary Info [file 41467_2022_27985_MOESM1_ESM.pdf]

Supplementary Information

Extendable Stapling of Unprotected Peptides by Crosslinking Two Amines with *o*-Phthalaldehyde

Bo Li[1], Lan Wang[1], Xiangxiang Chen[1], Xin Chu[1], Hong Tang[1], Jie Zhang[2], Gang He[1], Li Li[2]*, and Gong Chen[1]*

[1]State Key Laboratory and Institute of Elemento-Organic Chemistry, College of Chemistry, Nankai University, Tianjin 300071, China

[2]Beijing Key Laboratory of Active Substances Discovery and Druggability Evaluation, Institute of Materia Medica, Chinese Academy of Medical Sciences, Peking Union Medical College, Beijing 100050, China

Supplementary Information **S1**

1. Supplementary Methods **S2**

1.1 Reagents and instruments **S2**

1.2 Preparation of linear peptides **S2**

1.3 General procedure for OPA-2amines reactions **S3**

2. Supplementary Discussion **S3**

2.1 Characterization of OPA-2amines products **S3**

2.2. Facile extension with electron deficient π electrophiles **S48**

2.3 Evaluation of organic cosolvents, concentration and pH **S60**

2.4 Stability test of the cyclic peptides via OPA-2amines reaction **S67**

3. NMR spectra **S69**

4. Supplementary References **S83**

1. Supplementary Methods

1.1 Reagents and Instruments

Fmoc-amino acids and coupling reagents (DIC and Oxyma) were purchased from Shanghai Haohong Scientific Co. Ltd. Rink amide MBHA resin and 2-Cl-Trt resin were purchased from GL Biochem. PBS buffer (R&D Systems) and TFE (99.9%, Energy Chemical) were used in the stapling reaction. NMR spectra were recorded on Bruker AVANCE AV 400 instruments and Bruker Avance 600 MHz equipped with cryoprobe. UPLC-MS analyses were performed with a Dionex UltiMate 3000 connected to a thermo scientific MSQ PLUS mass spectrometer using Thermo Scientific Hypersil GOLD C18 (1.9 µm, 2.1 × 100 mm) or Agilent TC-C18 (5 µm, 4.6 × 250 mm). Linear gradients using A: H_2O (0.1% HCOOH) and B: MeCN (0.1% HCOOH) over varying periods of time. High-resolution mass spectra (HRMS) were recorded on a Thermo Q Exactive Focus using ESI. Semi preparative HPLC was carried out on a Dionex UltiMate 3000 using a Thermo Scientific Hypersil GOLD C18 (5 µm, 21.2 × 150 mm) preparative column. Linear gradients using A: H_2O (0.1% HCOOH) and B: MeCN (0.1% HCOOH) over varying periods of time. Peptide centrifugation was performed by DM0412 low speed centrifuge purchased from DLAB Scientific Co., Ltd. Peptide freeze drying was achieved by means of VirTis/SP SCIENTIFIC BenchTop Pro.

1.2. Preparation of linear peptide

Solid-phase peptide synthesis

Unless otherwise noted, linear peptides were assembled on an Automated microwave peptide synthesizer (Liberty Blue™, CEM) using Rink Amide MBHA or 2-Cl Trt resin. Fmoc-protected AAs (5.0 equiv), DIC (5.0 equiv) and Oxyma (5.0 equiv) were used in each coupling cycle. Deprotection of Fmoc was conducted by treatment with 20% piperidine/DMF for each cycle. Capping of the N-terminus can be achieved on the Synthesizer via acetylation (acetic anhydride) or coupling of other carboxyl compounds (picolinic acid, biotin, naphthoic acid, etc.).

Cleavage of linear peptides from resin

The dry Rink Amide MBHA and 2-Cl-Trt resin was treated with a cocktail of TFA/TIPS/H_2O

(95/2.5/2.5, v/v/v) for 2 hours (4 hours for the large-sized peptides containing more than 15 AAs). The solvents were dried under N_2 or Ar stream, after which the cold diethyl ether was added to precipitate the crude peptide. After centrifugation the supernatant was taken out and purification of peptides was conducted by semi-preparative HPLC if necessary.

1.3 General procedure for OPA-2amines reaction

Unprotected peptide substrates were dissolved in the mixed solvents to give a clear solution. OPA (1.0 equiv, stock solution) was added to the solution at room temperature and stirred for 10 min. A small aliquot was taken out and quenched with the mixed solvents of MeOH/H_2O/HCO_2H before subjection to UPLC-MS analysis. (For all experiments, OPA was freshly prepared as MeOH or TFE stock solution to use)

Condition A: PBS buffer (pH=8.0, 0.2 M)/MeOH=1:1 as solvent.

Condition B: H_2O/MeOH=1:1 as solvent, DIPEA (3.0 equiv) as base. Additional DIPEA was added when the peptide substrates containing protonated basic residues such as Arg, His.

Condition C: H_2O/TFE=1:1 as solvent, DIPEA (3.0 equiv) as base. Additional DIPEA was added when the peptide substrates containing protonated basic residues such as Arg, His.

Condition D: TFE as solvent, DIPEA (3.0 equiv). This condition was also used for on-resin cyclization.

2. Supplementary Discussion

2.1 Characterization data of OPA-2amines products

Elucidation of the isoindolinimine structure

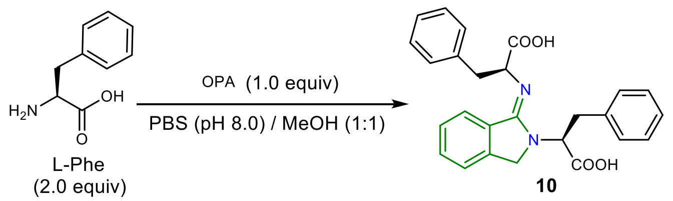

To a clear solution of L-Phe (2.0 equiv, 0.1 mmol, 16.5 mg) in 3 ml of MeOH-PBS buffer (pH = 8.0)(1:1), OPA (1.0 equiv, 0.05 mmol) was added at room temperature. After being stirred for 5 min, the reaction mixture was directly subjected to semi-preparative HPLC to give product **10** as a yellowish powder after freeze-drying.

^{1}H NMR (500 MHz, Methanol-d_4) δ 7.83 (d, J = 8.0 Hz, 1H), 7.58 (t, J = 7.5 Hz, 1H), 7.43 (d, J = 7.5 Hz, 2H), 7.35 (d, J = 7.5 Hz, 2H), 7.26 (d, J = 7.0 Hz, 2H), 7.19 – 7.11 (m, 5H), 7.08 (t, J = 7.5 Hz, 1H), 4.97 (dd, J = 9.0, 3.5 Hz, 1H), 4.68 (m, 1H), 4.63 – 4.55 (m, 1H), 4.15 (m, 1H), 3.53 (dd, J = 14.0, 11.5 Hz, 1H), 3.47 (dd, J = 14.0, 3.5 Hz, 1H), 3.40 (dd, J = 14.0, 5.0 Hz, 1H), 3.21 (dd, J = 14.0, 9.0 Hz, 1H). **^{13}C NMR (100 MHz, Acetic Acid-d4)** δ 173.68, 172.18, 162.15, 143.41, 135.99, 135.93, 133.75, 129.56, 128.86, 128.80, 128.69, 128.40, 127.19, 127.12, 126.75, 125.84, 123.30, 61.54, 59.71, 54.77, 38.16, 34.83.

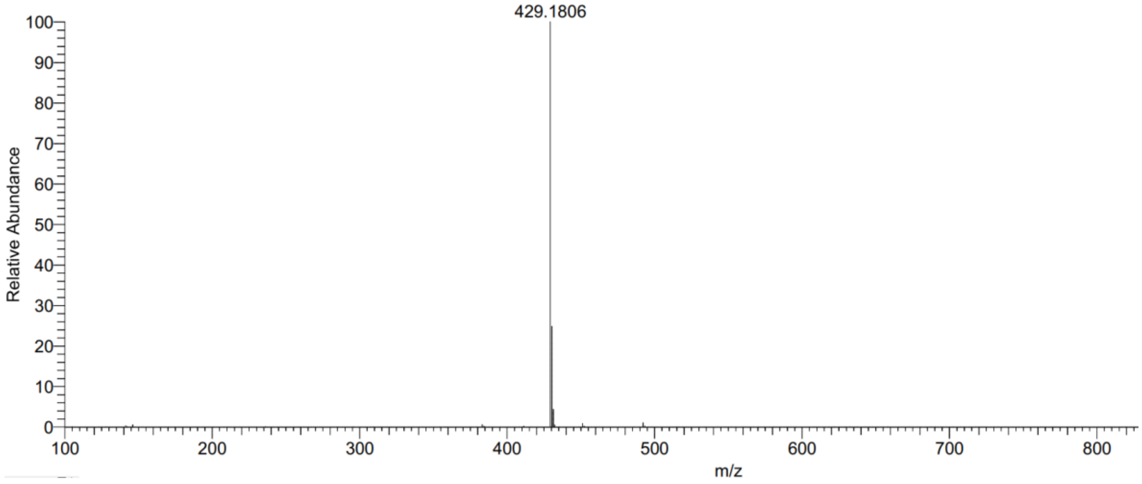

HRMS (ESI): calcd. for $C_{26}H_{25}N_2O_4$ $[M+H]^+$ = 429.1809; found 429.1806.

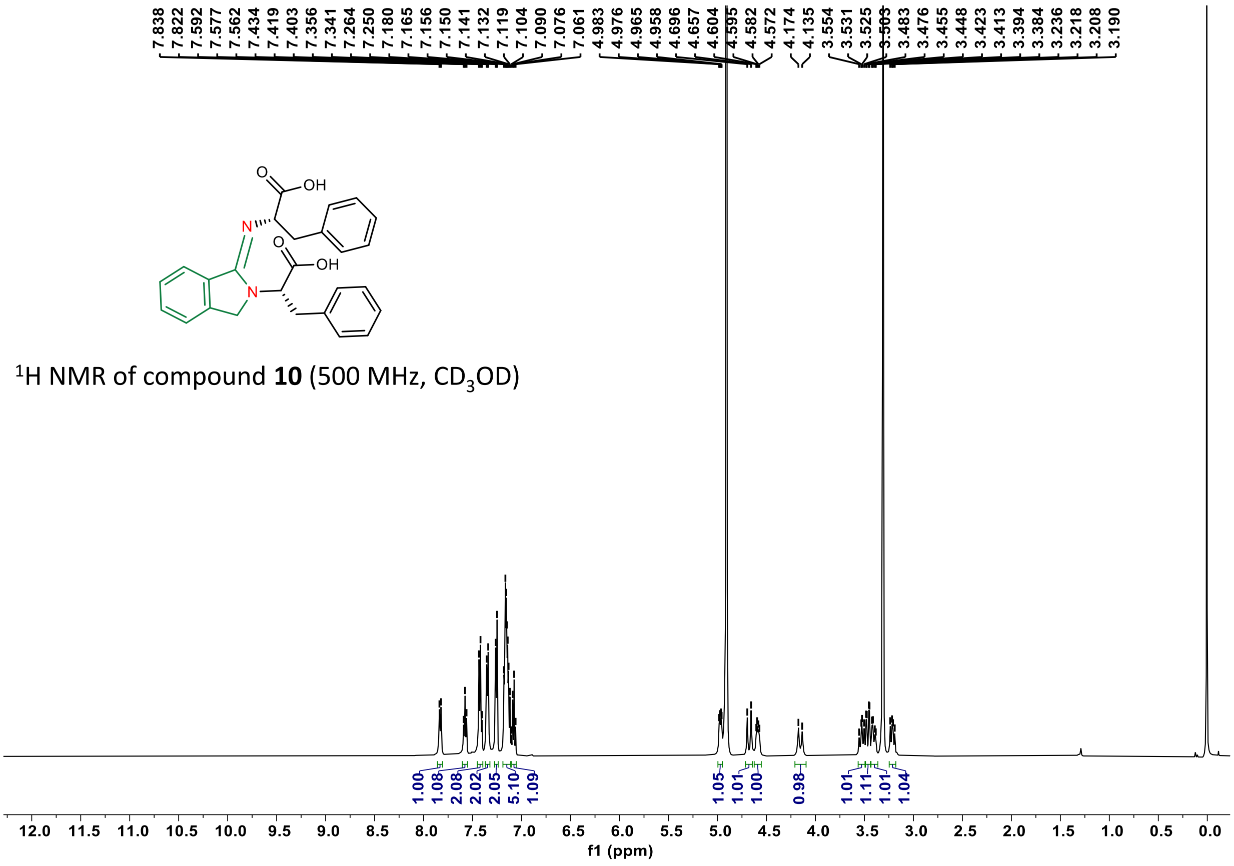

^{1}H NMR of compound **10** (500 MHz, CD_3OD)

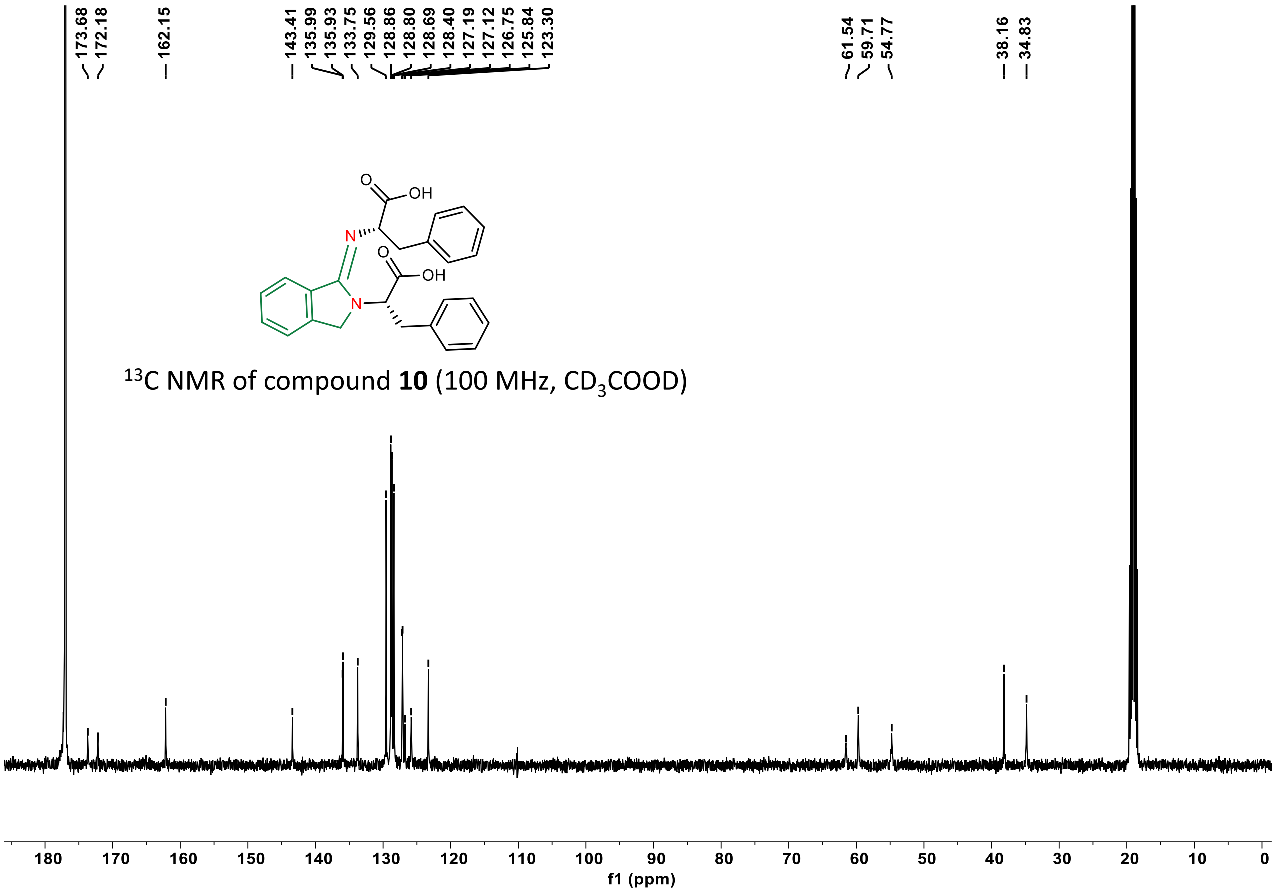

Supplementary Fig. 1. ^{1}H & ^{13}C NMR spectra of compound **10**.

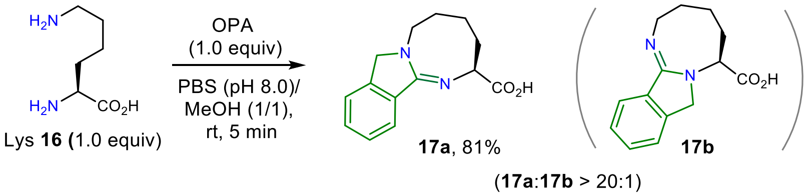

^{1}H NMR (600 MHz, DMSO-d_6) δ 7.94 (d, J = 7.8 Hz, 1H), 7.77-7.70 (m, 2H), 7.63-7.60 (m, 1H), 5.09–4.88 (m, 2H), 4.51–4.40 (m, 2H), 3.77–3.68 (m, 1H), 2.35-2.26 (m, 1H), 2.08-1.98 (m, 1H), 1.94-1.85 (m, 1H), 1.75-1.59 (m, 2H), 1.49-1.38 (m, 1H). **^{13}C NMR (150 MHz, DMSO-d_6)** δ 170.3, 157.8, 141.2, 132.9, 129.2, 128.6, 123.3, 121.6, 58.8, 55.1, 45.7, 34.2, 28.6, 21.7.

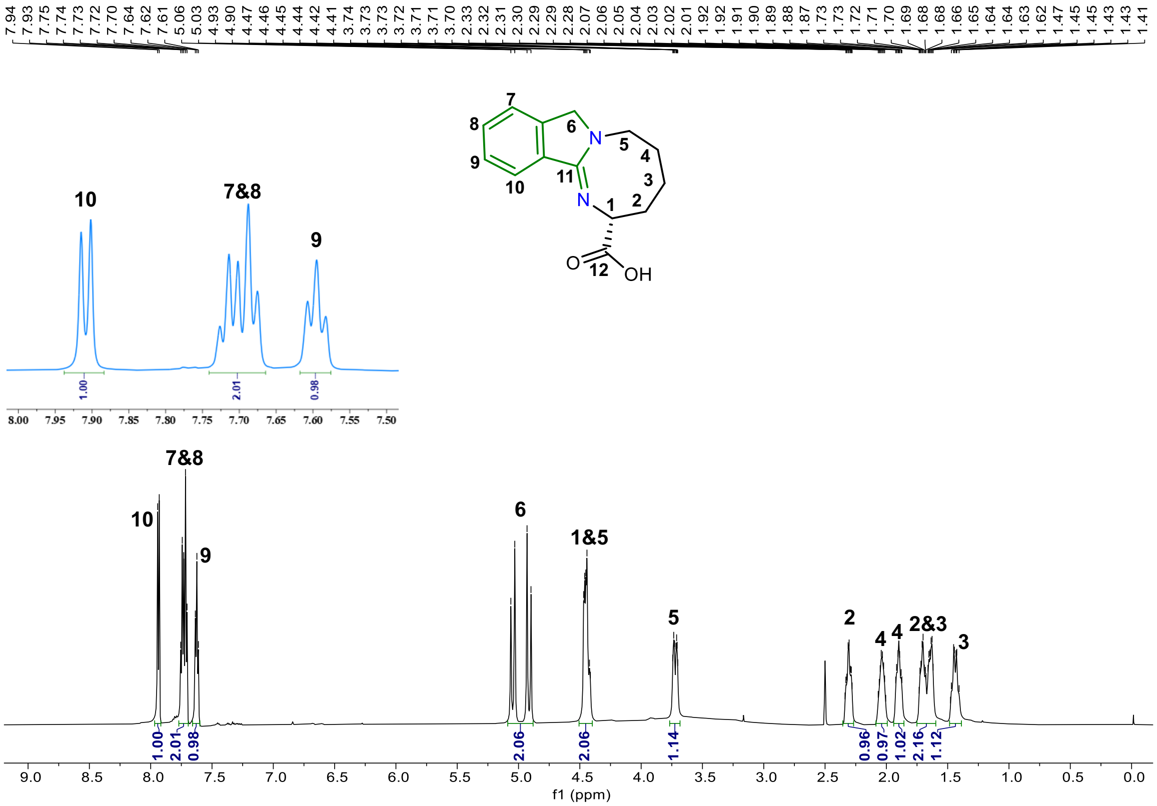

Supplementary Fig. 2. ^{1}H NMR spectrum of compound **17a**.

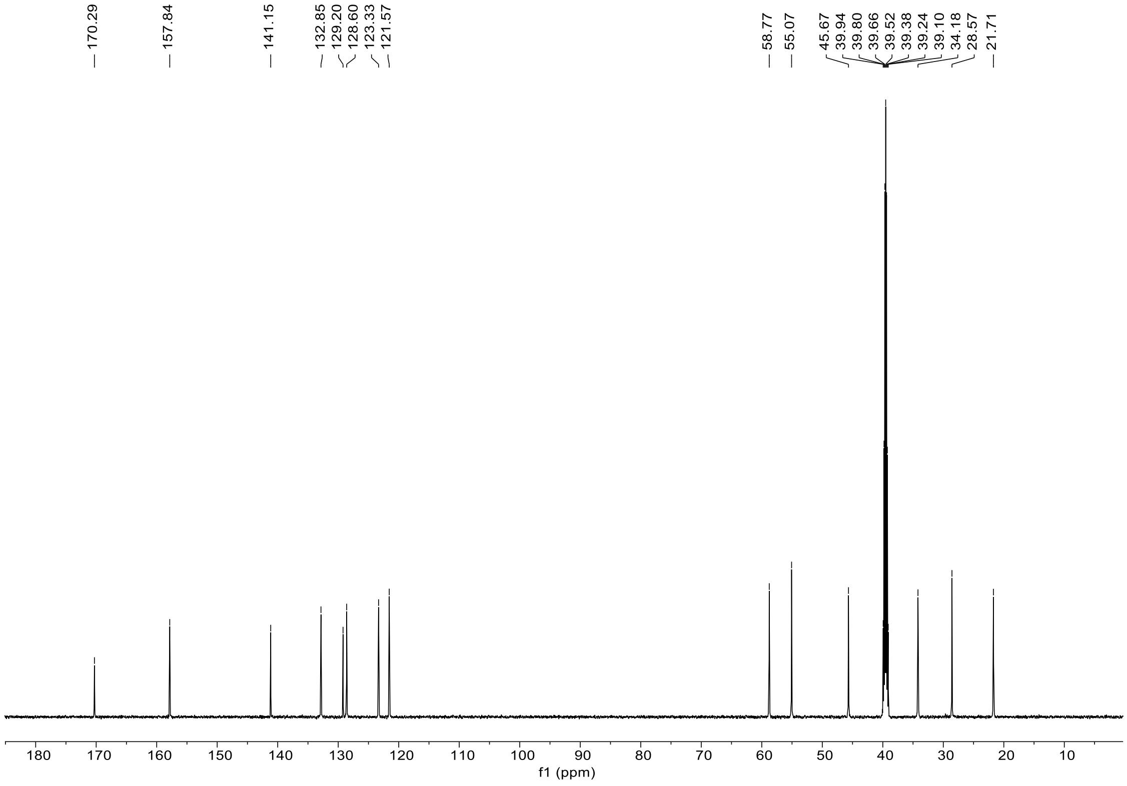

Supplementary Fig. 3. ^{13}C NMR spectrum of compound **17a**.

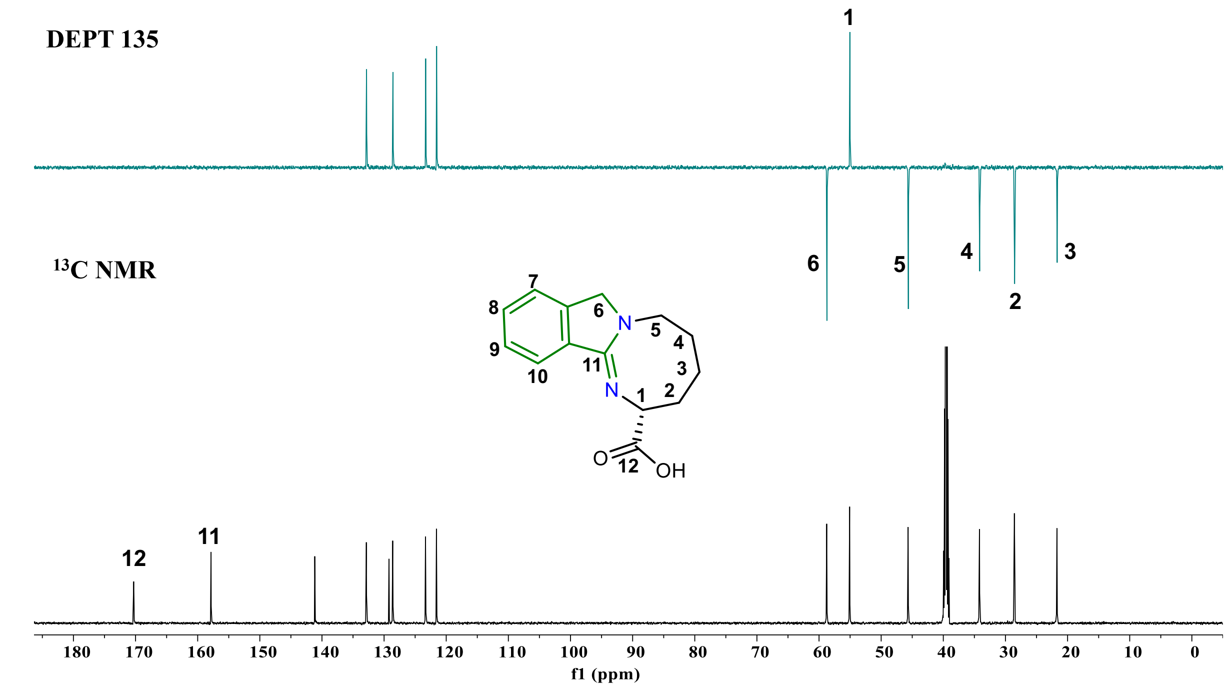

Supplementary Fig. 4. DEPT & ^{13}C NMR spectra of **17a**.

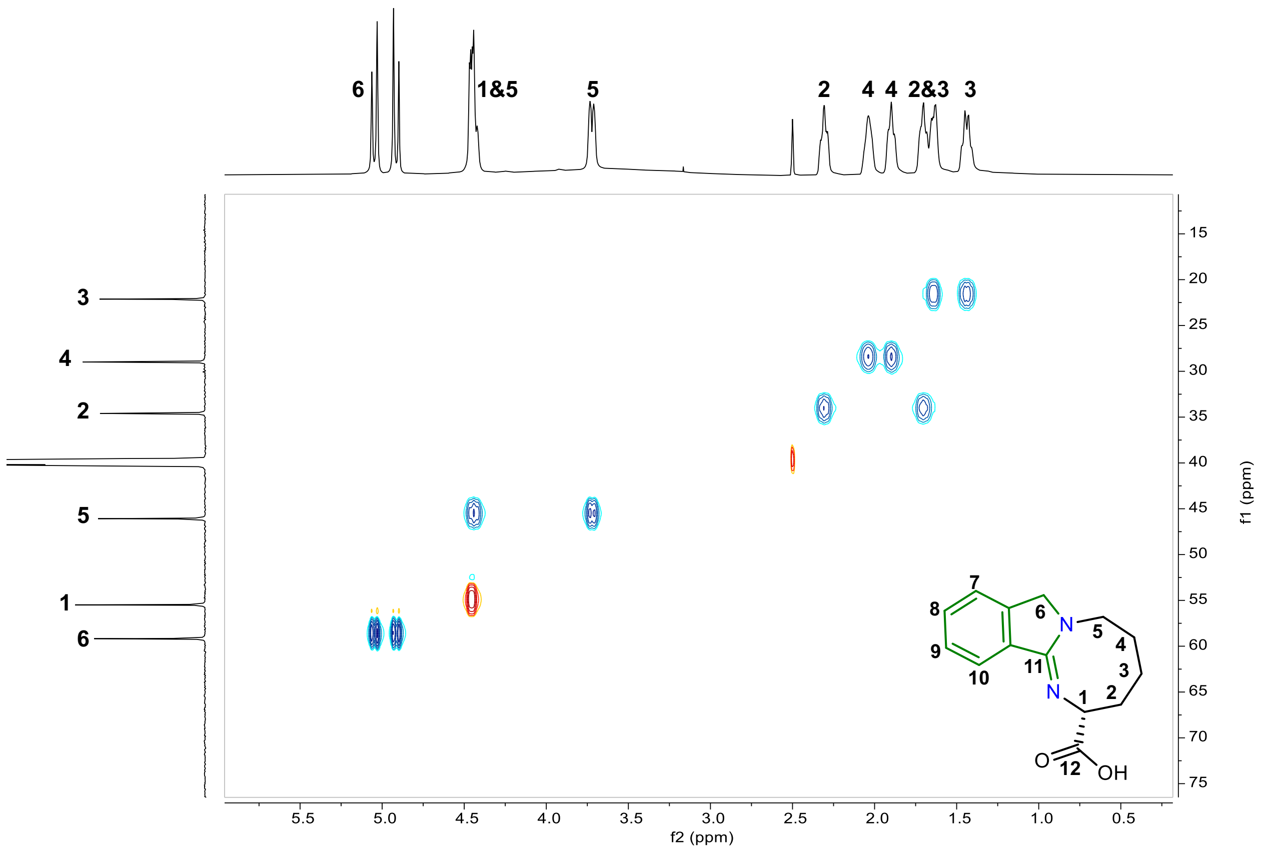

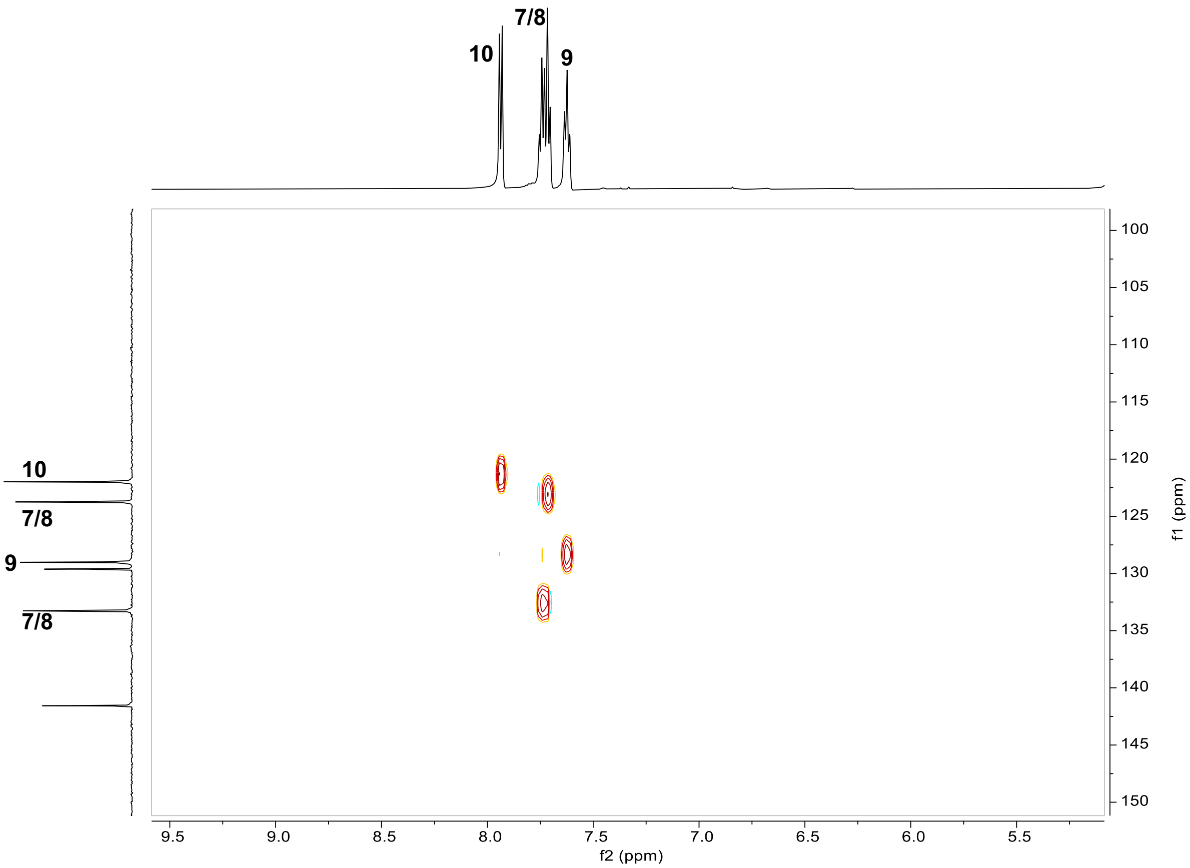

Supplementary Fig. 5. HSQC spectrum of **17a**.

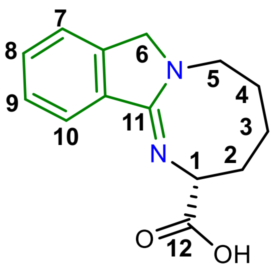

**Position**	**δ_C, type**
**1**	55.1, CH
**2**	28.6, CH_2
**3**	21.7, CH_2
**4**	34.2, CH_2
**5**	45.7, CH_2
**6**	58.8, CH_2
**7&8**	123.3/132.9, CH
**9**	128.6, CH
**10**	121.6, CH
**11**	157.8, C
**12**	170.3, C

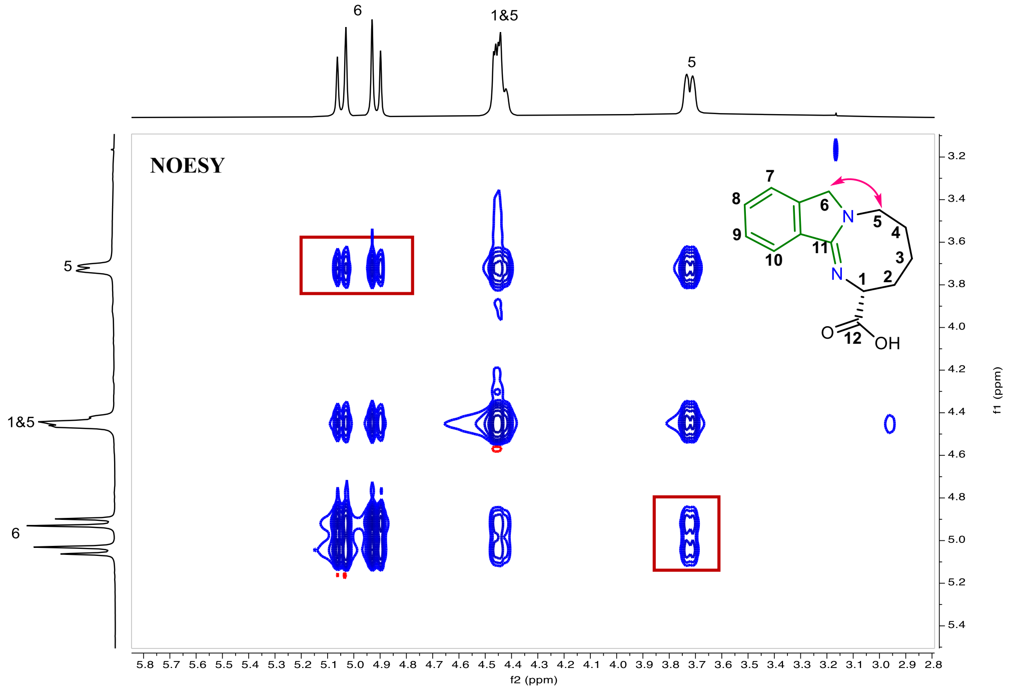

Supplementary Fig. 6. NOESY spectrum of **17a**.

Characterization for head-to-side macrocyclization

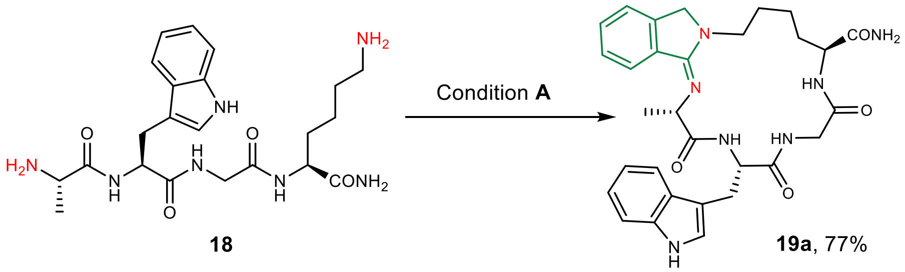

Cyclic peptide **19a** (HCOOH salt, 0.038 mmol, 23.2 mg) was prepared as a single isomer in 77% yield from the linear precursor **18** (TFA salt, 0.05 mmol, 32.7 mg) under the condition **A**.

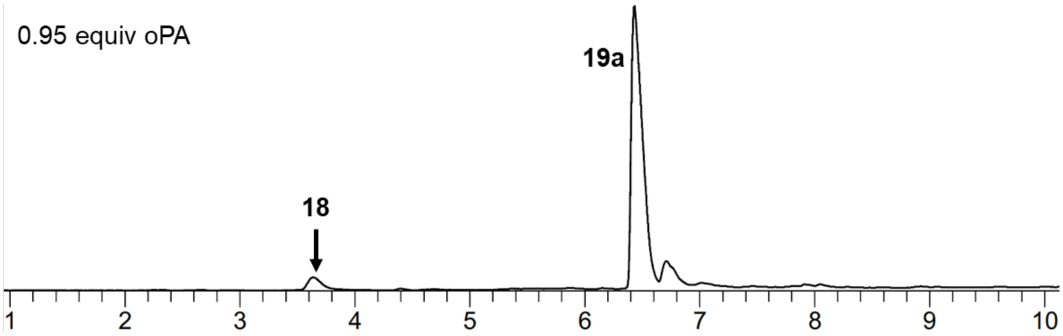

Supplementary Fig. 7. LC trace of the control experiment with 0.95 equiv of OPA.
(A small amounts of unconsumed peptide substrates **18** can be detected by LC-MS analysis)

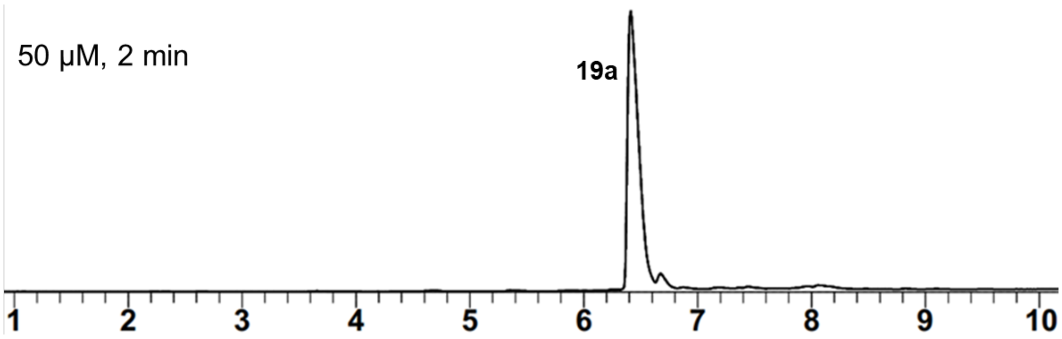

Supplementary Fig. 8. LC trace of the reaction of **18** at 50 µM for 2 min.

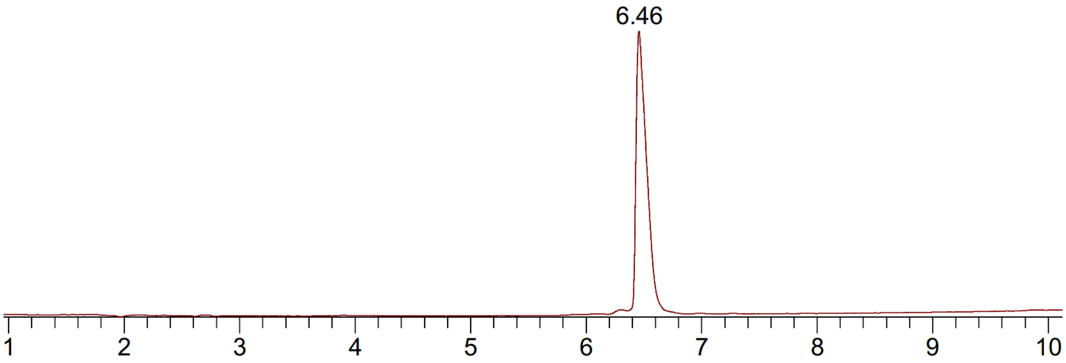

Supplementary Fig. 9. LC trace of purified product **19a** (rt =6.46 min, λ = 280 nm).

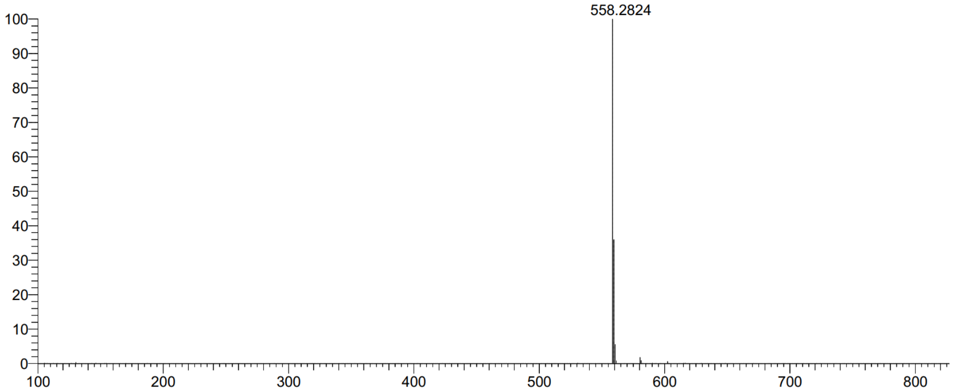

HRMS (ESI): calcd. for $C_{30}H_{36}N_7O_4$ $[M+H]^+$= 558.2823; found 558.2824.

^{1}H NMR (400 MHz, Acetic Acid-*d*$_4$) δ 7.76 – 7.67 (m, 2H), 7.57 (d, *J* = 7.8 Hz, 1H), 7.48 (s, 1H), 7.40 (d, *J* = 8.0 Hz, 1H), 7.23 (s, 1H), 7.14 (t, *J* = 7.6 Hz, 1H), 7.05 (t, *J* = 7.6 Hz, 1H), 4.97 (d, *J* = 19.6 Hz, 1H), 4.77 (d, *J* = 20.4 Hz, 1H), 4.52 (q, *J* = 8.2, 7.2 Hz, 2H), 4.11 (d, *J* = 17.0 Hz, 2H), 3.75 (m, 2H), 3.35 (d, *J* = 7.4 Hz, 2H), 1.85 (d, *J* = 22.8 Hz, 3H), 1.72 (d, *J* = 6.6 Hz, 3H), 1.58–1.48 (m, 2H), 1.30 (d, *J* = 5.6 Hz, 3H).

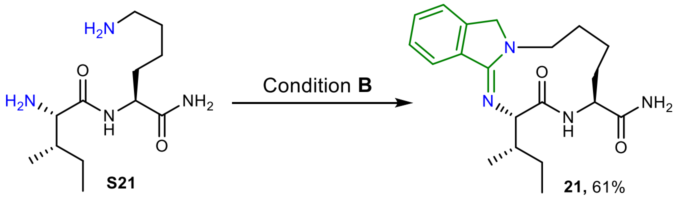

Cyclic peptide **21** (HCOOH salt, 0.012mmol, 5.0 mg) was prepared in 61% yield from the linear precursor **S21** (TFA salt, 0.02 mmol, 9.1 mg) under the condition **B**.

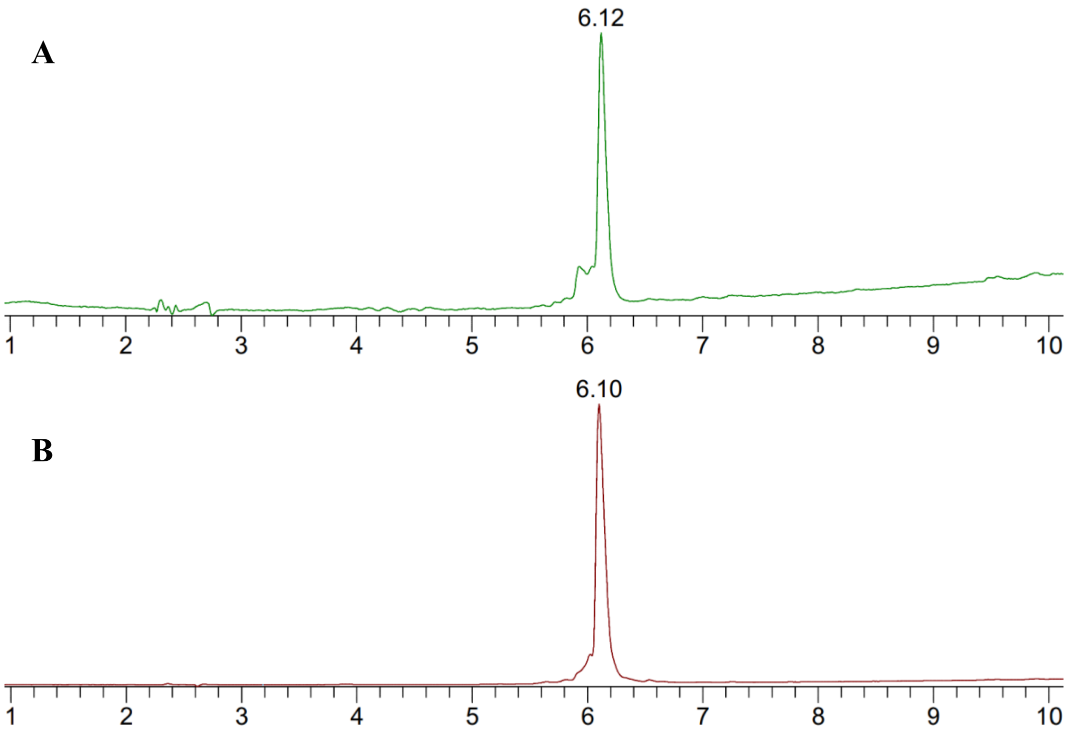

Supplementary Fig. 10. A) Crude UPLC trace of cyclization reaction. **B)** LC trace of product **21** (rt =6.10 min, λ = 254 nm).

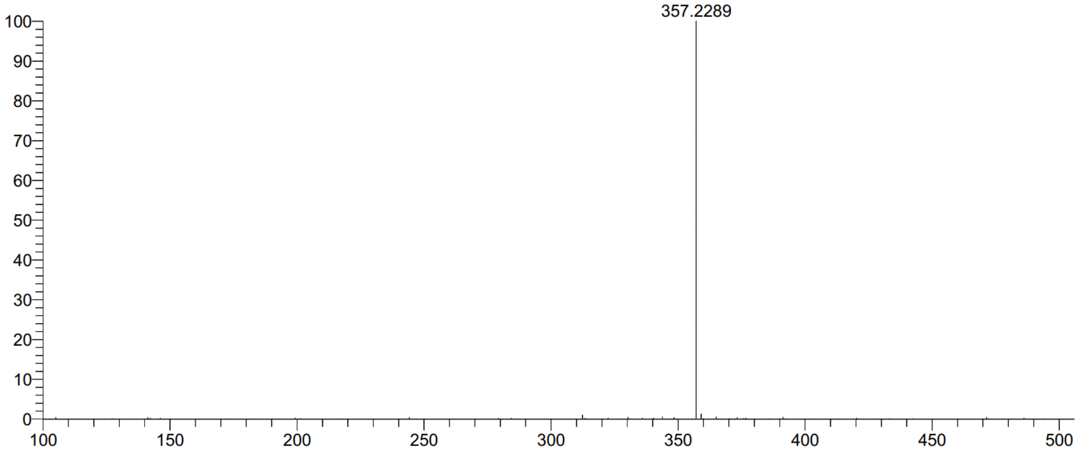

HRMS (ESI): calcd. for $C_{20}H_{29}N_4O_2$ $[M+H]^+$= 357.2285; found 357.2289.

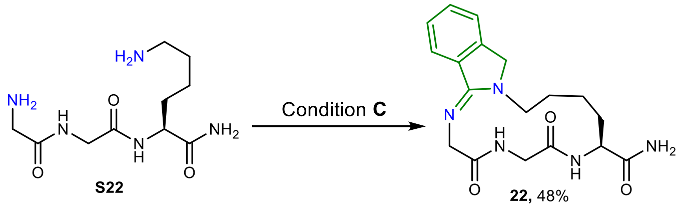

Cyclic peptide **22** (HCOOH salt, 0.010 mmol, 4.1 mg) was prepared as a single isomer in 48% yield from the linear precursor **S22** (TFA salt, 0.02 mmol, 9.1 mg) under the condition **C**.

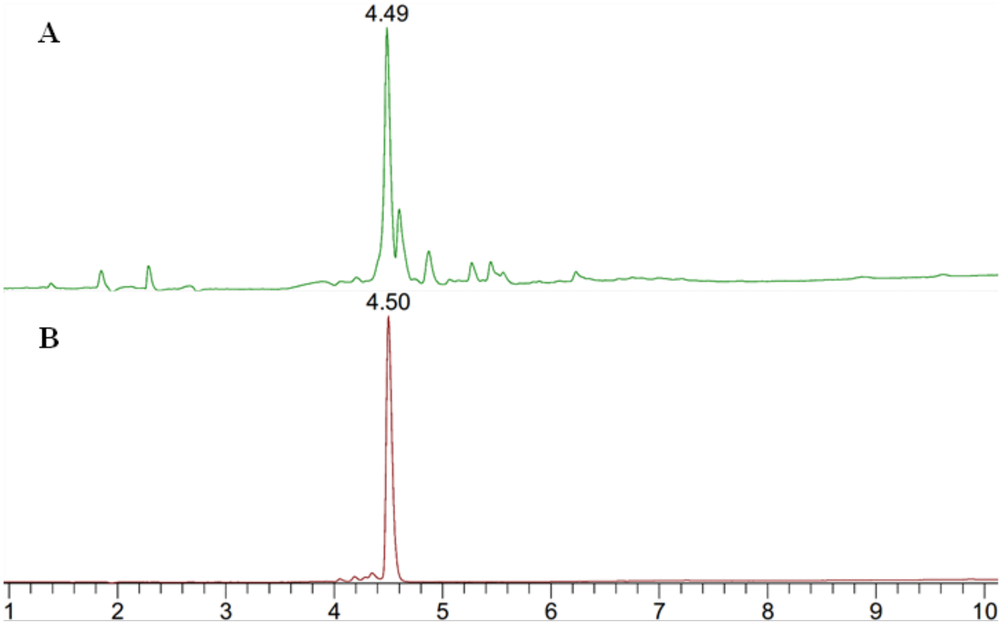

Supplementary Fig. 11. A) Crude UPLC trace of cyclization reaction. **B)** LC trace of purified **22** (rt =4.50 min, λ = 254 nm).

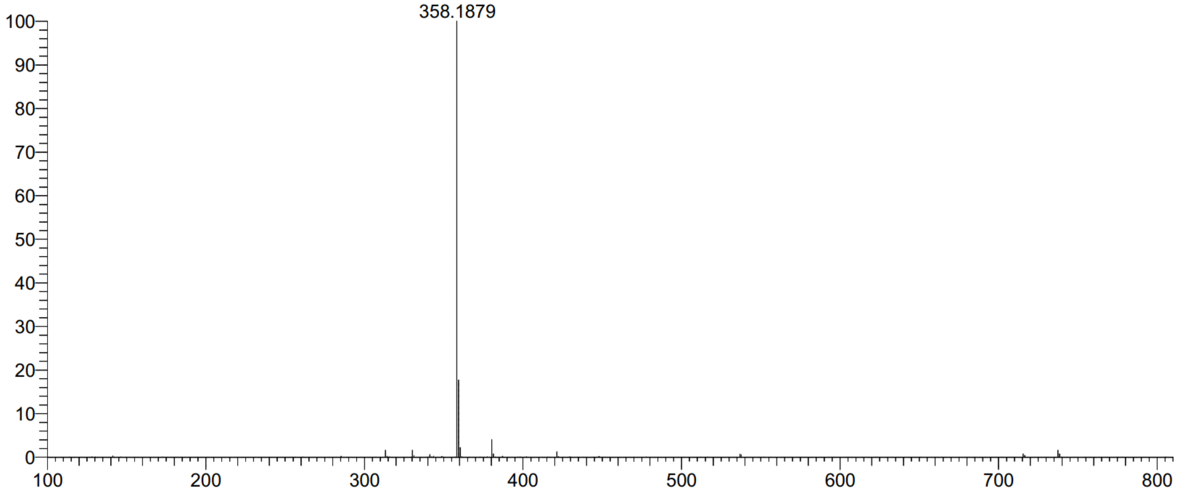

HRMS (ESI): calcd. for $C_{18}H_{24}N_5O_3$ $[M+H]^+$= 358.1874; found 358.1879.

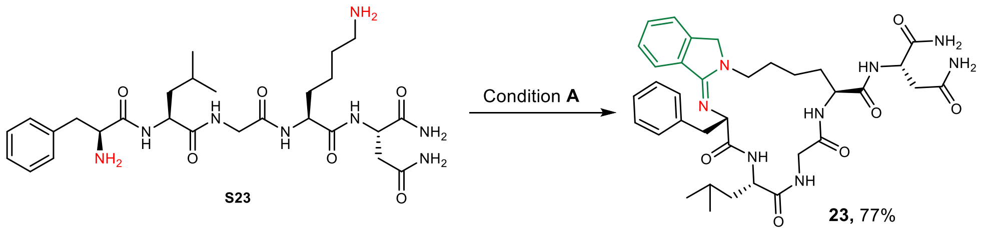

Cyclic peptide **23** (HCOOH salt, 0.015 mmol, 8.6 mg) was prepared as a single isomer in 77% yield from the linear precursor **S23** (TFA salt, 0.02 mmol, 12.5 mg) under the condition **A**.

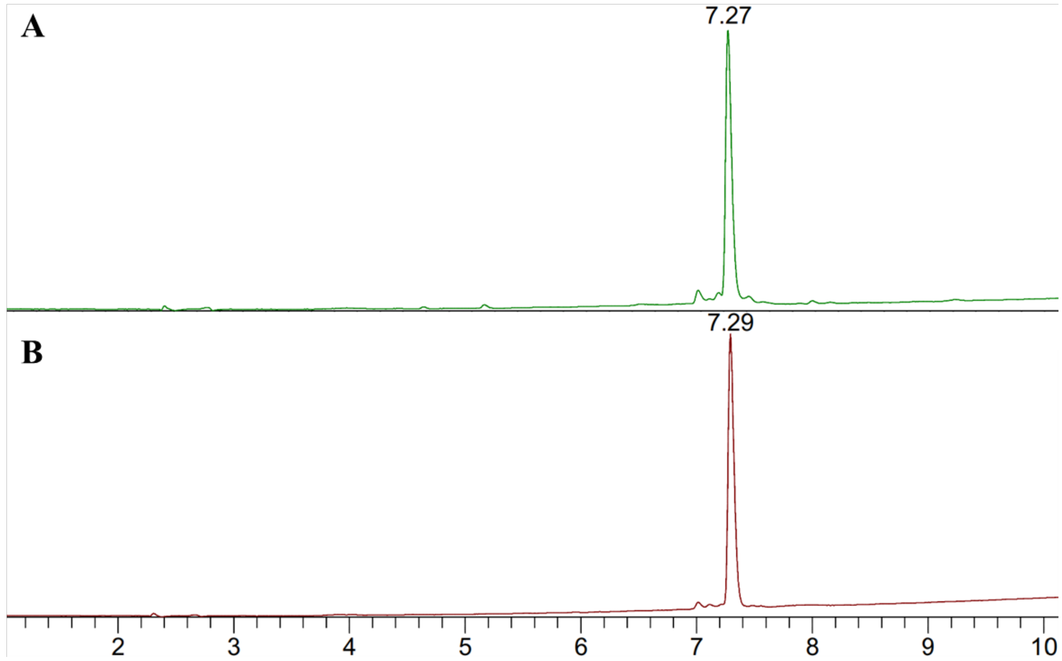

Supplementary Fig. 12. A) Crude UPLC trace of cyclization reaction. **B)** LC trace of purified **23** (rt =5.32 min, λ = 254 nm).

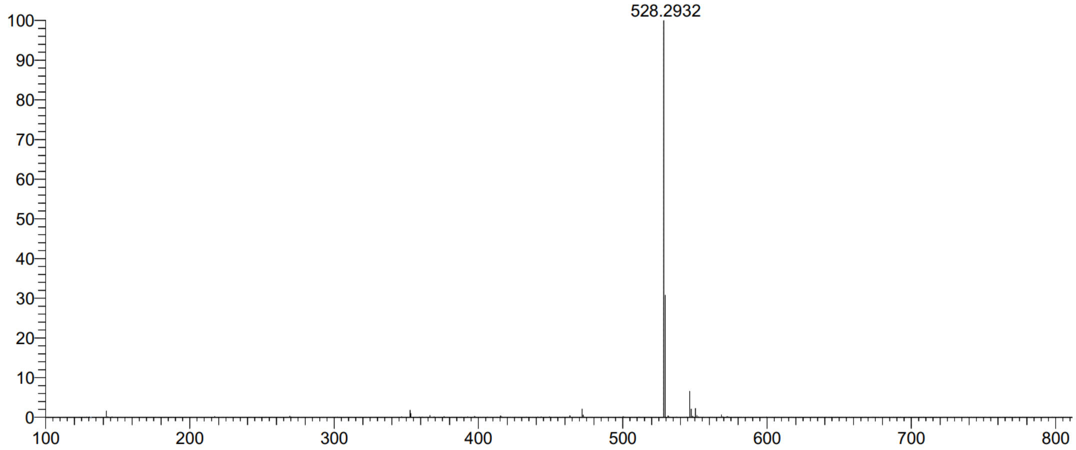

HRMS (ESI): calcd. for $C_{26}H_{38}N_7O_5$ $[M+H]^+$= 528.2929; found 528.2932.

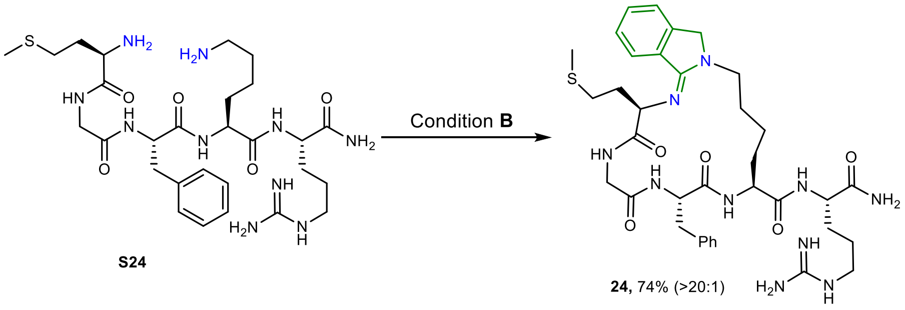

Cyclic peptide **24** (HCOOH salt, 0.015 mmol, 12.4 mg) was prepared as a single isomer in 74% yield from the linear precursor **S24** (TFA salt, 0.02 mmol, 18.6 mg) under the condition **B**.

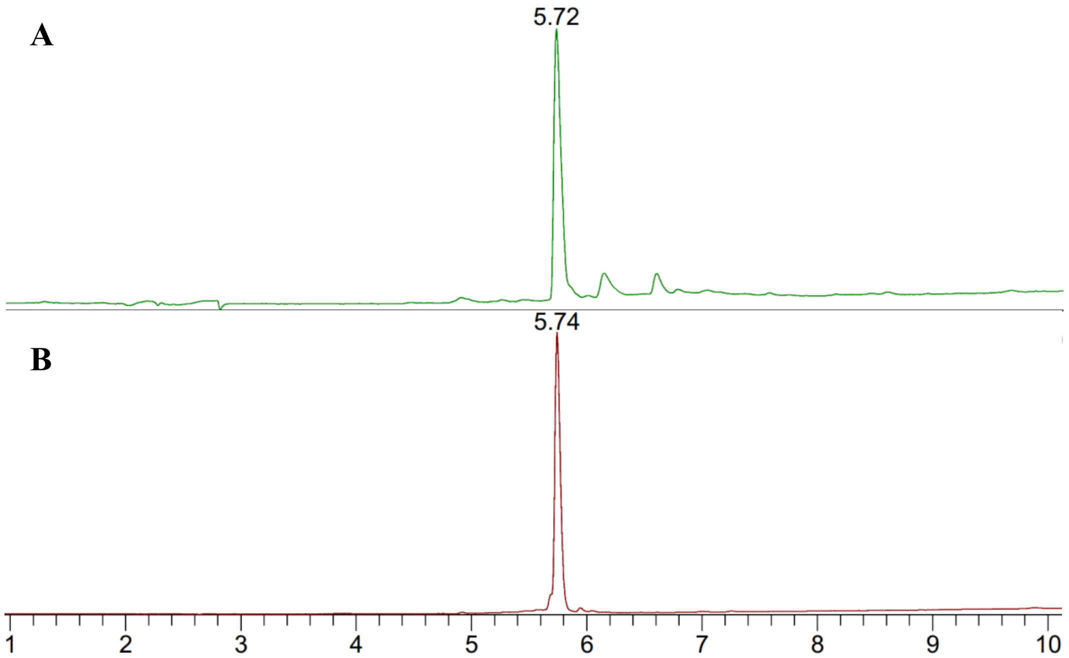

Supplementary Fig. 13. A) Crude UPLC trace of cyclization reaction. **B)** LC trace of purified **24** (rt =5.74 min, λ = 254 nm).

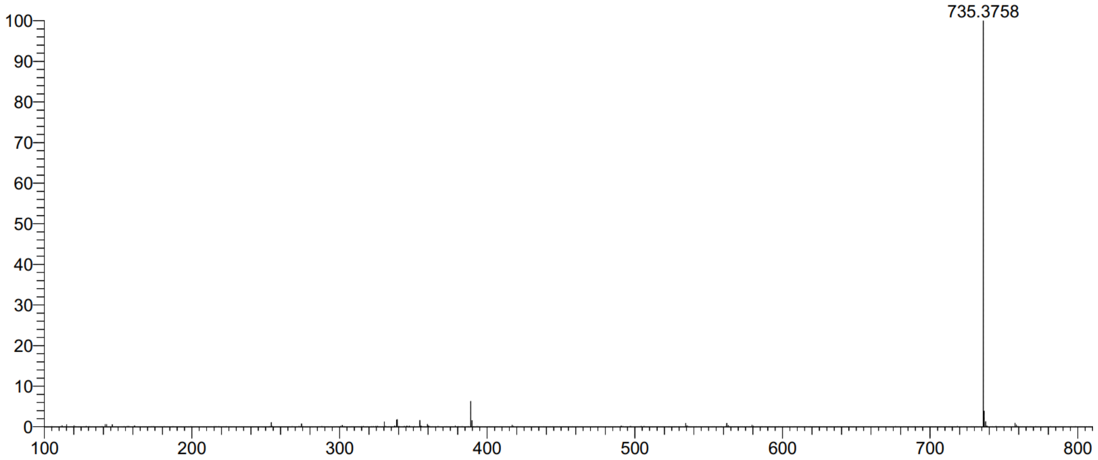

HRMS (ESI): calcd. for $C_{36}H_{51}N_{10}O_5S$ $[M+H]^+$= 735.3759; found 735.3758.

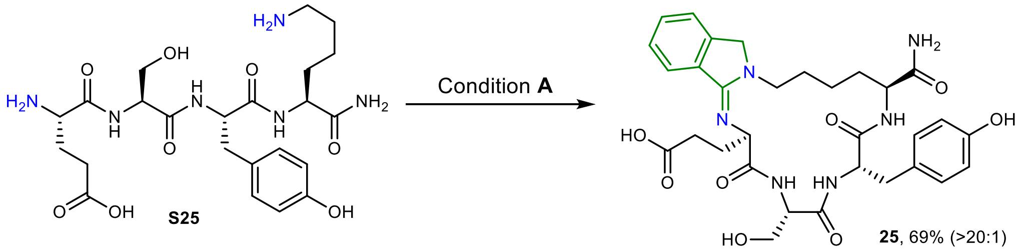

Cyclic peptide **25** (HCOOH salt, 0.014 mmol, 9.5 mg) was prepared as a single isomer in 69% yield from the linear precursor **S25** (TFA salt, 0.02 mmol, 14.4 mg) under the condition **A**.

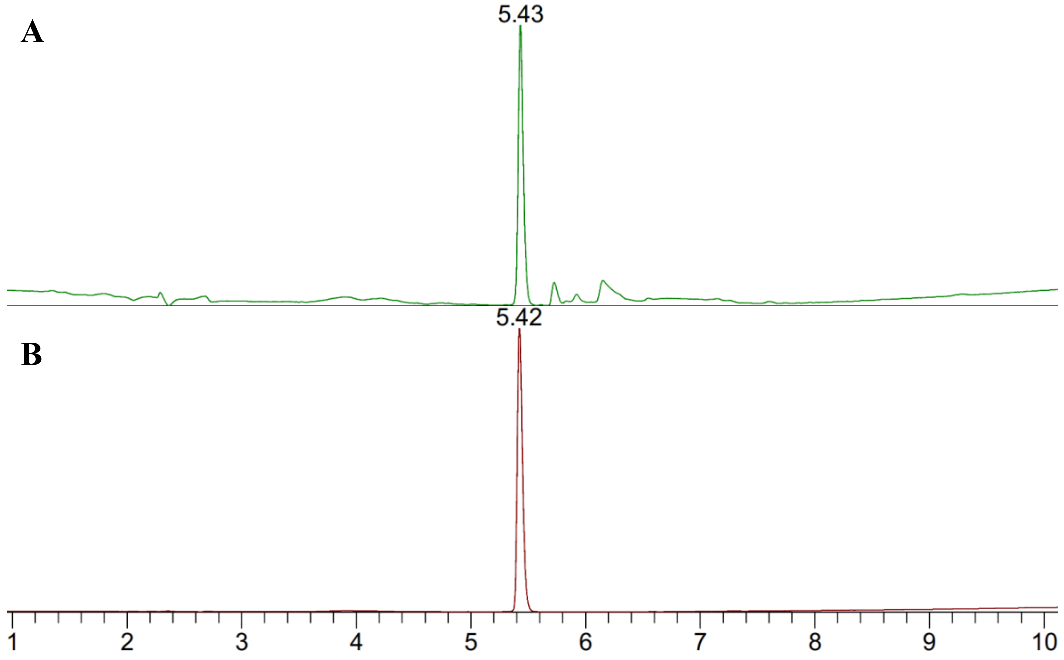

Supplementary Fig. 14. A) Crude UPLC trace of cyclization reaction. **B)** LC trace of purified **24** (rt =5.42 min, λ = 254 nm).

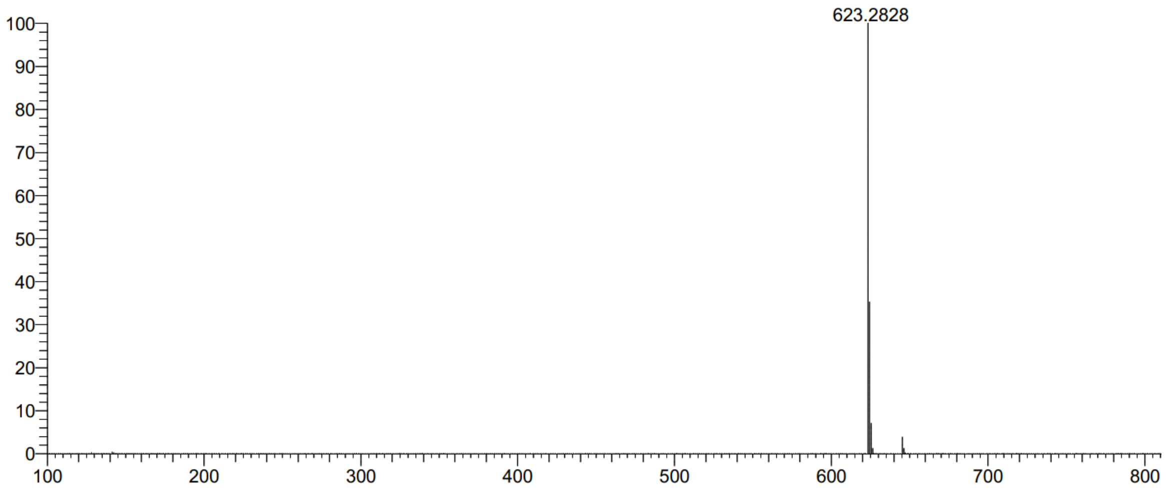

HRMS (ESI): calcd. for $C_{31}H_{39}N_6O_8$ $[M+H]^+$= 623.2824; found 628.2828.

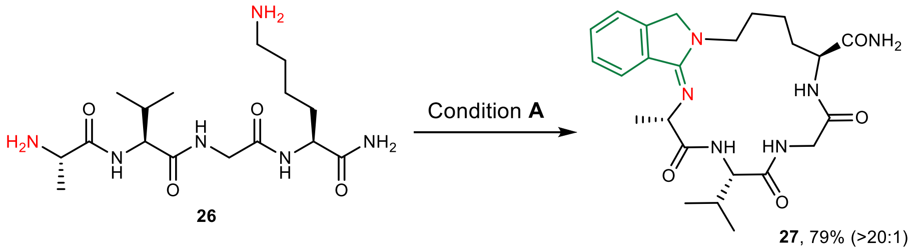

Cyclic peptide **27** (HCOOH salt, 0.041 mmol, 21.2 mg) was prepared in 79% yield from the linear precursor **26** (TFA salt, 0.05 mmol, 28.3 mg) under the condition **A**.

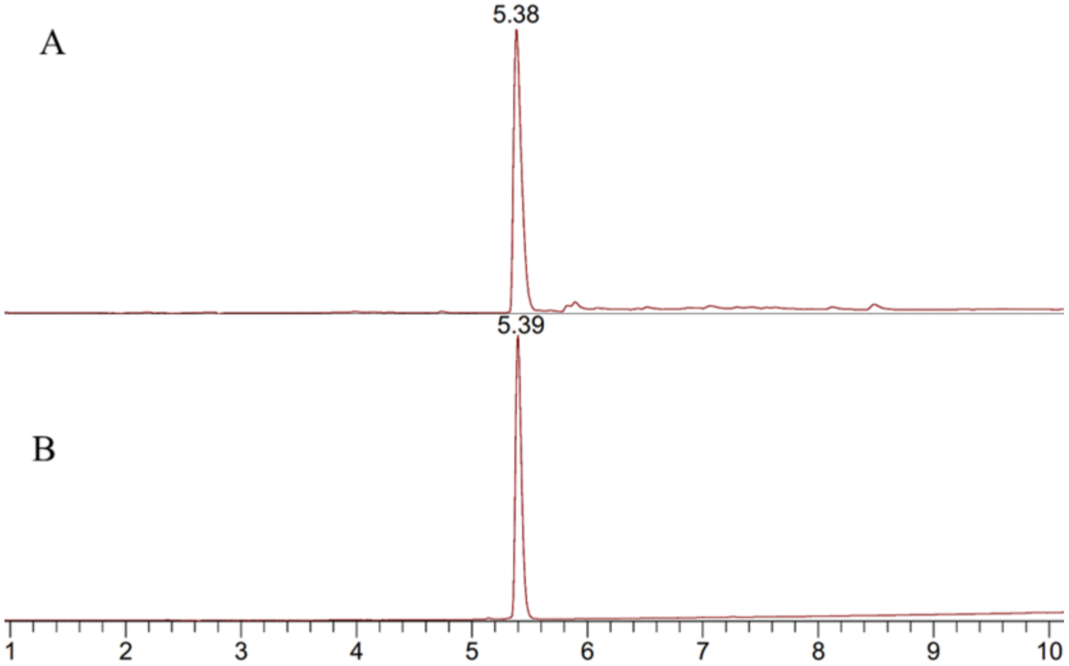

Supplementary Fig. 15. A) Crude UPLC trace of cyclization reaction. **B)** LC trace of purified **25** (rt =5.39 min, λ = 254 nm).

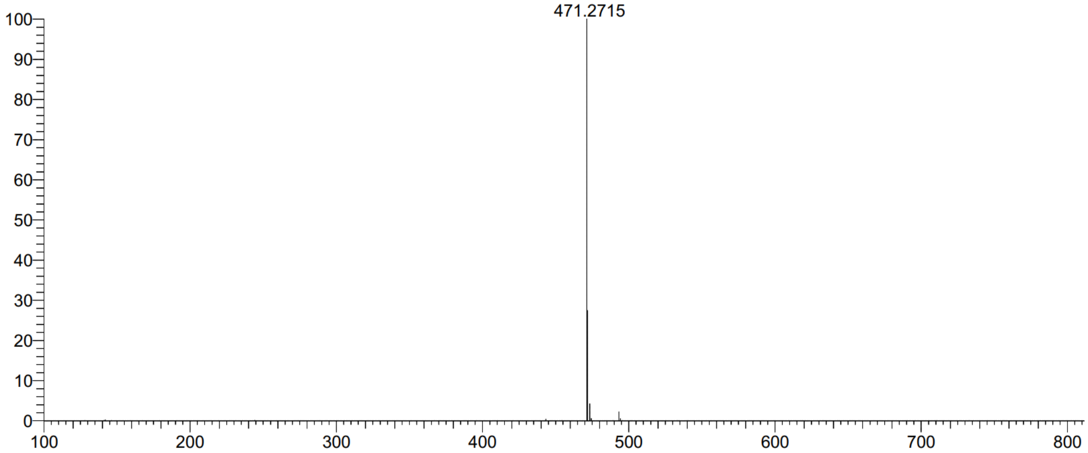

HRMS (ESI): calcd. for $C_{24}H_{35}N_6O_4$ $[M+H]^+$= 471.2714; found 471.2715.

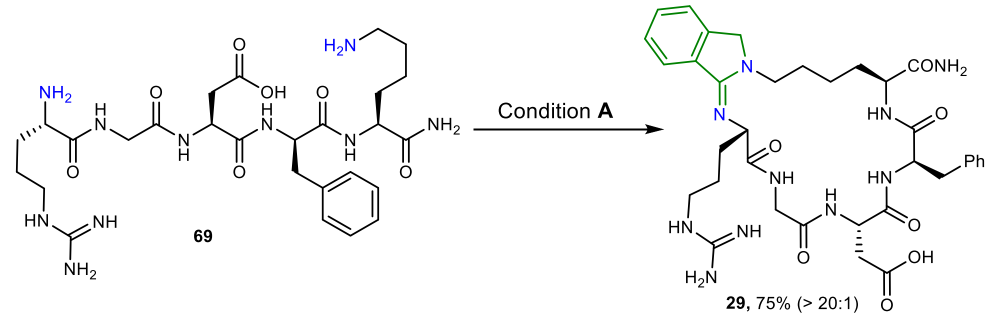

Cyclic peptide **29** (HCOOH salt, 0.015 mmol, 11.5 mg) was prepared in 75% yield from the linear precursor **69** (TFA salt, 0.02 mmol, 18.3 mg) under the condition **A**.

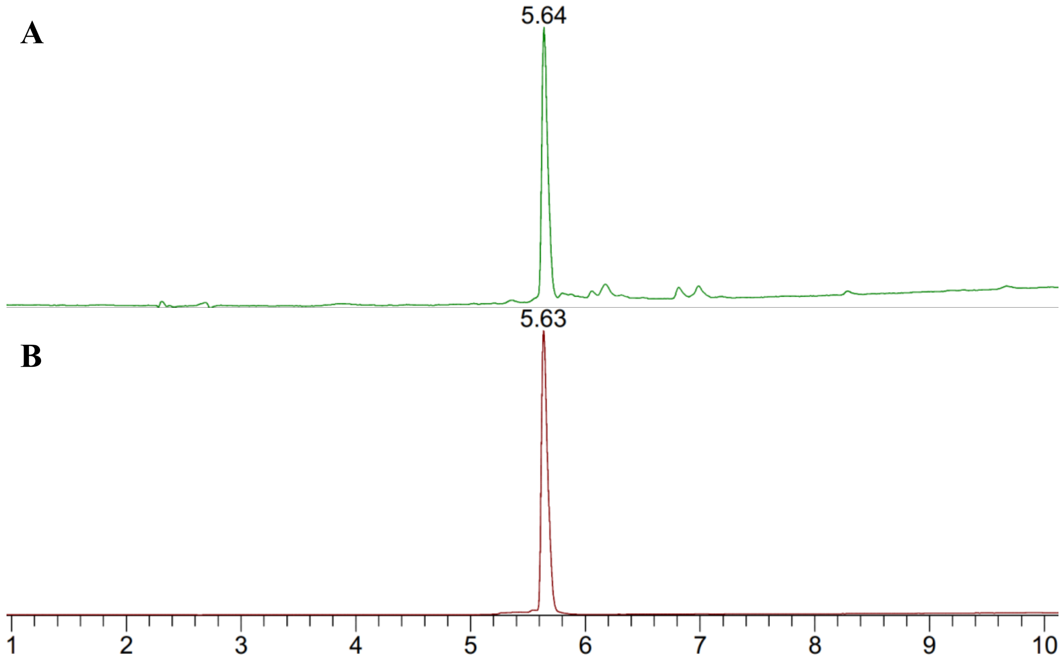

Supplementary Fig. 16. A) Crude UPLC trace of cyclization reaction. **B)** LC trace of purified **28** (rt =5.63 min, λ = 254 nm).

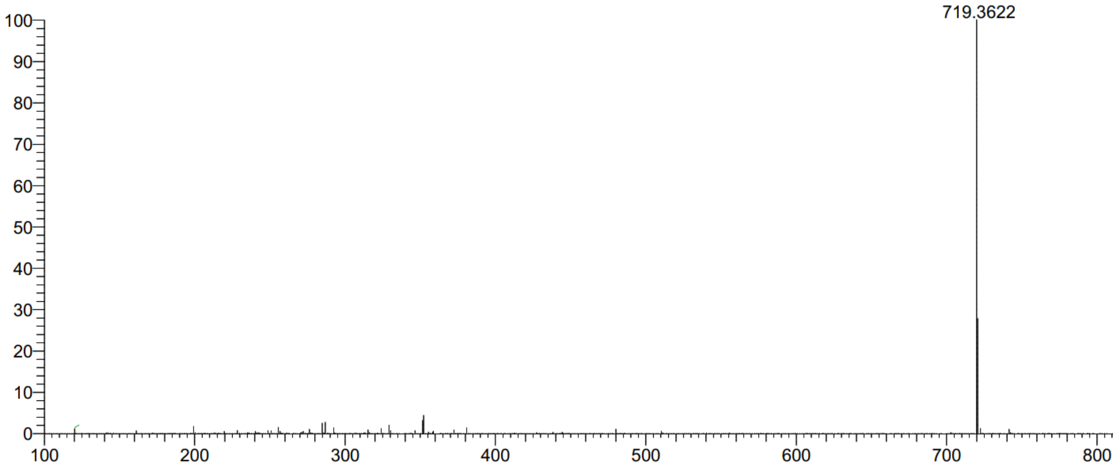

HRMS (ESI): calcd. for $C_{35}H_{47}N_{10}O_7$ $[M+H]^+$= 719.3624; found 719.3622.

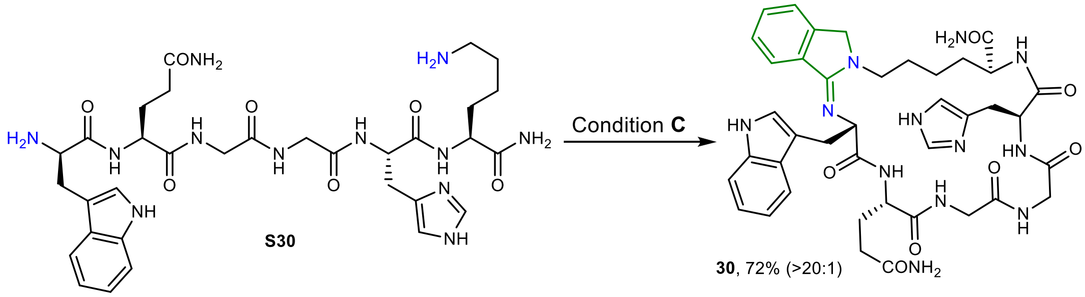

Cyclic peptide **30** (HCOOH salt, 0.014 mmol, 13.0 mg) was prepared in 72% yield from the linear precursor **S30** (TFA salt, 0.02 mmol, 20.0 mg) under the condition **C**.

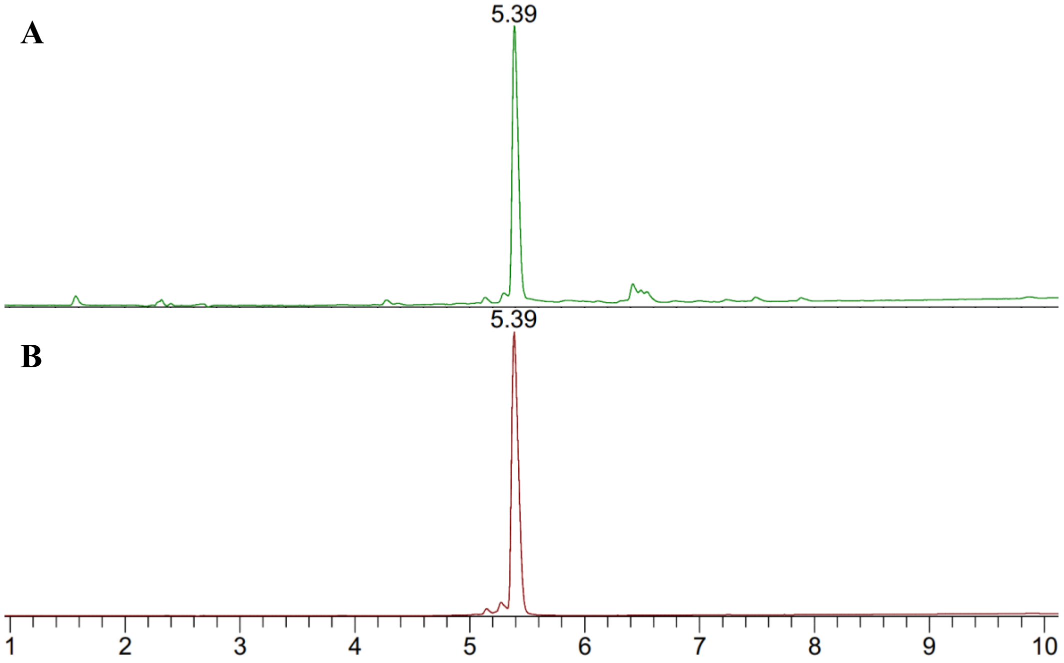

Supplementary Fig. 17. A) Crude UPLC trace of cyclization reaction. **B)** LC trace of purified **29** (rt =5.39 min, λ = 254 nm).

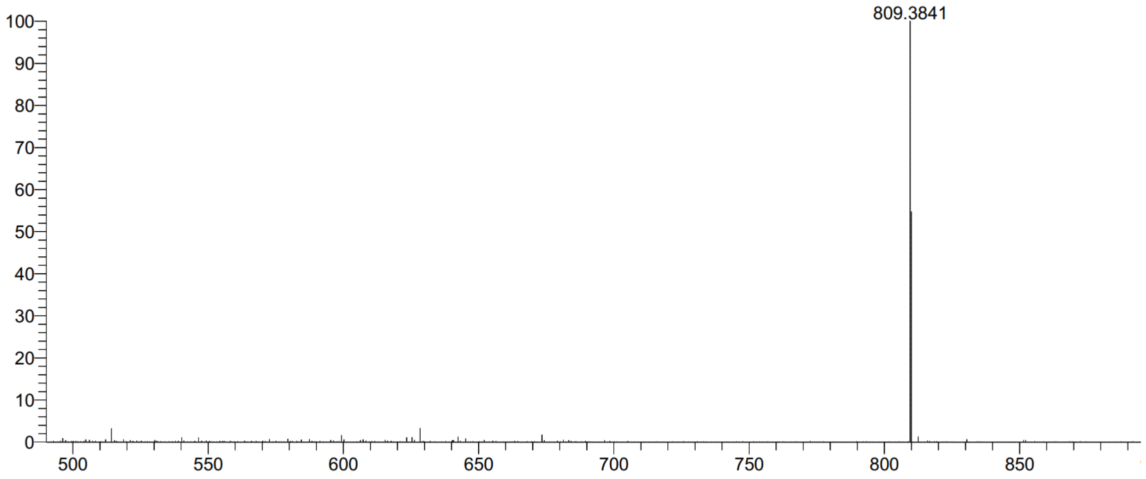

HRMS (ESI): calcd. for $C_{40}H_{49}N_{12}O_7$ $[M+H]^+$= 809.3842; found 809.3841.

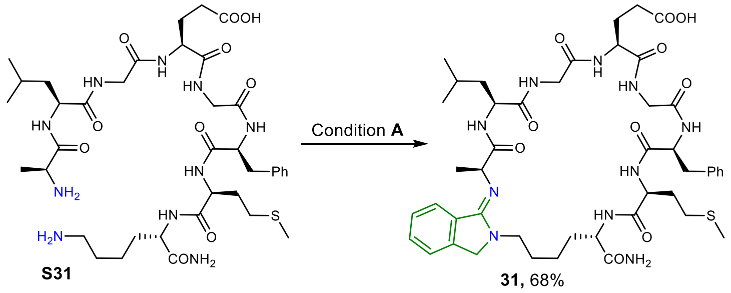

Cyclic peptide **31** (HCOOH salt, 0.0068 mmol, 6.7 mg) was prepared in 68% yield from the linear precursor **S31** (TFA salt, 0.01 mmol, 10.5 mg) under the condition **A**.

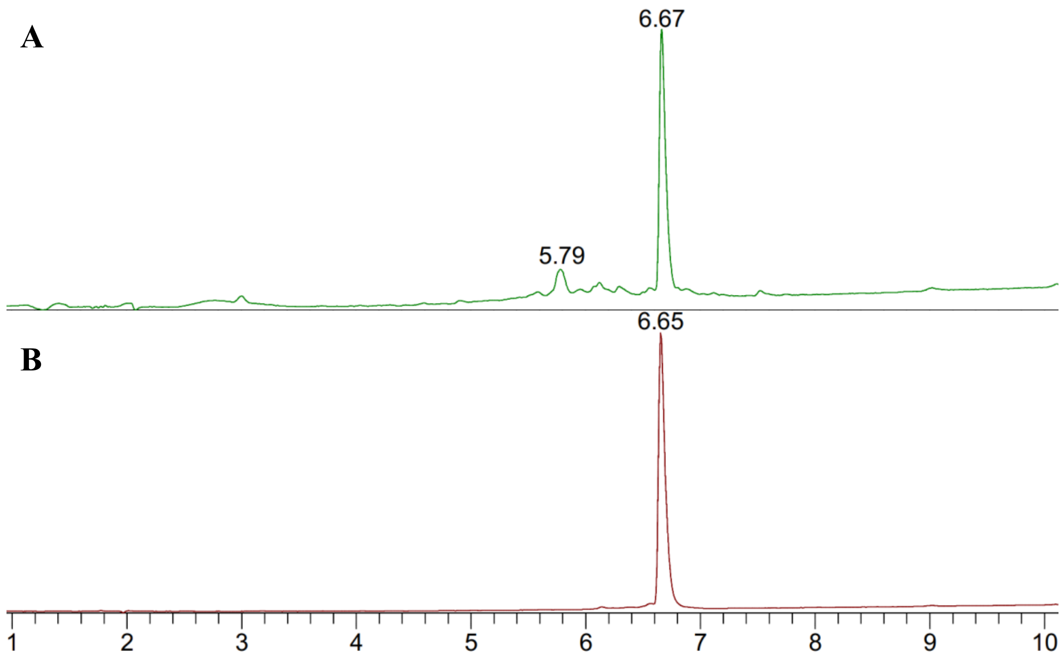

Supplementary Fig. 18. A) Crude UPLC trace of reaction. **B)** LC trace of purified product **31** (rt =6.65 min, λ = 254 nm).

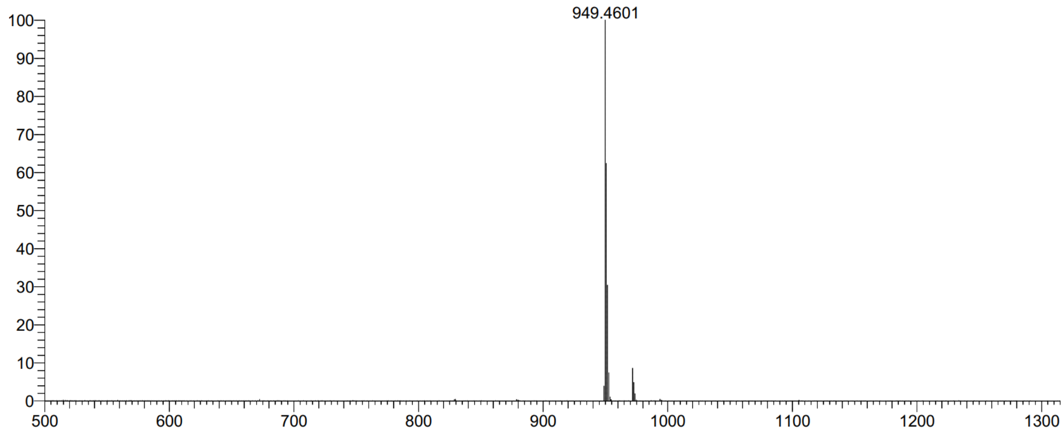

HRMS (ESI): calcd. for $C_{46}H_{65}N_{10}O_{10}S$ $[M+H]^+$= 949.4600; found 949.4601.

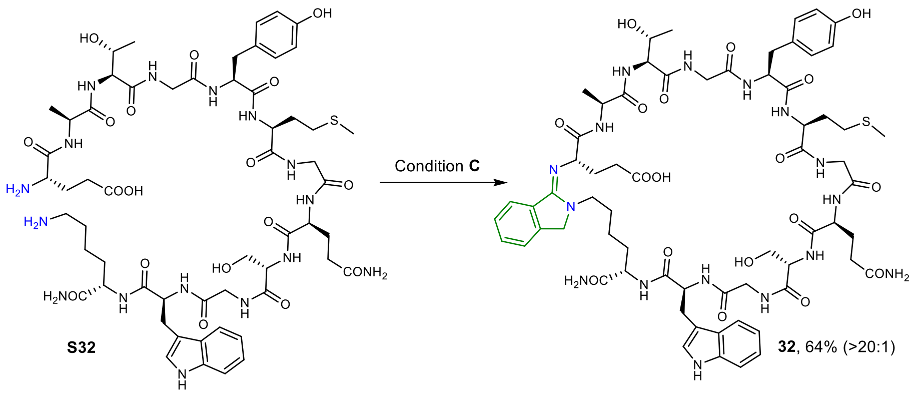

Cyclic peptide **32** (HCOOH salt, 0.0064 mmol, 9.3 mg) was prepared in 64% yield from the linear precursor **S32** (TFA salt, 0.01 mmol, 15.1 mg) under the condition **C**.

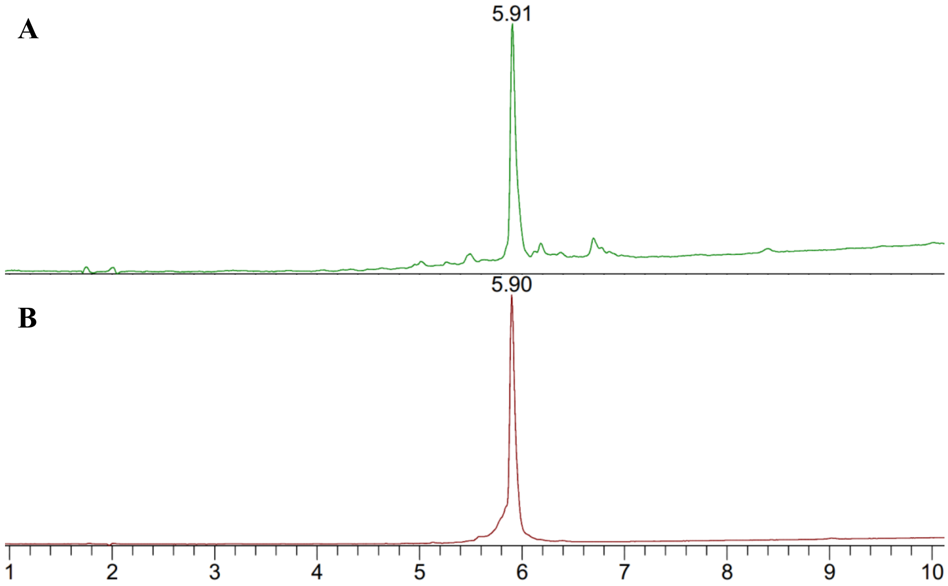

Supplementary Fig. 19. A) Crude UPLC trace of reaction. **B)** LC trace of purified product **32** (rt =5.90 min, λ = 254 nm).

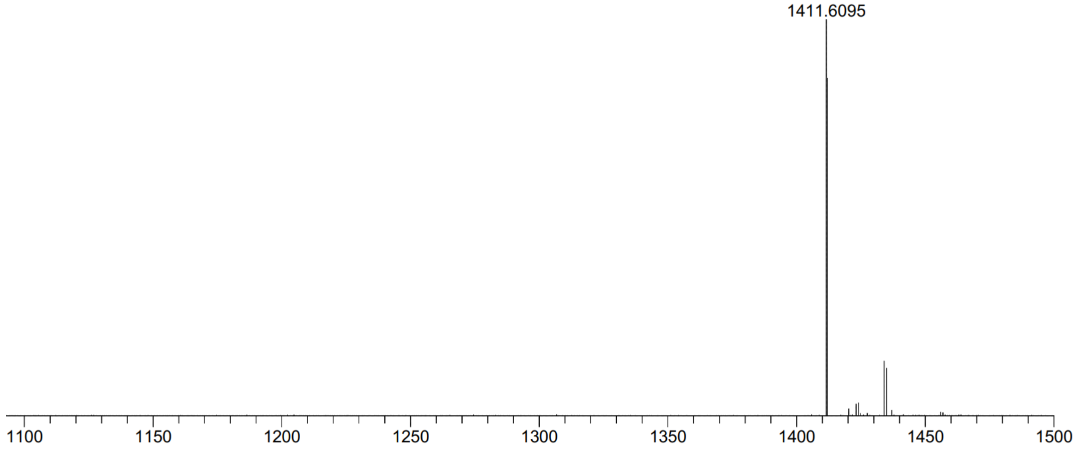

HRMS (ESI): calcd. for $C_{65}H_{87}N_{16}O_{18}S$ $[M+H]^+$= 1411.6099; found 1411.6095.

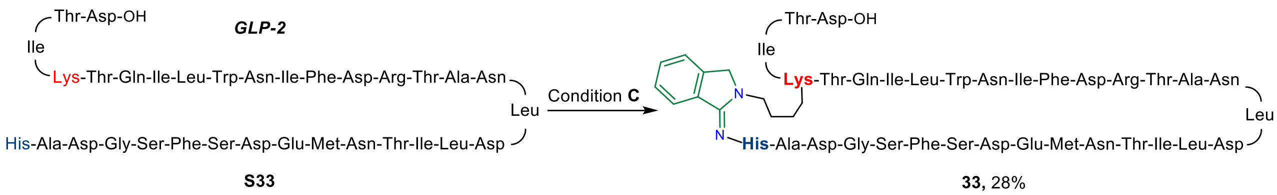

Cyclic peptide **33** (HCOOH salt, 0.00067 mmol, 2.7 mg) was prepared as two isomers in 28% yield from the linear precursor **S33** (TFA salt, 0.0024 mmol, 10.0 mg) under the condition **C** (1.0 mM).

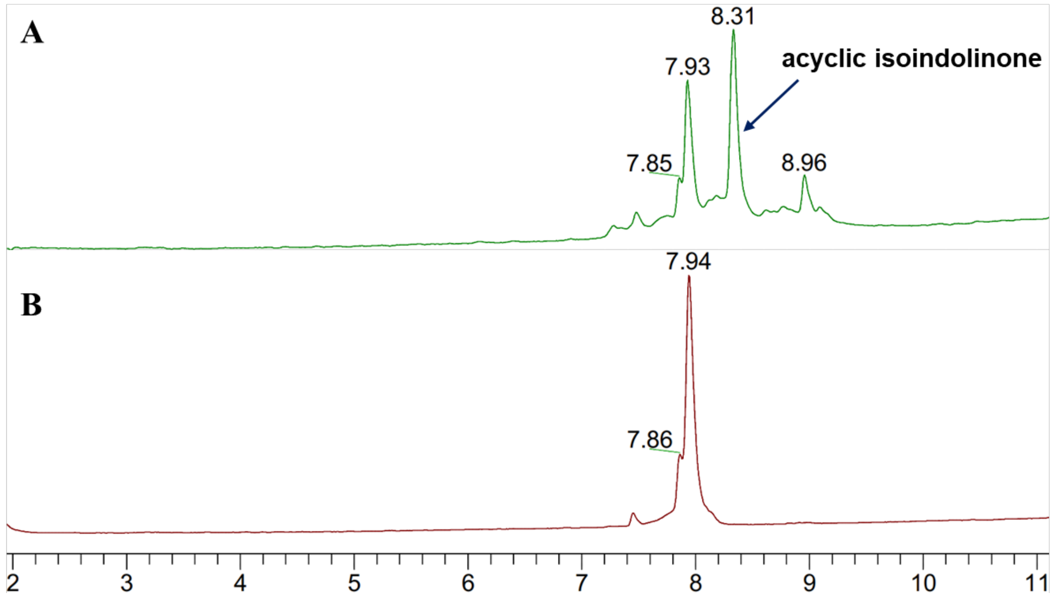

Supplementary Fig. 20. A) Crude UPLC trace of reaction. **B)** LC trace of purified product **33** (rt =7.86 & 7.94 min, λ = 254 nm).

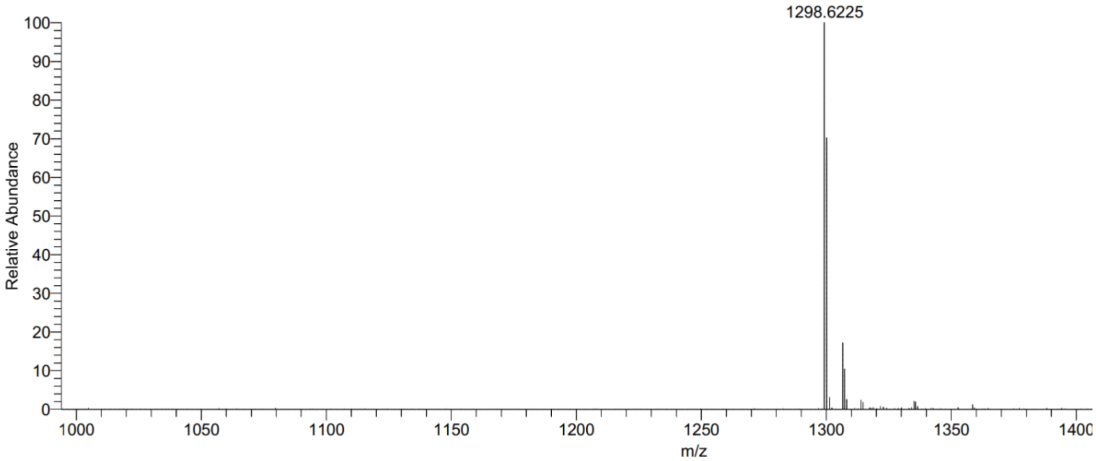

HRMS (ESI): calcd. for $C_{174}H_{261}N_{44}O_{56}S$ $[M+3H]^{3+}$= 1298.6222; found 1298.6225.

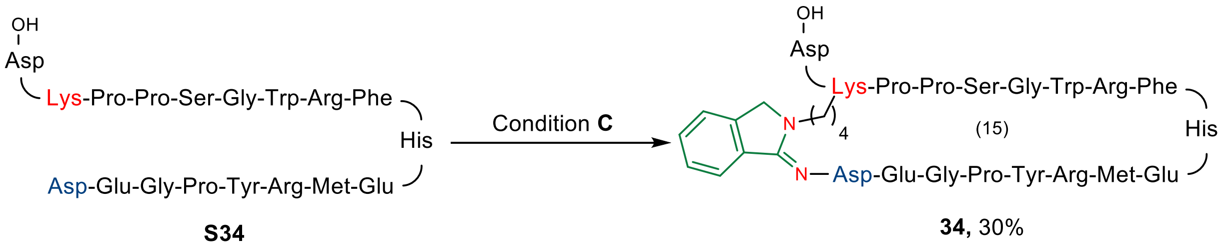

Cyclic peptide **34** (HCOOH salt, 0.0011 mmol, 2.8 mg) was prepared in 30% yield from the linear precursor **S34** (TFA salt, 0.0037 mmol, 10 mg) under the condition **C**.

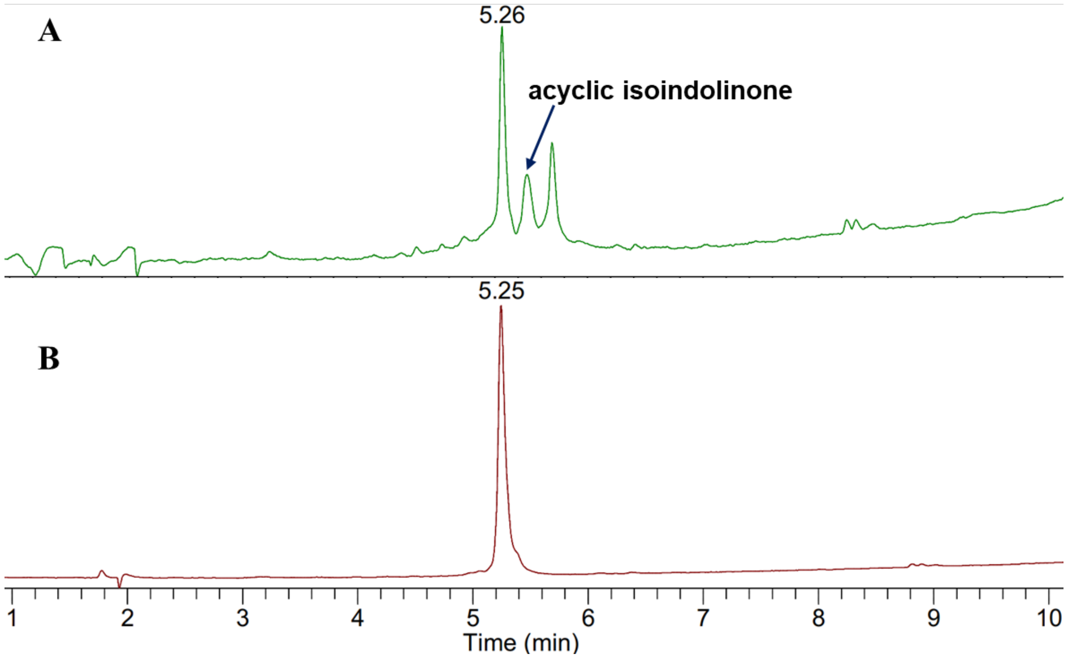

Supplementary Fig. 21. A) Crude UPLC trace of cyclization reaction. **B)** LC trace of purified **34** (rt =5.25 min, λ = 254 nm).

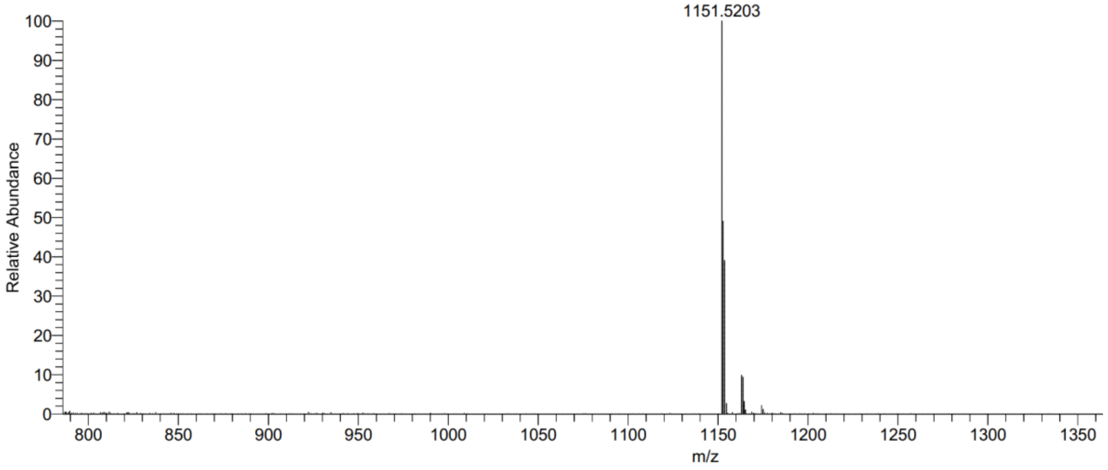

HRMS (ESI): calcd. for $C_{106}H_{143}N_{29}O_{28}S$ $[M+2H]^{2+}$= 1151.5200; found 1151.5203.

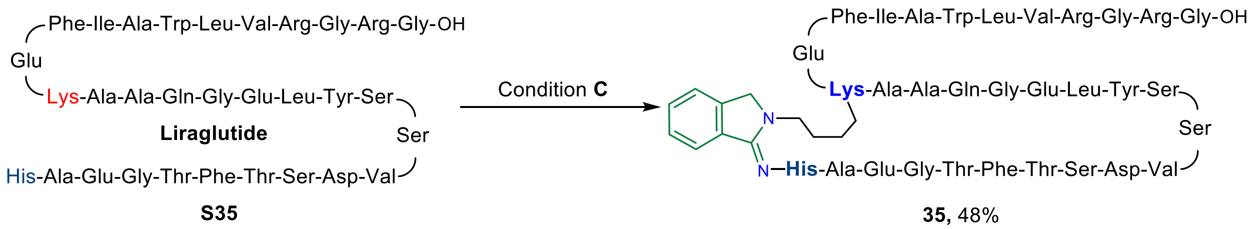

Cyclic peptide **35** (HCOOH salt, 0.0009 mmol, 3.4 mg) was prepared in 48% yield from the linear precursor **S35** (TFA salt, 0.0019 mmol, 7.0 mg) under the condition **C** (1.0 mM).

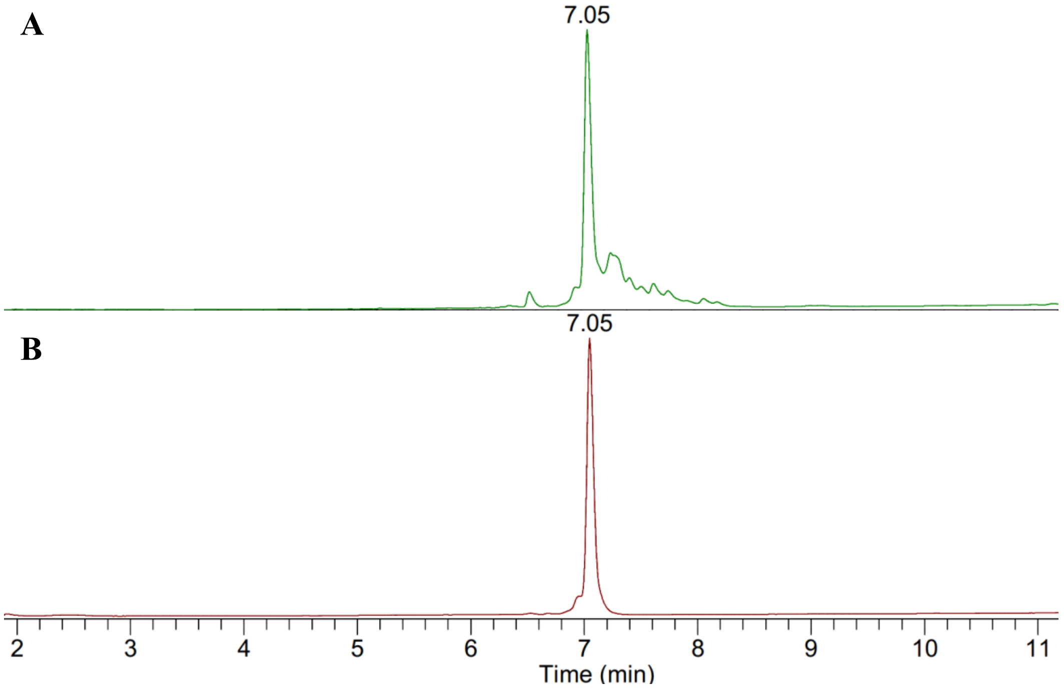

Supplementary Fig. 22. A) Crude UPLC trace of cyclization reaction. **B)** LC trace of purified **35** (rt =7.05 min, λ = 254 nm).

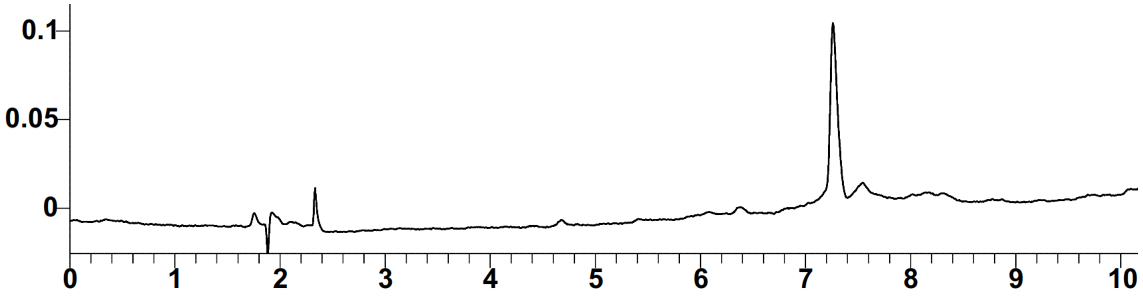

Supplementary Fig. 23. LC trace of the reaction of **S35** in the concentration of 50 μM.

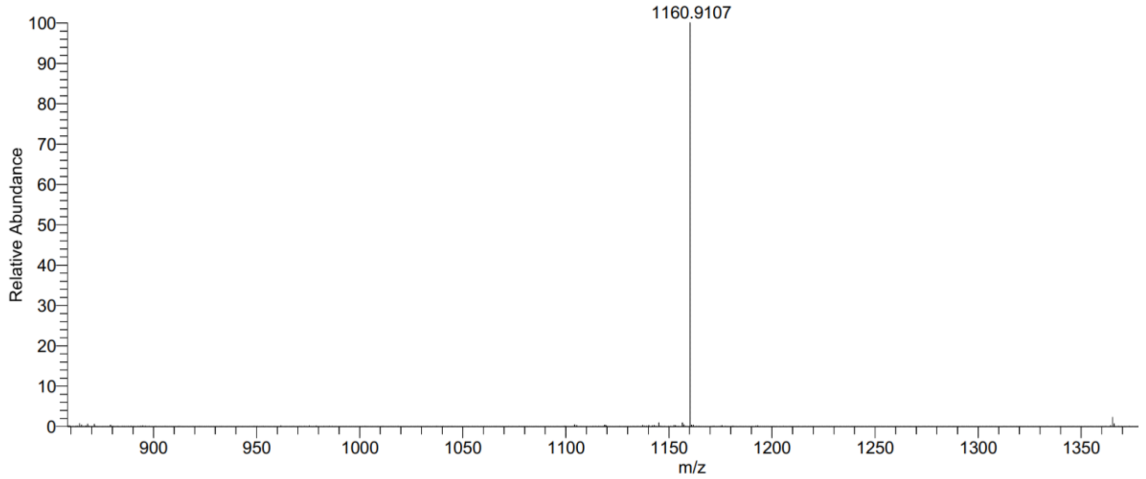

HRMS (ESI): calcd. for $C_{159}H_{234}N_{43}O_{46}$ $[M+3H]^{3+}$= 1160.9103; found 1160.9107.

Cyclization of 17 with OPA analogs

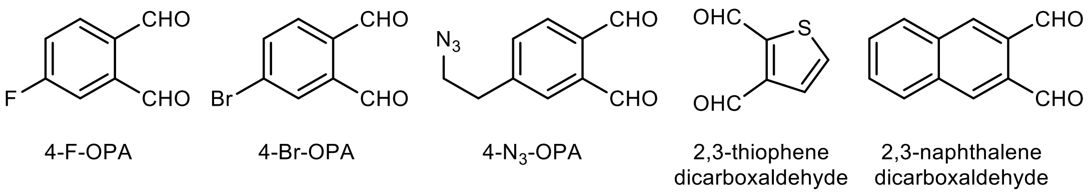

Compounds 4-F-OPA, 4-Br-OPA and 4-N_3-OPA were prepared using the reported procedure[1,2], and the other two OPA analogs were commercially available.

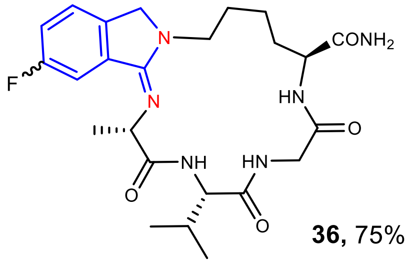

36, 75%

Cyclic peptide **36** (HCOOH salt, 0.015 mmol, 8.0 mg) was prepared in 75% yield from the linear precursor **25** (TFA salt, 0.02 mmol, 11.3 mg) under the condition **C**.

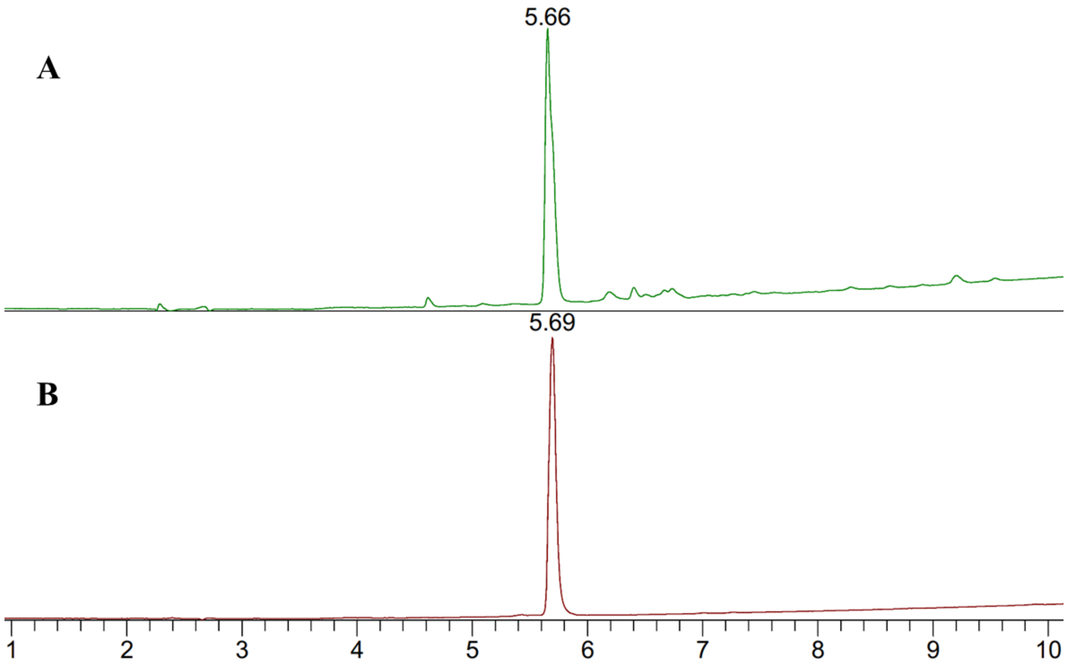

Supplementary Fig. 24. A) Crude UPLC trace of reaction. **B)** LC trace of Purified product **36** (rt = 5.69 min, λ = 254 nm).

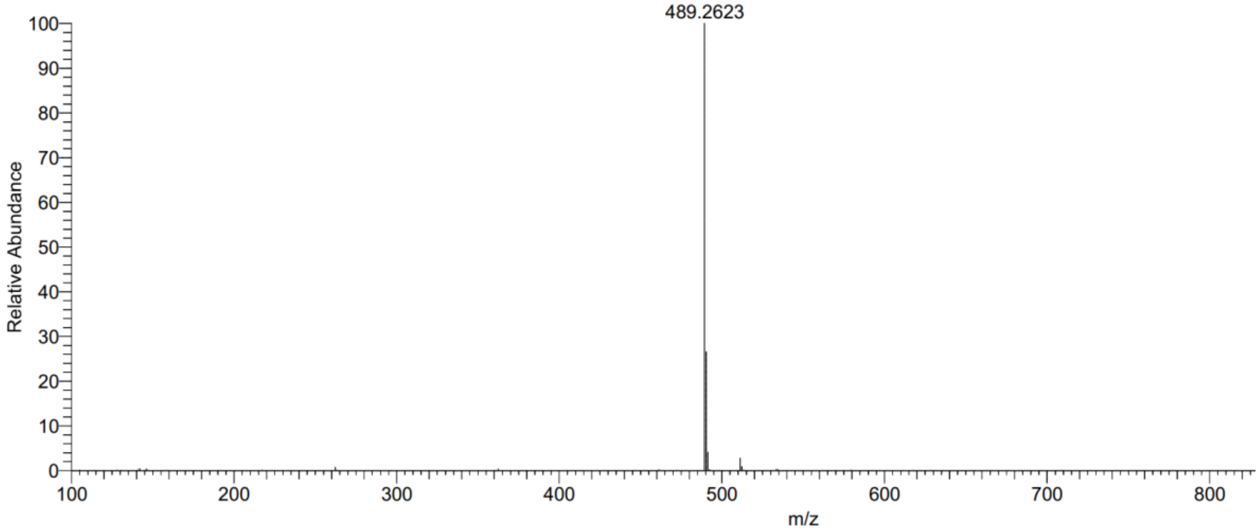

HRMS (ESI): calcd. for $C_{24}H_{34}FN_6O_4$ $[M+H]^+$ = 489.2620; found 489.2623.

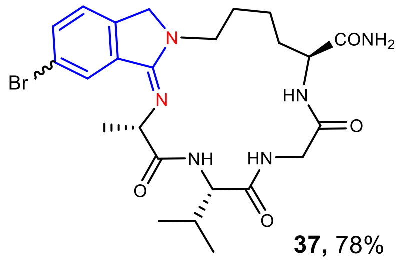

37, 78%

Cyclic peptide **37** (HCOOH salt, 0.015 mmol, 9.1 mg) was prepared in 78% yield from the linear precursor **25** (TFA salt, 0.02 mmol, 11.3 mg) under the condition **C**.

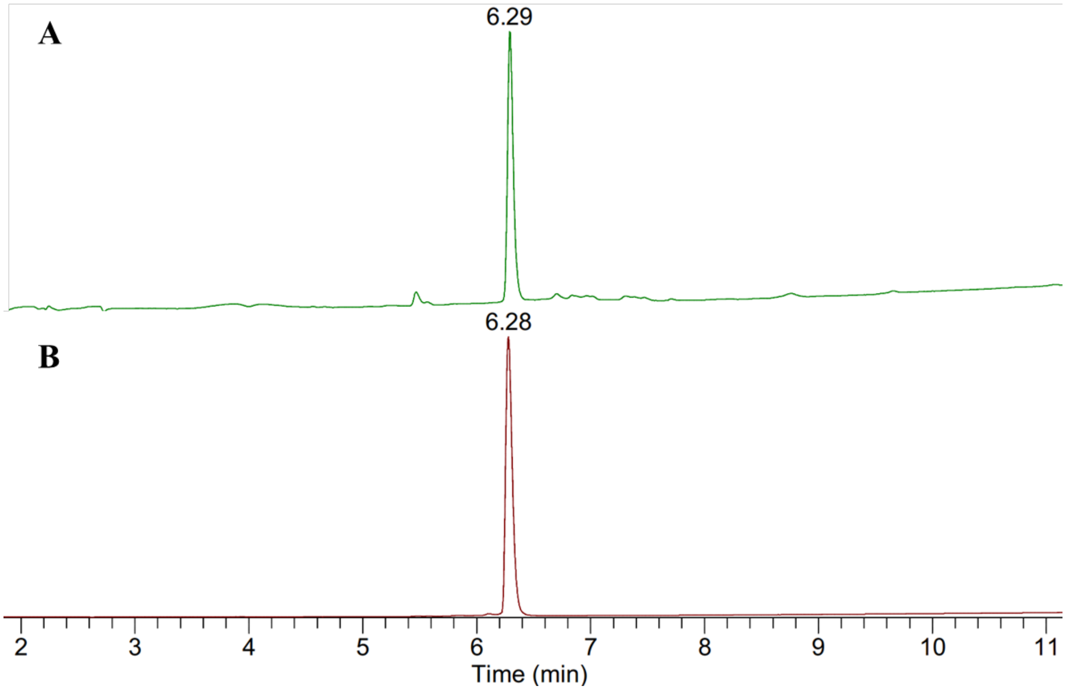

Supplementary Fig. 25. A) Crude UPLC trace of reaction. **B)** LC trace of purified product **37** (rt = 6.28 min, λ = 254 nm).

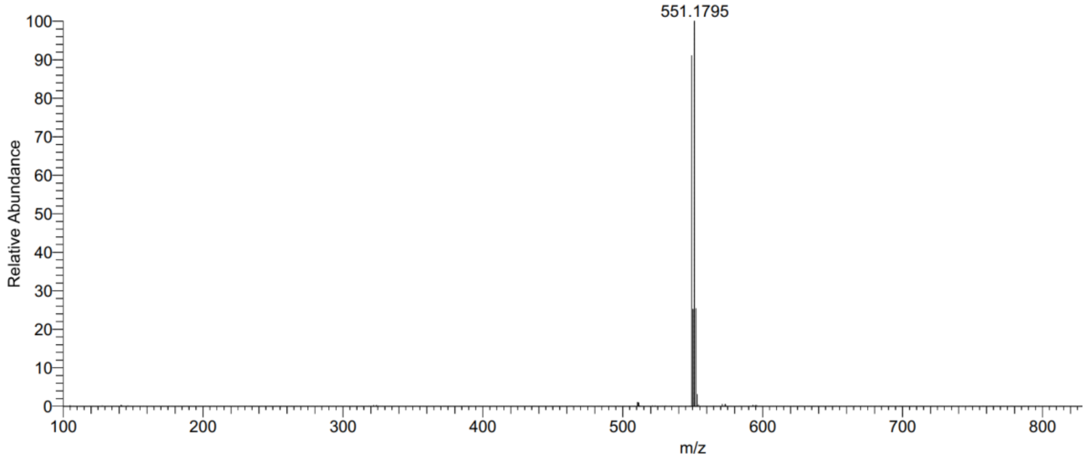

HRMS (ESI): calcd. for $C_{24}H_{34}BrN_6O_4$ $[M+H]^+$= 549.1819; found 551.1795.

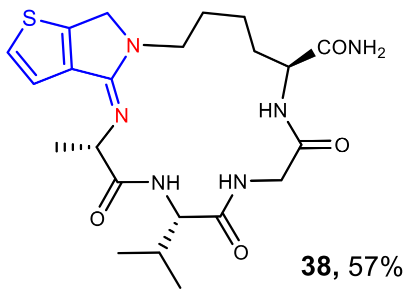

38, 57%

Cyclic peptide **38** (HCOOH salt, 0.011 mmol, 5.9 mg) was prepared in 57% yield from the linear precursor **25** (TFA salt, 0.02 mmol, 11.3 mg) under the condition **C**.

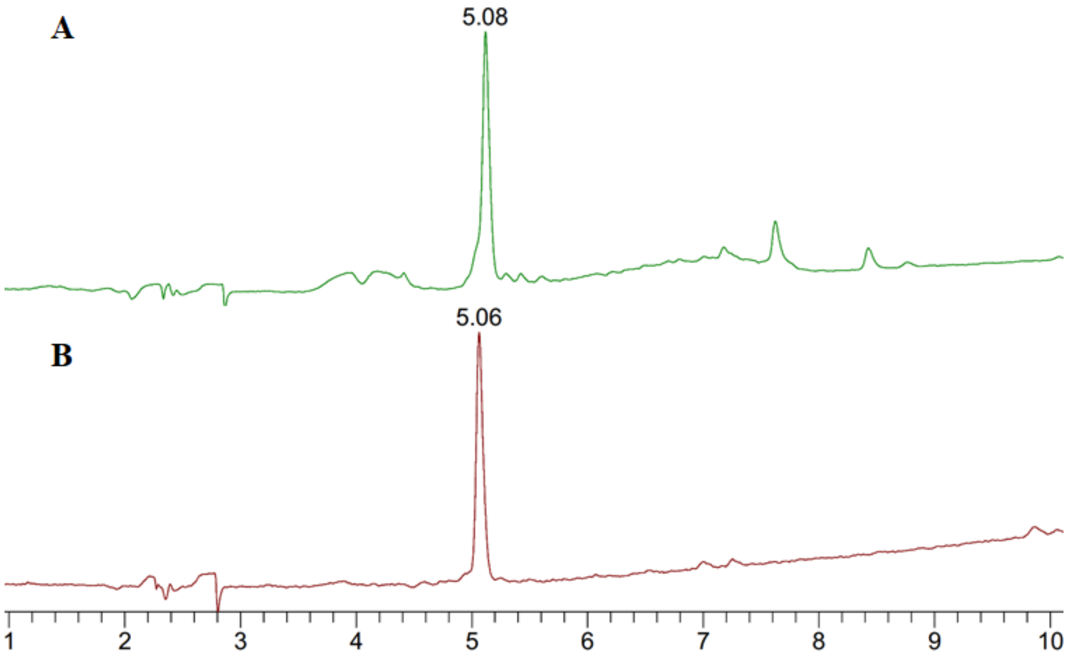

Supplementary Fig. 26. A) Crude UPLC trace of reaction. **B)** UPLC trace of purified product **38** (rt = 5.06 min, λ = 254 nm).

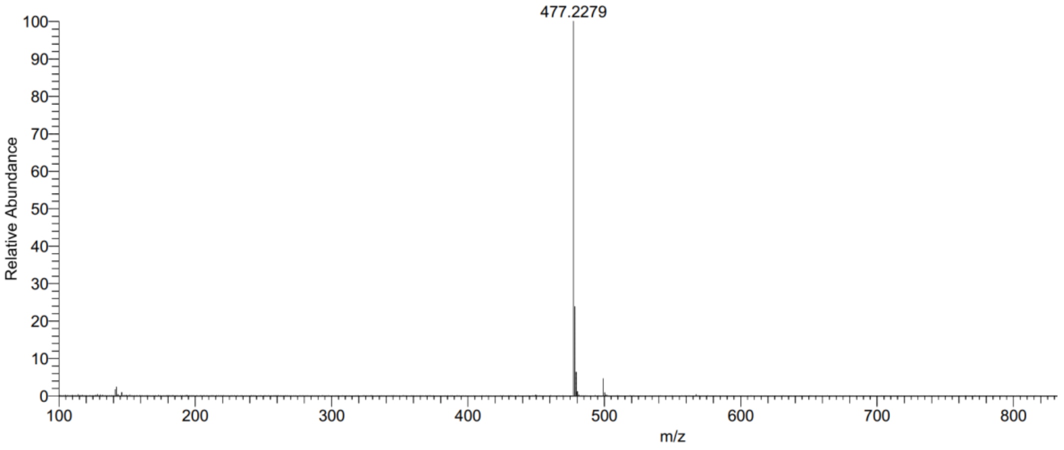

HRMS (ESI): calcd. for $C_{22}H_{33}N_6O_4S$ $[M+H]^+$ = 477.2279; found 477.2279.

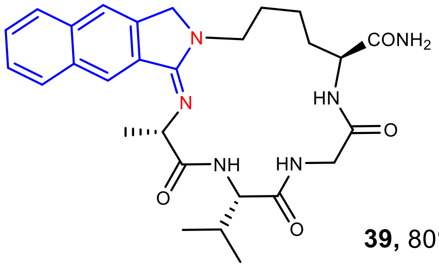

39, 80%

Cyclic peptide **39** (HCOOH salt, 0.016 mmol, 9.2 mg) was prepared as a single isomer in 80% yield from the linear precursor **25** (TFA salt, 0.02 mmol, 11.3 mg) under the condition **C**.

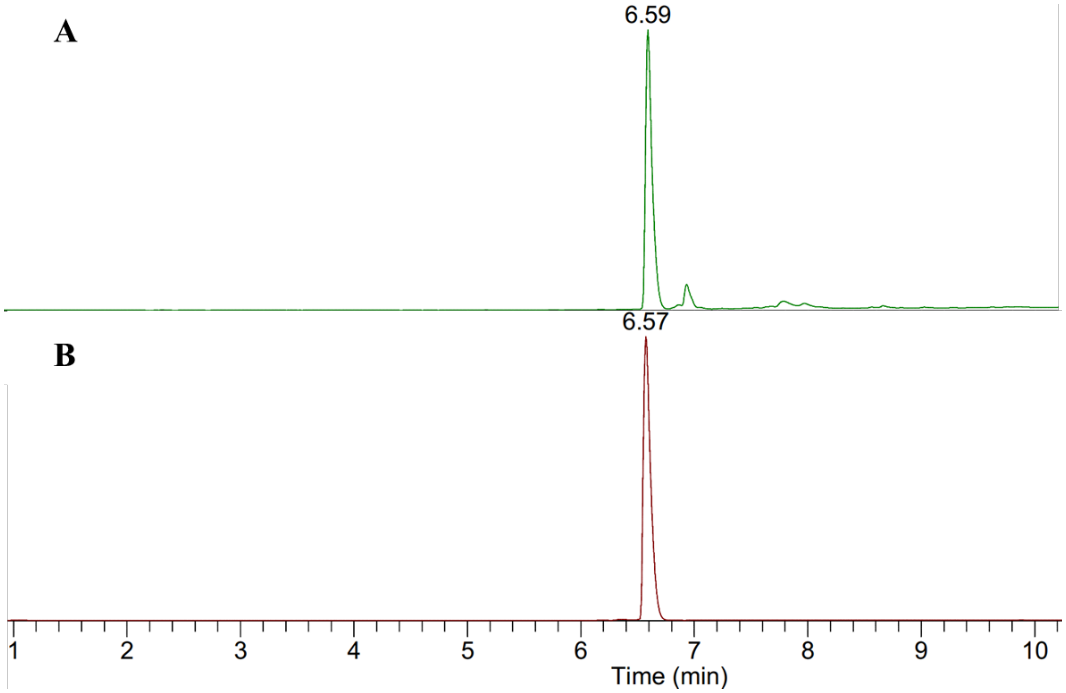

Supplementary Fig. 27. A) Crude UPLC trace of reaction. **B)** UPLC trace of purified product **39** (rt = 6.57 min, λ = 254 nm).

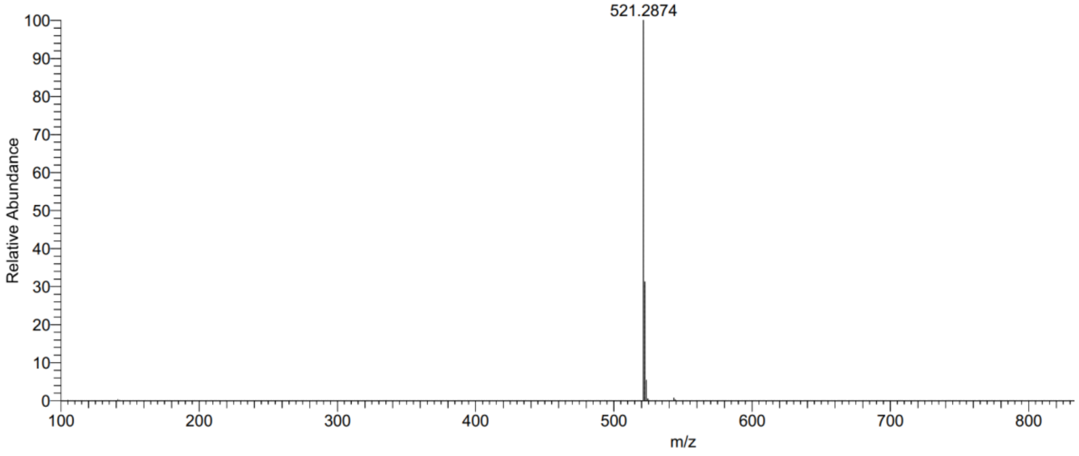

HRMS (ESI): calcd. for $C_{28}H_{37}N_6O_4$ $[M+H]^+$= 521.2871; found 521.2874.

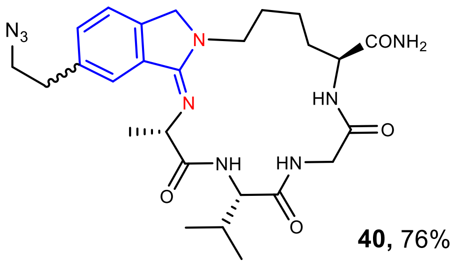

40, 76%

Cyclic peptide **40** (HCOOH salt, 0.015 mmol, 8.7 mg) were prepared in 76% yield from the linear precursor **25** (TFA salt, 0.02 mmol, 11.3 mg) under the condition **C**.

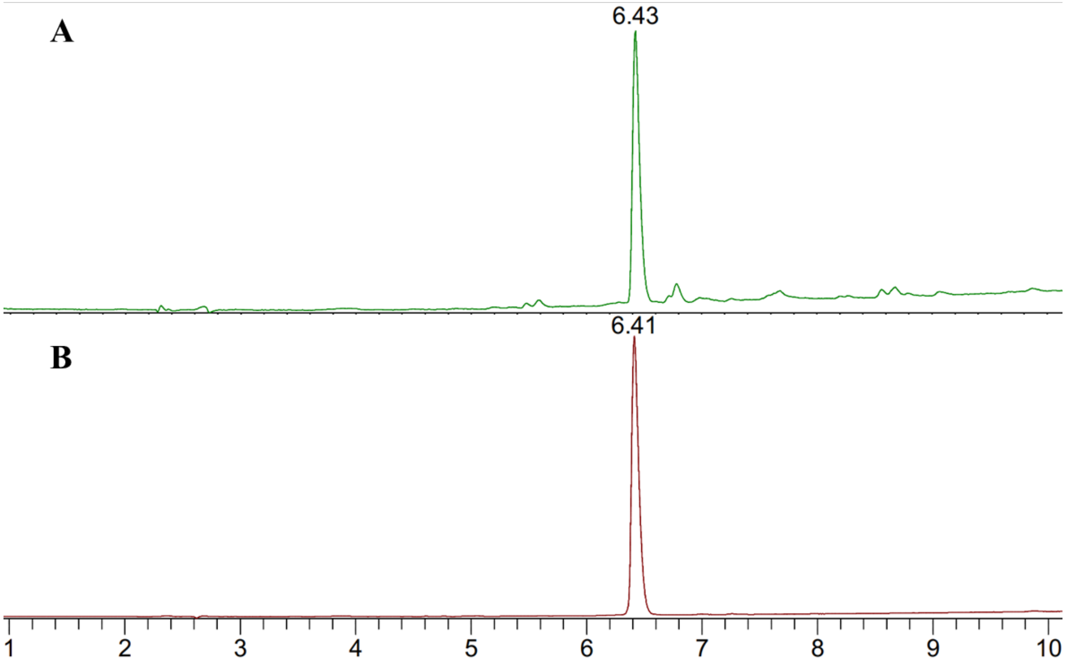

Supplementary Fig. 28. A) Crude UPLC trace of reaction. **B)** UPLC trace of purified product **40** (rt = 6.41 min, λ = 254 nm).

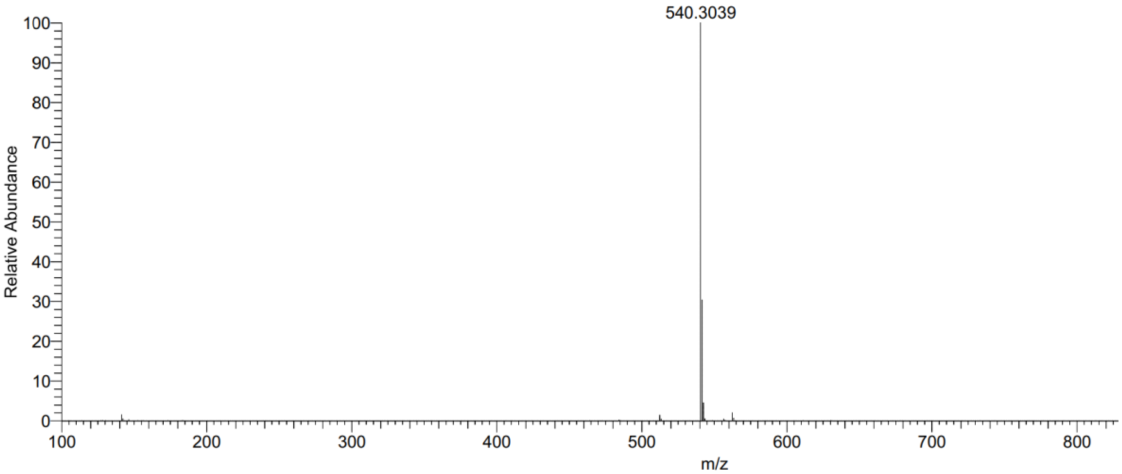

HRMS (ESI): calcd. for $C_{26}H_{38}N_9O_4$ $[M+H]^+$= 540.3041; found 540.3039.

Cyclization on solid phase

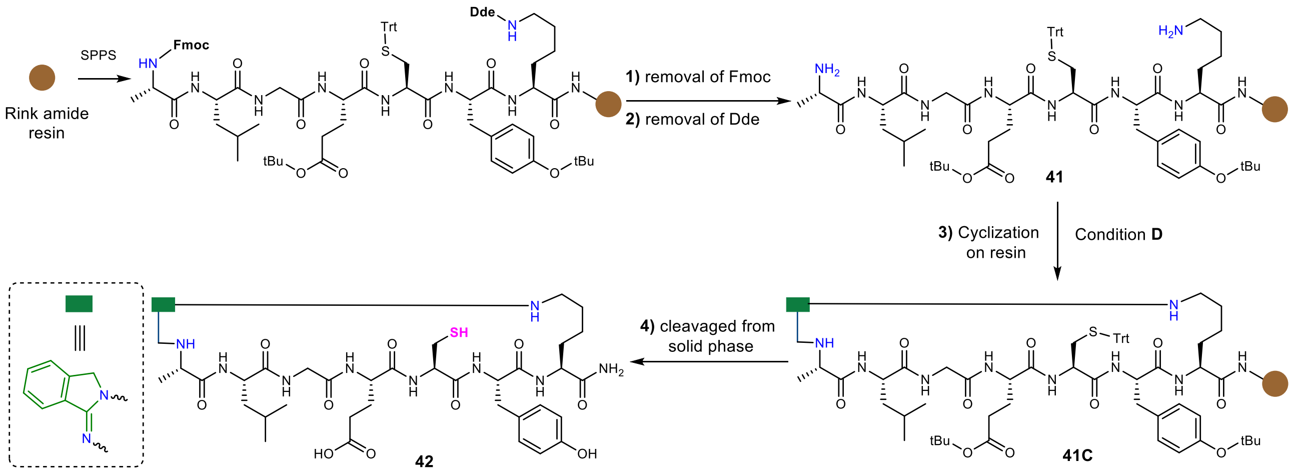

Supplementary Fig. 29. On-resin cyclization.

1) removal of Fmoc:

Deprotection of Fmoc was conducted by treatment with 20% piperidine/DMF twice for each 10min.

2) removal of Dde:

Selective removal of Dde was conducted with 5% $N_2H_4 \cdot H_2O$/DMF twice for each 10 min at rt.

The peptidyl-resin was placed in a solid-phase synthesis tube and treated with 5% $N_2H_4 \cdot H_2O$/DMF. Then stopper the tube and leave to stand at rt for 10 min. Filter the resin and repeat the hydrazine deprotection.

3) Cyclization on resin:

OPA (1.2 equiv, stock solution) and DIPEA (3.0 equiv) was added to the swelled resin in 5 ml of TFE, then the tube was shaken for for 10 min at rt.

Note: The resin was first swelled by DCM, and the TFE was added after suction of DCM.

4) Cleavaged from solid phase:

The dry Rink Amide MBHA resin was treated with a cocktail of TFA/TIPS/H_2O (95/2.5/2.5, v/v/v) for 2 hours. The solvents were dried under N_2 or Ar stream, after which the cold diethyl ether was added to precipitate the crude product **42**. After centrifugation the supernatant was taken out and purification was conducted by semi-preparative HPLC.

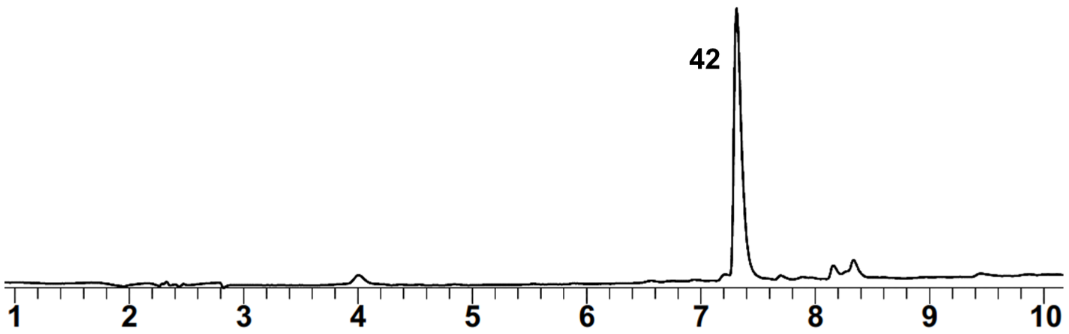

UPLC of crude reaction.

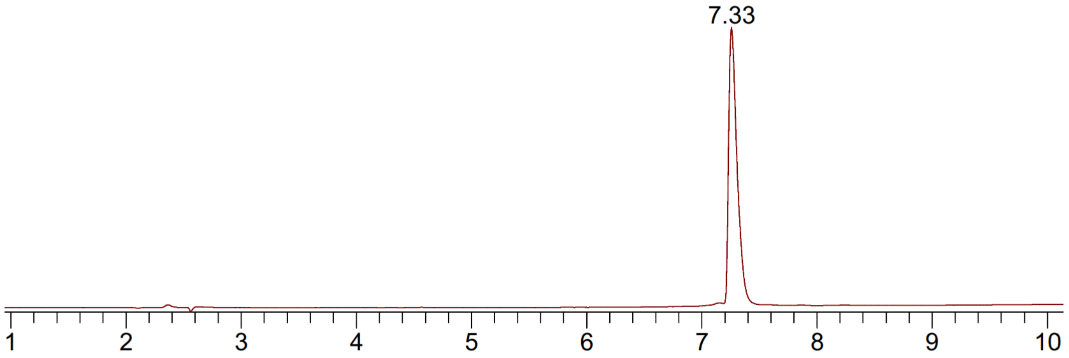

Supplementary Fig. 30. UPLC trace of purified product **42** (rt = 7.33 min, λ = 254 nm).

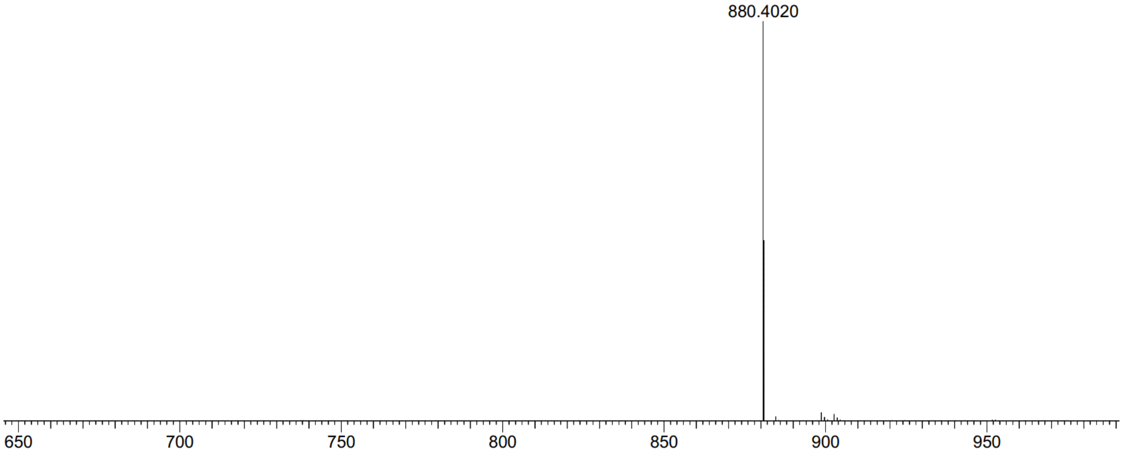

HRMS (ESI): calcd. for $C_{42}H_{58}N_9O_{10}S$ $[M+H]^+$ = 880.4022; found 880.4020.

Characterization data for side-to-side macrocyclization

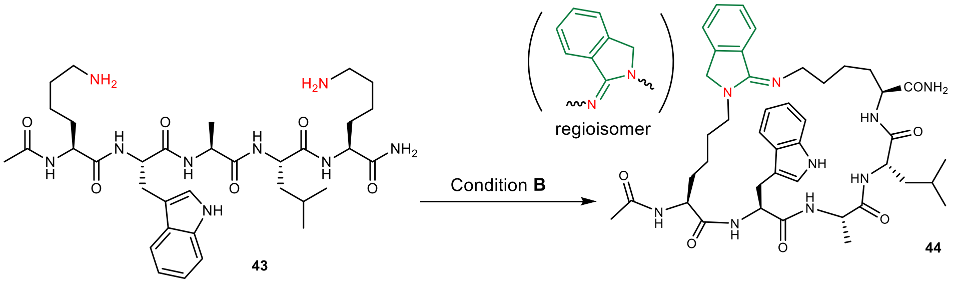

Cyclic peptide **44** (HCOOH salt, 0.039 mmol, 32.3 mg) were prepared as two isomers in 79% yield from the linear precursor **43** (TFA salt, 0.05 mmol, 44.0 mg) under the condition **B**.

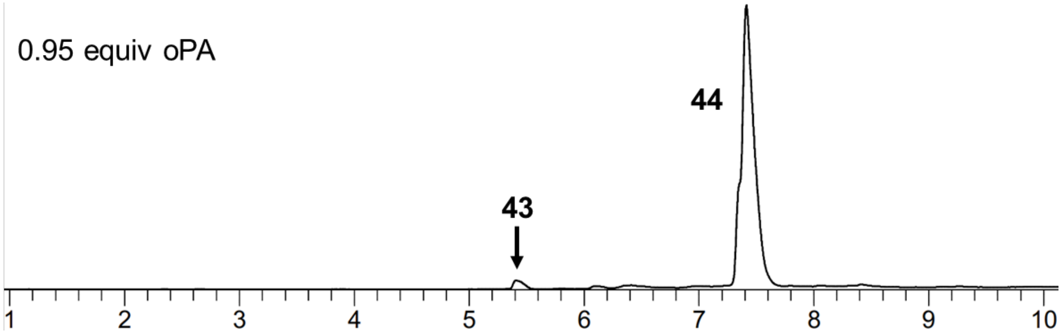

Supplementary Fig. 31. LC trace of the control experiments with 0.95 equiv of OPA (A small amounts of unconsumed peptide substrates **43** can be detected by LC-MS analysis).

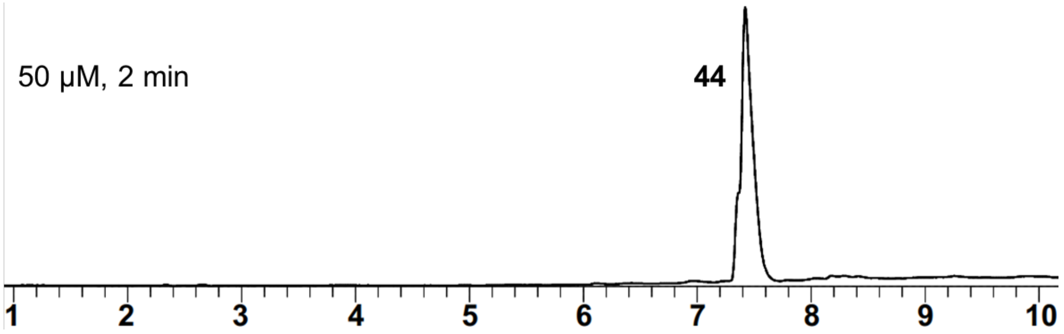

Supplementary Fig. 32. LC trace of the reaction of **43** at 50 μM for 2 min.

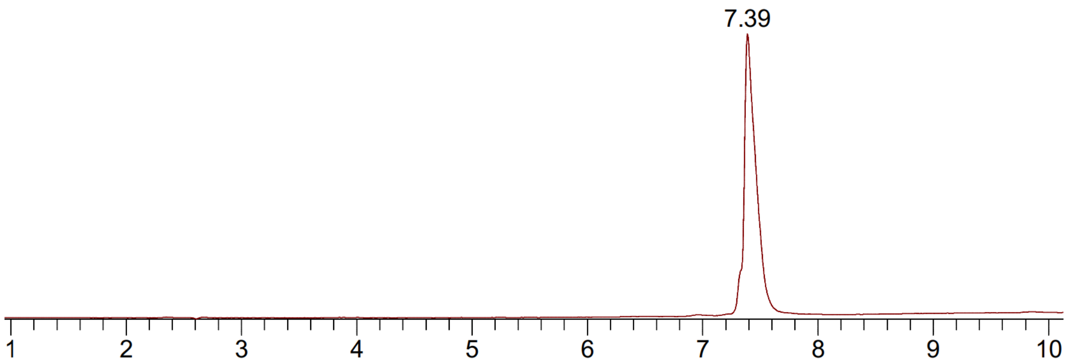

Supplementary Fig. 33. LC trace of purified product **44** (rt =7.39 min, λ = 280 nm).

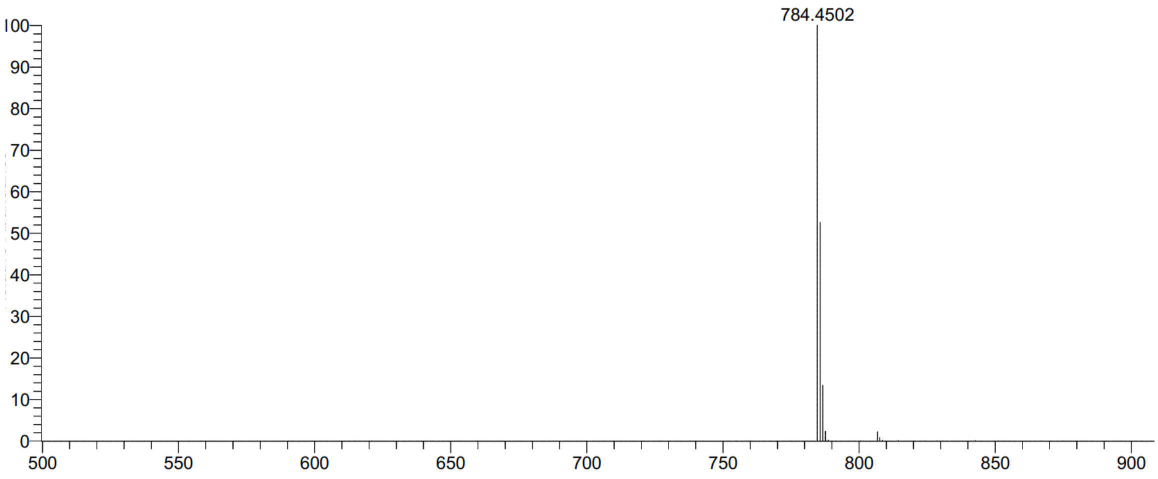

HRMS (ESI): calcd. for $C_{42}H_{58}N_9O_6$ $[M+H]^+$= 784.4505; found 784.4502.

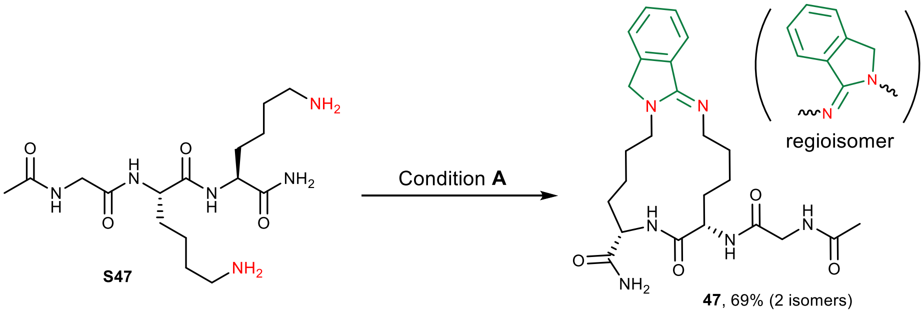

Cyclic peptide **47** (HCOOH salt, 0.014 mmol, 7.2 mg) were prepared as two isomers in 69% yield from the linear precursor **S47** (TFA salt, 0.02 mmol, 11.4 mg) under the condition **A**.

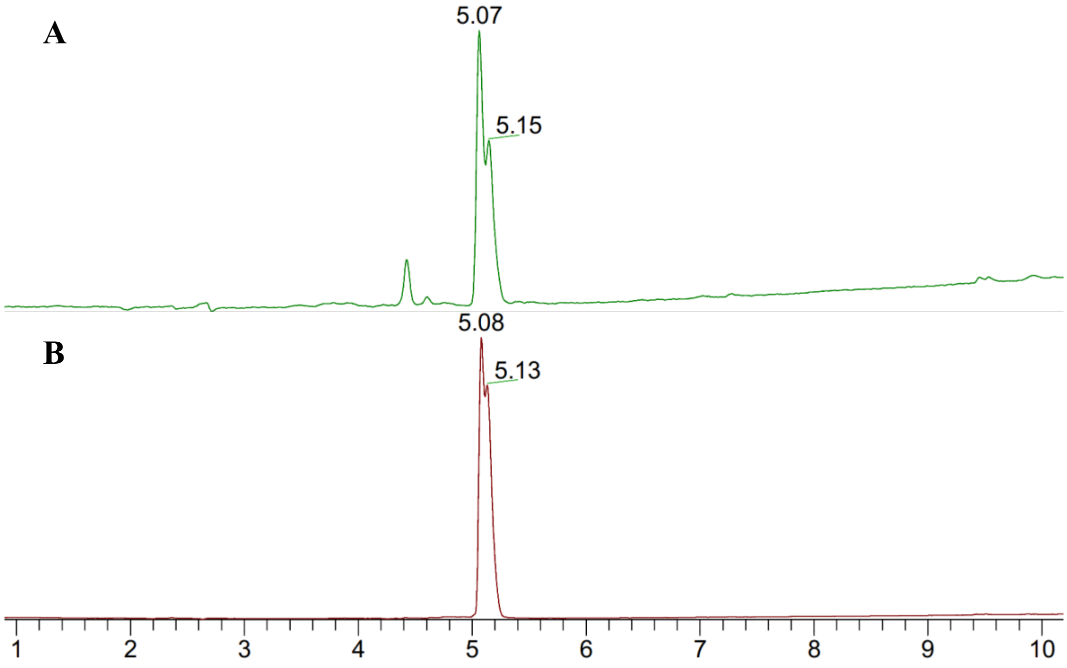

Supplementary Fig. 34. A) Crude UPLC trace of reaction. **B)** LC trace of purified product **47** (rt =5.08 & 5.13 min).

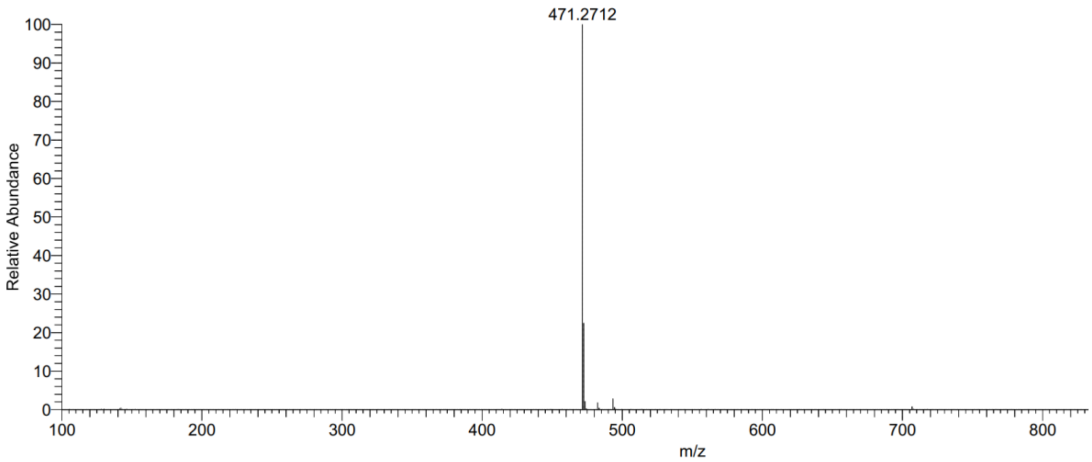

HRMS (ESI): calcd. for $C_{24}H_{35}N_6O_4$ $[M+H]^+$ = 471.2714; found 471.2712.

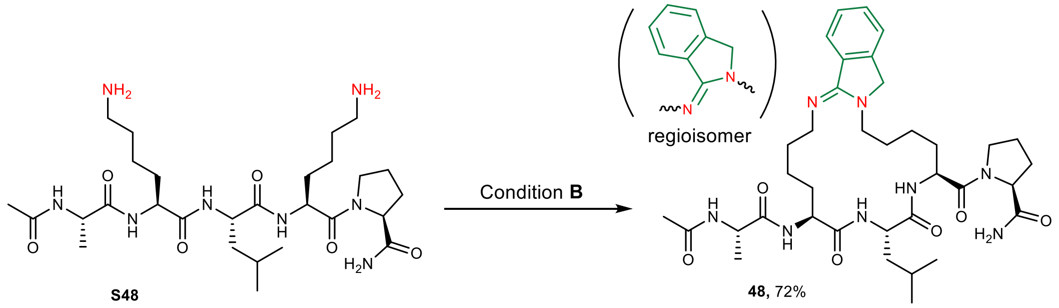

Cyclic peptide **48** (HCOOH salt, 0.014 mmol, 10.6 mg) were prepared as two isomers in 72% yield from the linear precursor **S48** (TFA salt, 0.02 mmol, 16.0 mg) under the condition **B**.

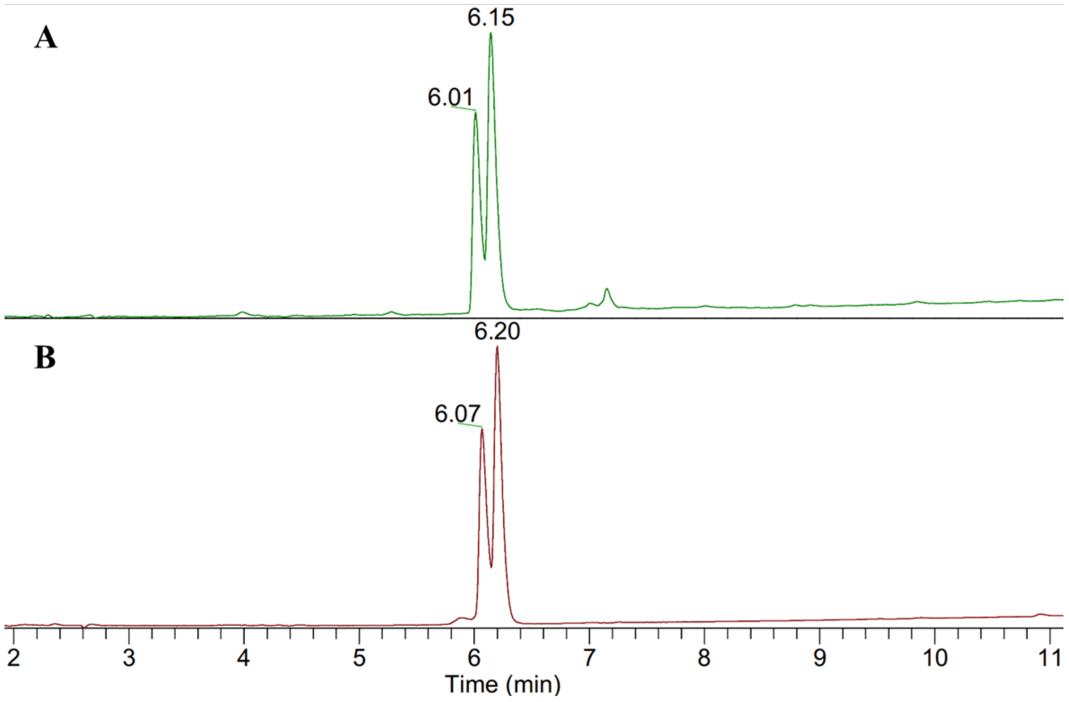

Supplementary Fig. 35. A) Crude UPLC trace of reaction. **B)** LC trace of purified product **48** (rt =6.07, 6.20 min, λ = 254 nm).

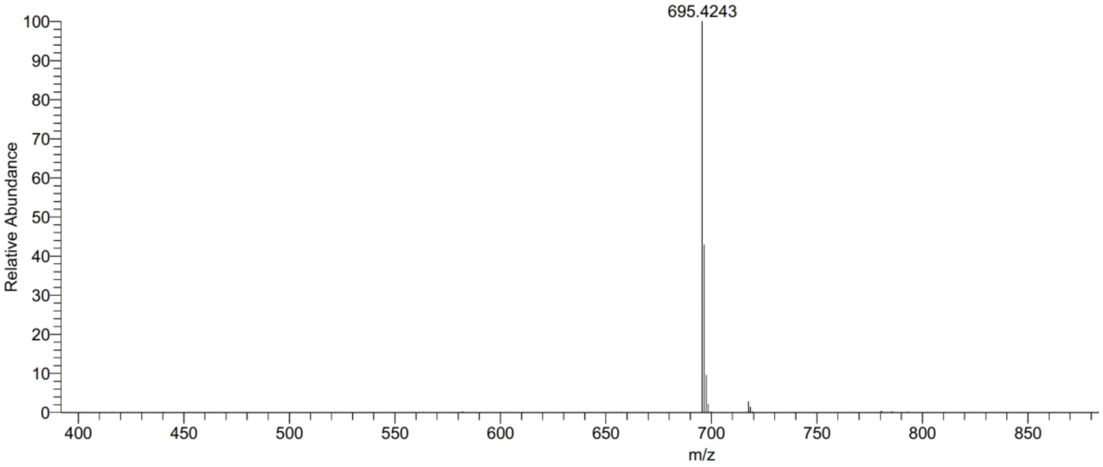

HRMS (ESI): calcd. for $C_{36}H_{55}N_8O_6$ $[M+H]^+$ = 695.4239; found 695.4243.

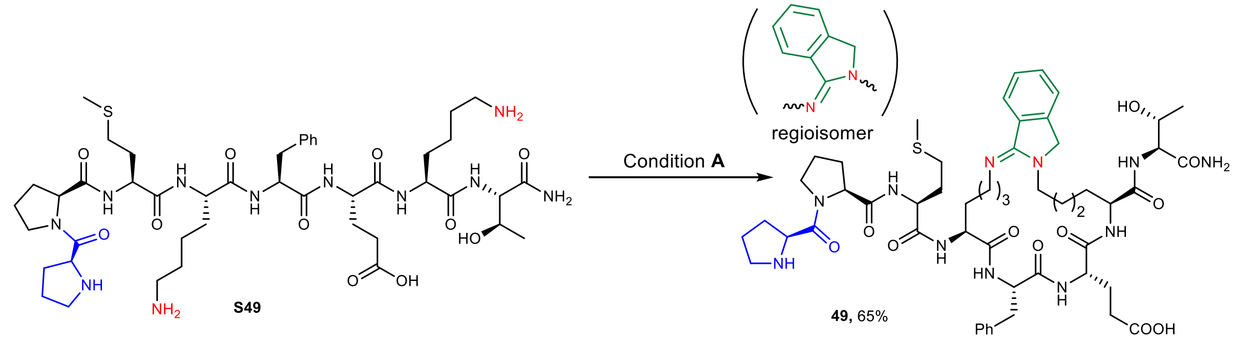

Cyclic peptide **49** (HCOOH salt, 0.013 mmol, 14.6 mg) were prepared as two isomers in 65% yield from the linear precursor **S49** (TFA salt, 0.02 mmol, 23.4 mg) under the condition **A**.

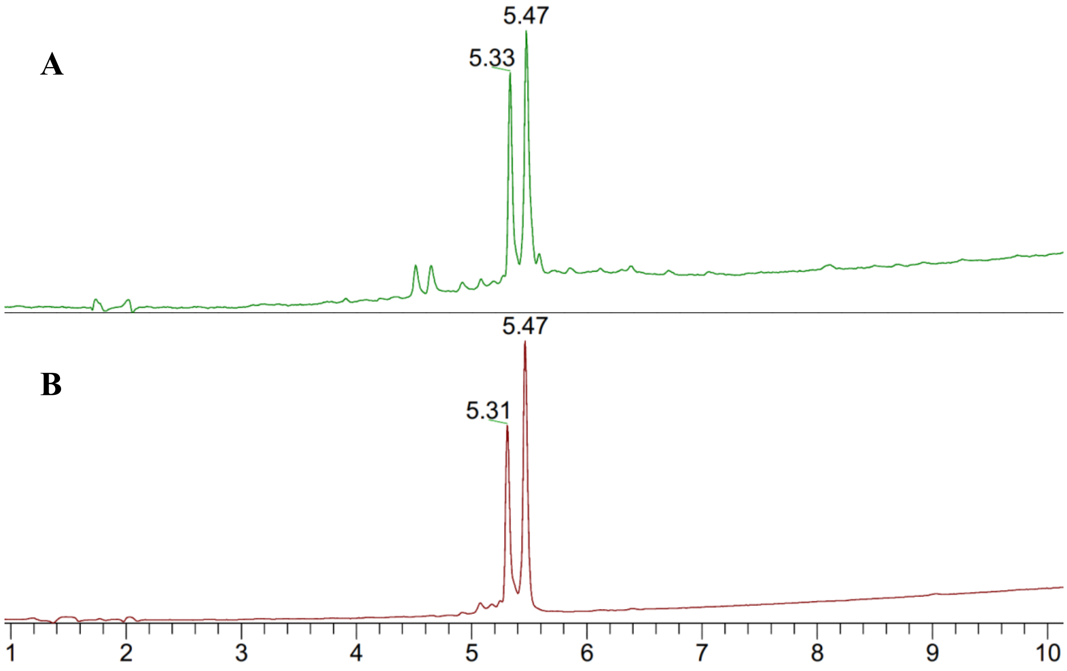

Supplementary Fig. 36. A) Crude UPLC trace of reaction. **B)** LC trace of purified product **49** (rt =5.31, 5.47 min, λ = 254 nm).

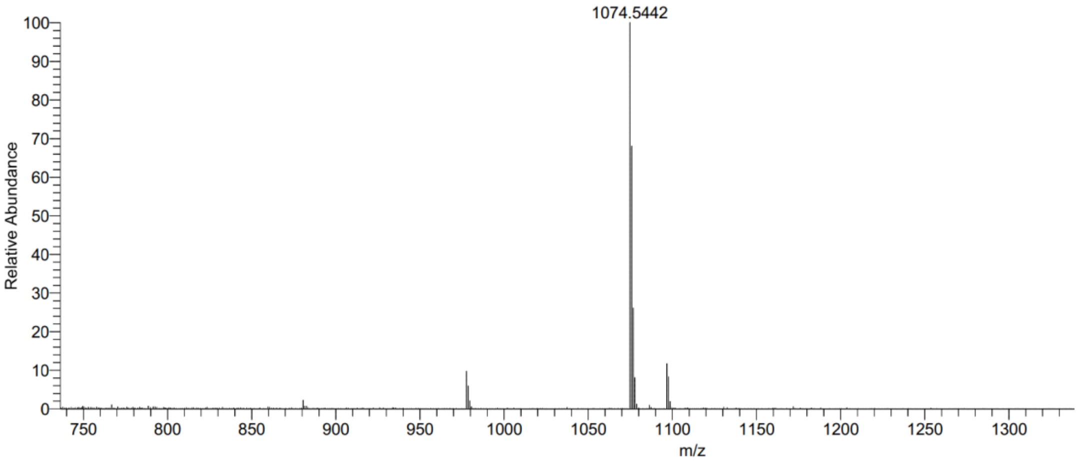

HRMS (ESI): calcd. for $C_{53}H_{76}N_{11}O_{11}S$ $[M+H]^{+}$= 1074.5441; found 1074.5442.

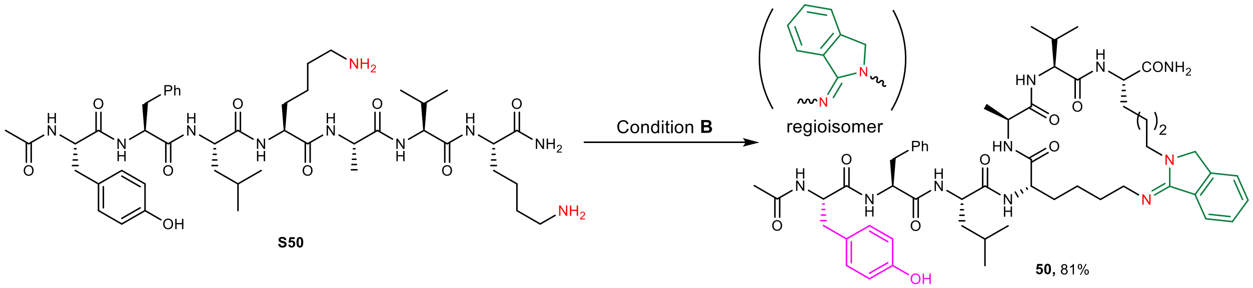

Cyclic peptide **50** (HCOOH salt, 0.016 mmol, 17.1 mg) were prepared as two isomers in 81% yield from the linear precursor **S50** (TFA salt, 0.02 mmol, 22.1 mg) under the condition **B**.

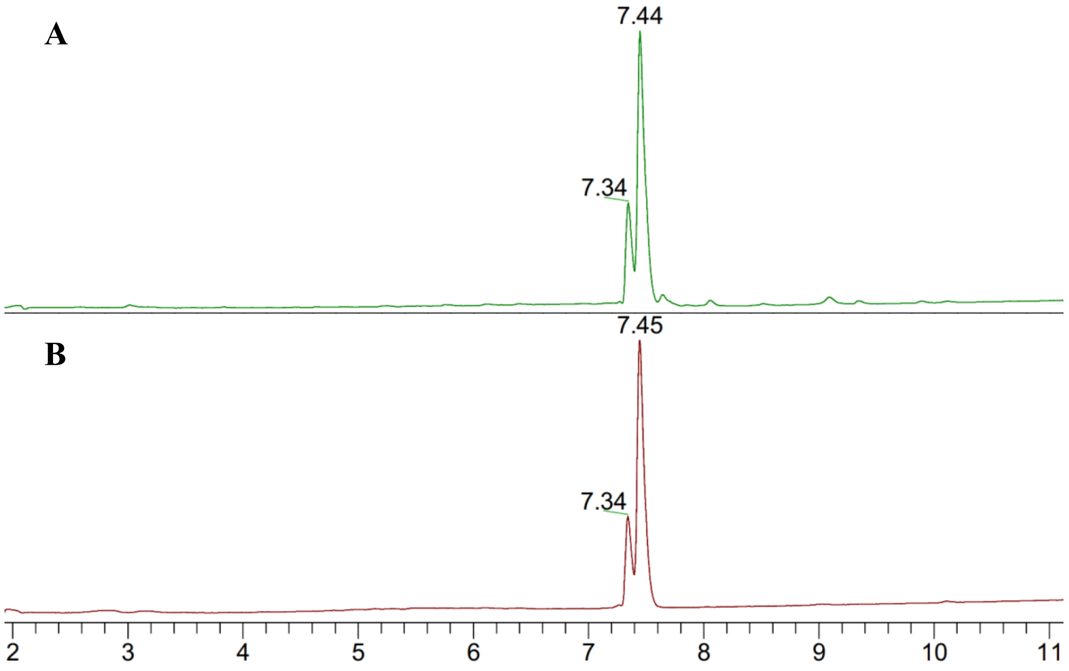

Supplementary Fig. 37. A) Crude UPLC trace of reaction. **B)** LC trace of purified product **50** (rt =7.34, 7.45 min, λ = 254 nm).

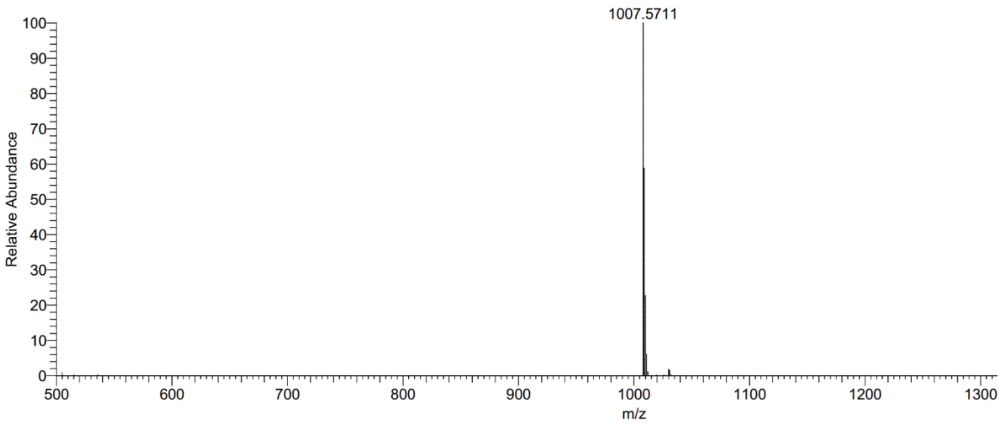

HRMS (ESI): calcd. for $C_{54}H_{75}N_{10}O_{9}$ $[M+H]^{+}$= 1007.5713; found 1007.5711.

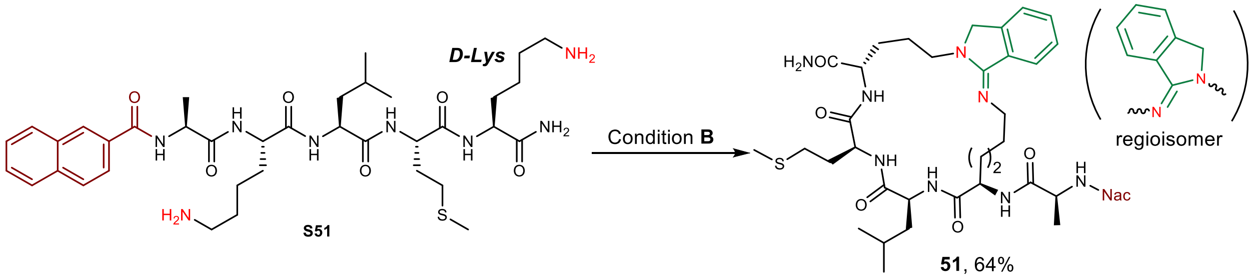

Cyclic peptide **51** (HCOOH salt, 0.013 mmol, 11.4 mg) as separable isomers were prepared in 64% yield from the linear precursor **S51** (TFA salt, 0.02 mmol, 18.8 mg) under the condition **B**.

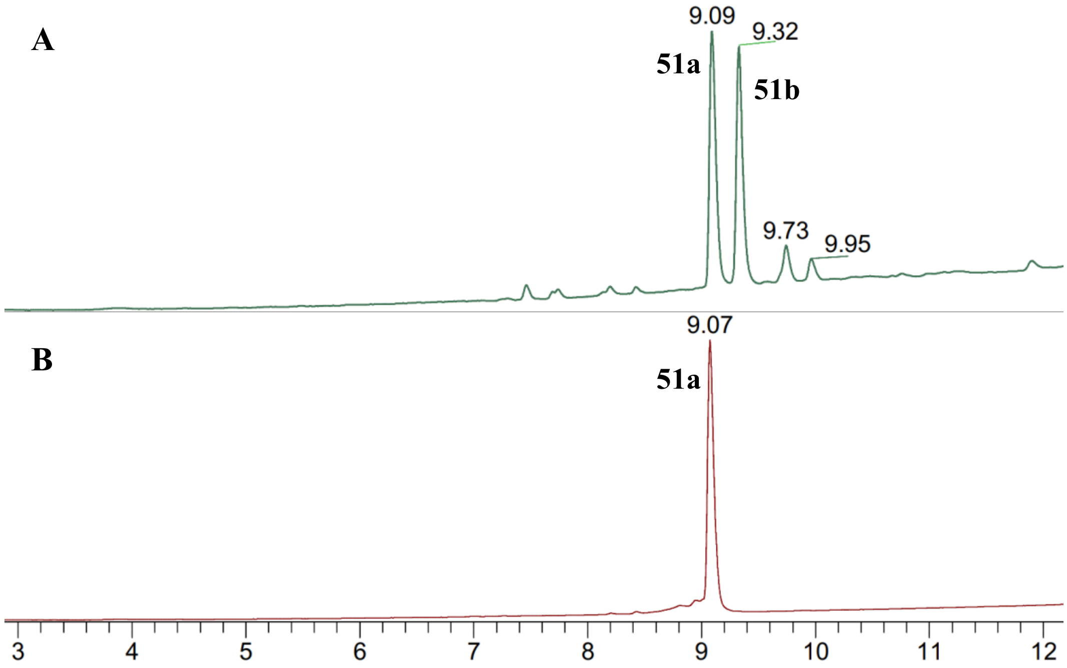

Supplementary Fig. 38. A) Crude UPLC trace of reaction. **B)** LC trace of one of the separated regioisomer **51a** (rt =9.07 min, λ = 254 nm).

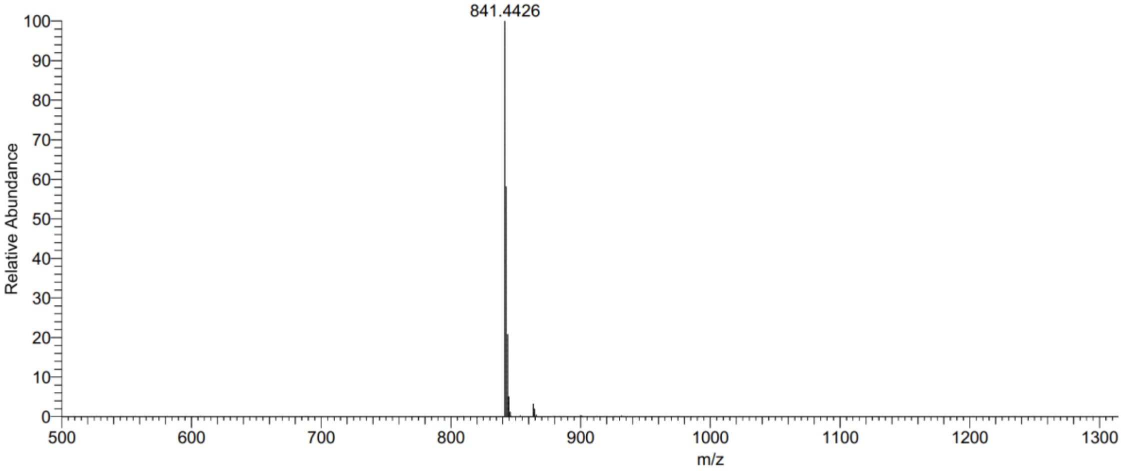

HRMS (ESI): calcd. for $C_{45}H_{61}N_8O_6S$ $[M+H]^+$= 841.4429; found 841.4426.

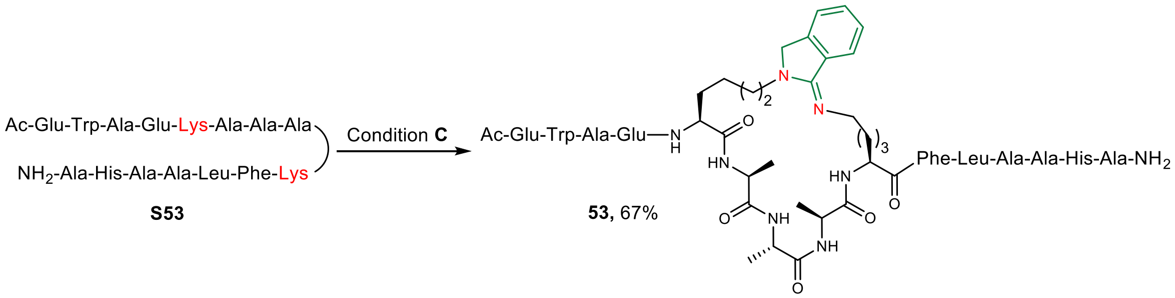

Cyclic peptide **53** (HCOOH salt, 0.0067 mmol, 12.3 mg) were prepared in 67% yield from the linear precursor **S53** (TFA salt, 0.01 mmol, 19.5 mg) under the condition **C**. The 15-mer sequence derived from RNase A, among which two **Lys** replace the two $\mathbf{S_5}$ as the anchors[3].

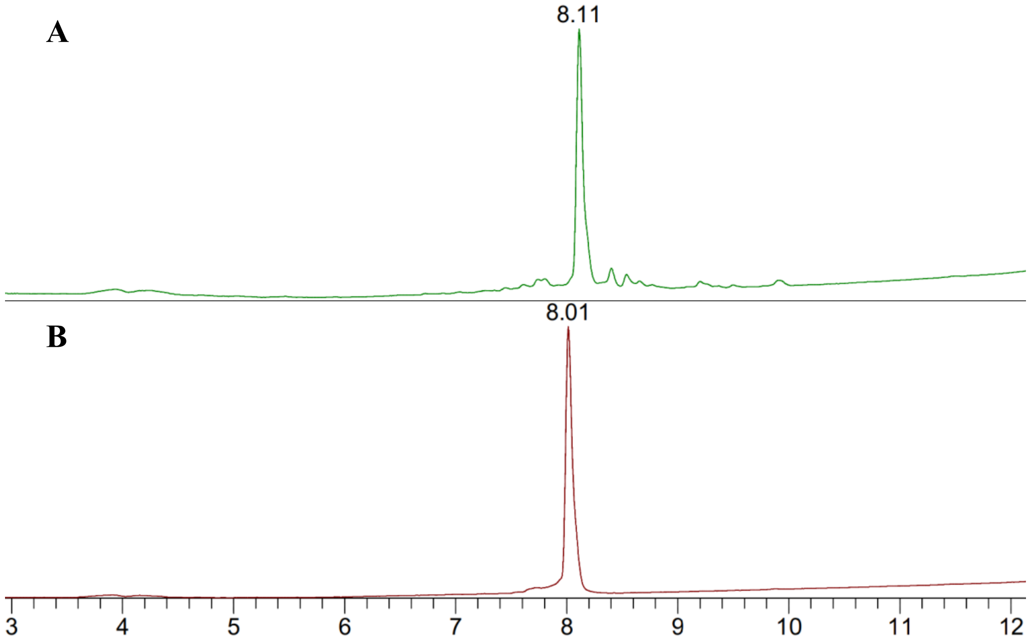

Supplementary Fig. 39. A) Crude UPLC trace of cyclization reaction. **B)** LC trace of purified **53** (rt =8.01 min, λ = 254 nm).

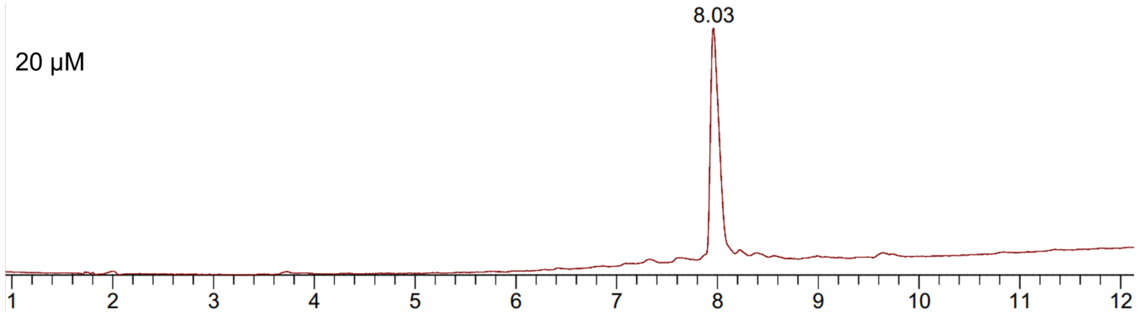

Supplementary Fig. 40. LC of the reaction conducted at 20 μM.

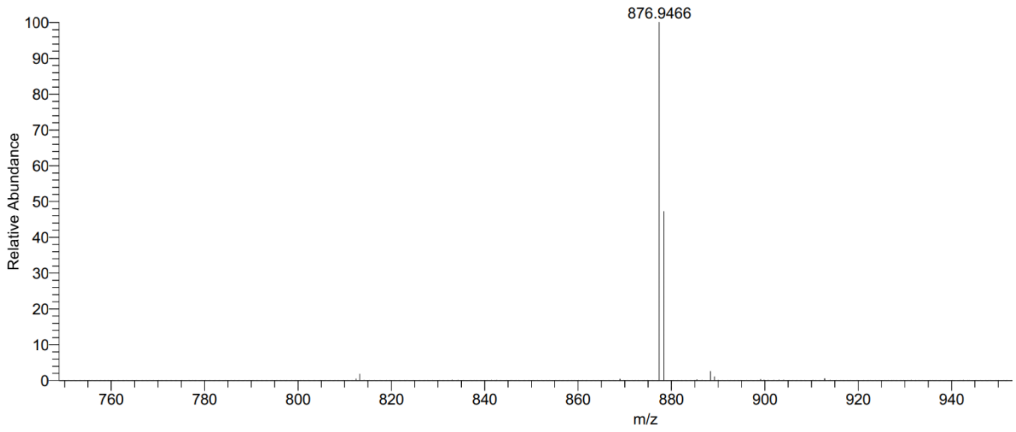

HRMS (ESI): calcd. for $C_{85}H_{119}N_{21}O_{20}$ $[M+2H]^{+}$= 876.9465; found 876.9466.

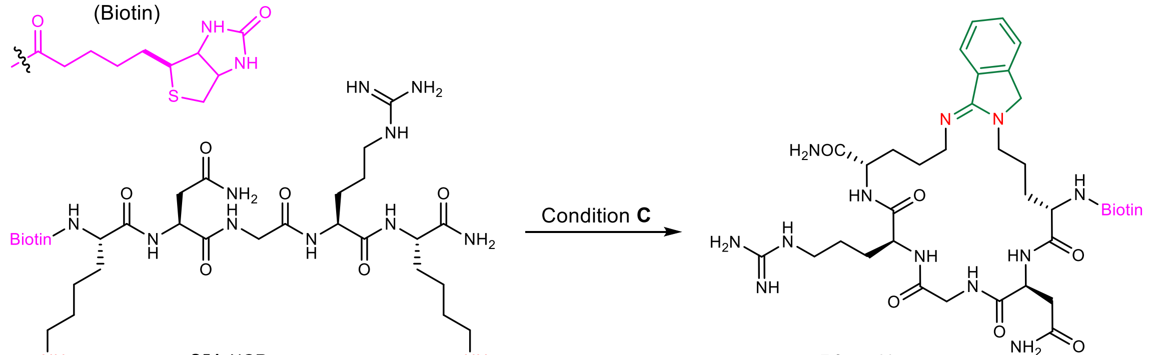

Cyclic peptide **54** (HCOOH salt, 0.0072 mmol, 7.3 mg) as two inseparable isomers were prepared in 72% yield from the linear precursor **S54** (TFA salt, 0.01 mmol, 11.2 mg) under the condition **C**. Biotin was installed on the N-terminus via SPPS using HATU-mediated coupling procedure, and untouched in the cleavage step via TFA in a relatively short period of time.

Note: The NGR sequence was used as an application case because cyclic NGR peptide can selectively binds to the aminopeptidase N (APN or CD13) is known as an attractive tumor marker[4].

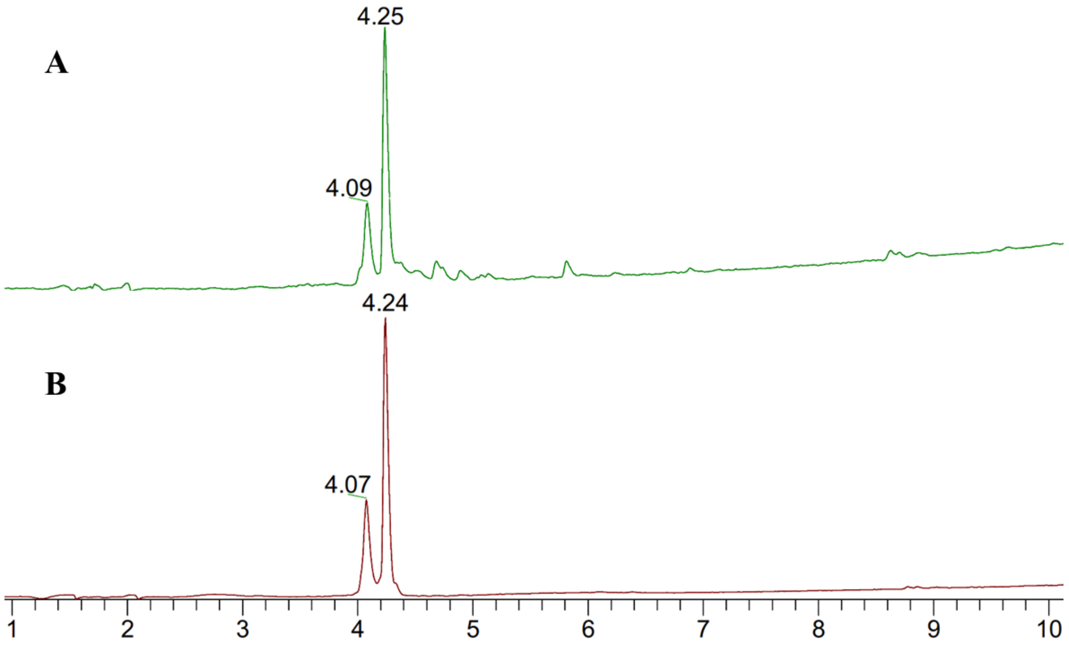

Supplementary Fig. 41. A) Crude UPLC trace of cyclization reaction. **B)** LC trace of purified product **54** (rt =4.07, 4.24 min, λ = 254 nm).

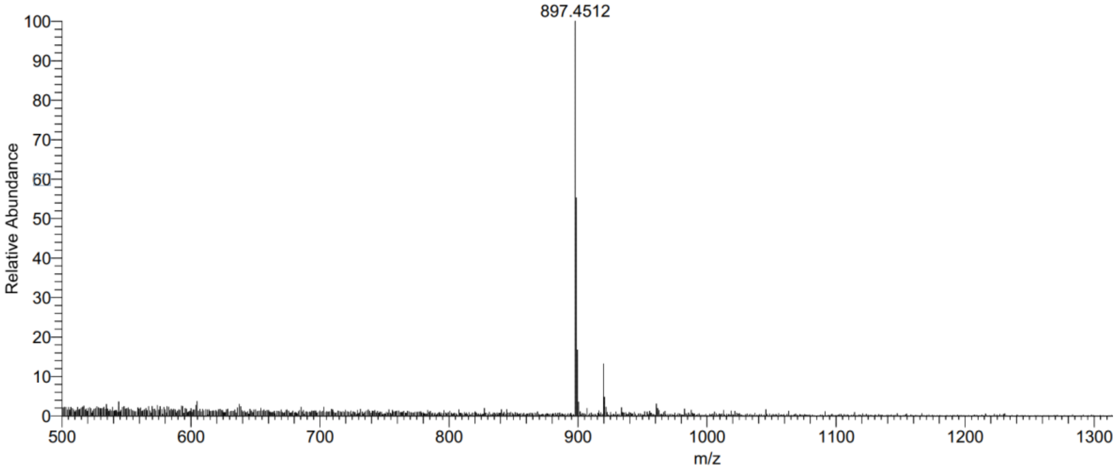

HRMS (ESI): calcd. for $C_{40}H_{61}N_{14}O_8S$ $[M+H]^+$= 897.4512; found 897.4512.

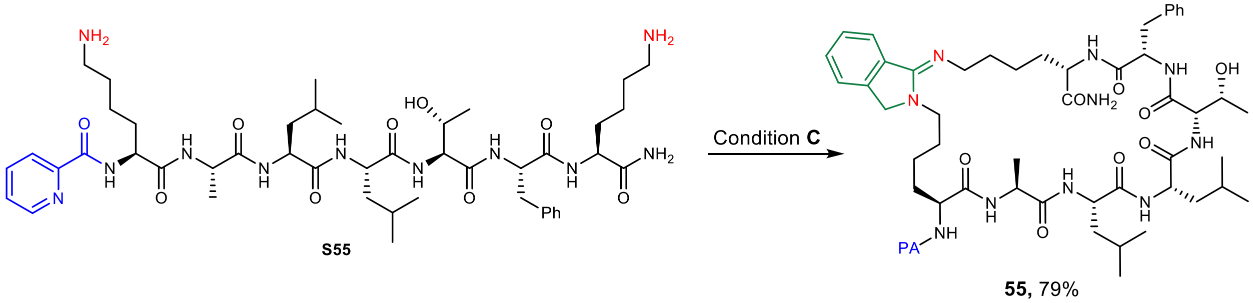

Cyclic peptide **55** (HCOOH salt, 0.0079 mmol, 8.4 mg) as two isomers were prepared in 79% yield from the linear precursor **S55** (TFA salt, 0.01 mmol, 11.2 mg) under the condition **C**.

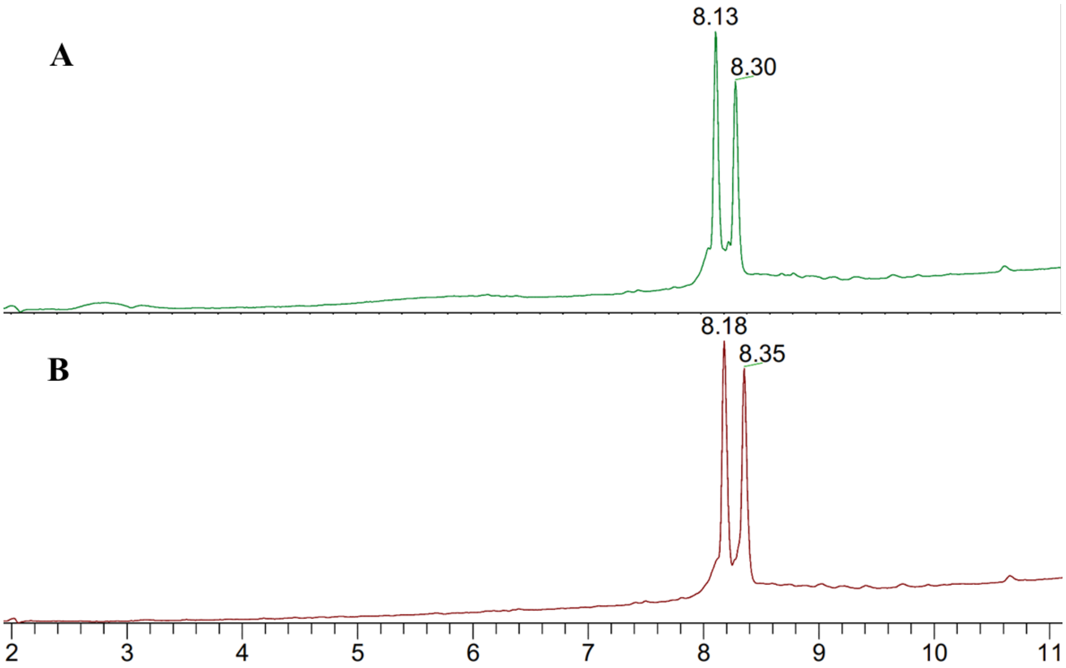

Supplementary Fig. 42. A) Crude UPLC trace of cyclization reaction. **B)** LC trace of purified **55** (rt =8.18, 8.35 min, λ = 254 nm).

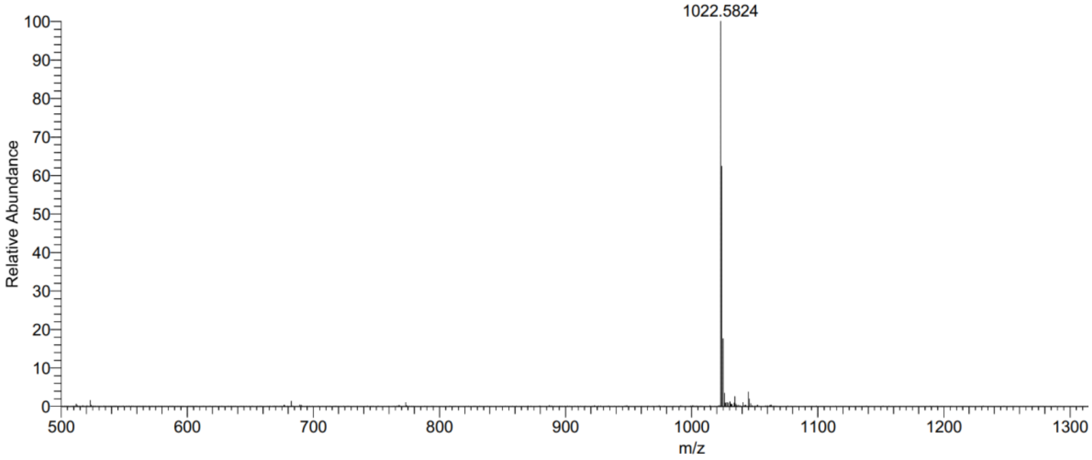

HRMS (ESI): calcd. for $C_{54}H_{76}N_{11}O_9$ $[M+H]^+$= 1022.5822; found 1022.5824.

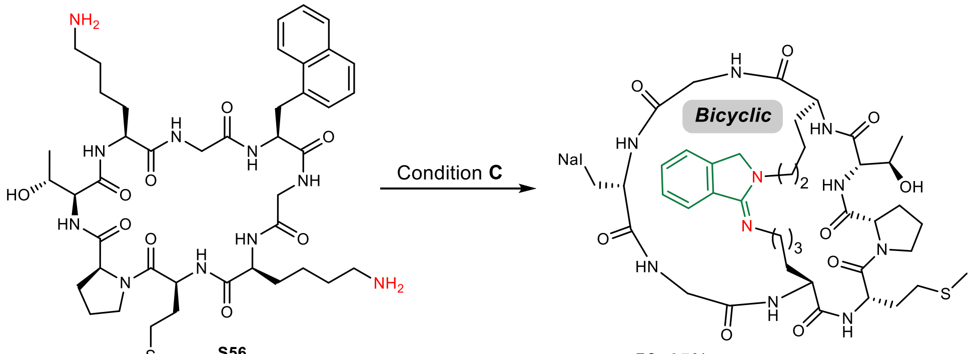

S56

56, 65%

Bicyclic peptide **56** (HCOOH salt, 0.0065 mmol, 6.7 mg) were prepared in 65% yield from the cyclic precursor **S56** (TFA salt, 0.01 mmol, 9.7 mg) under the condition **C**.

S56 was prepared as follow:

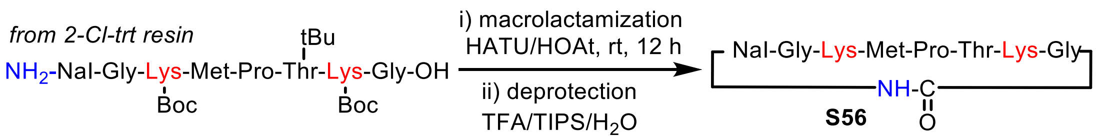

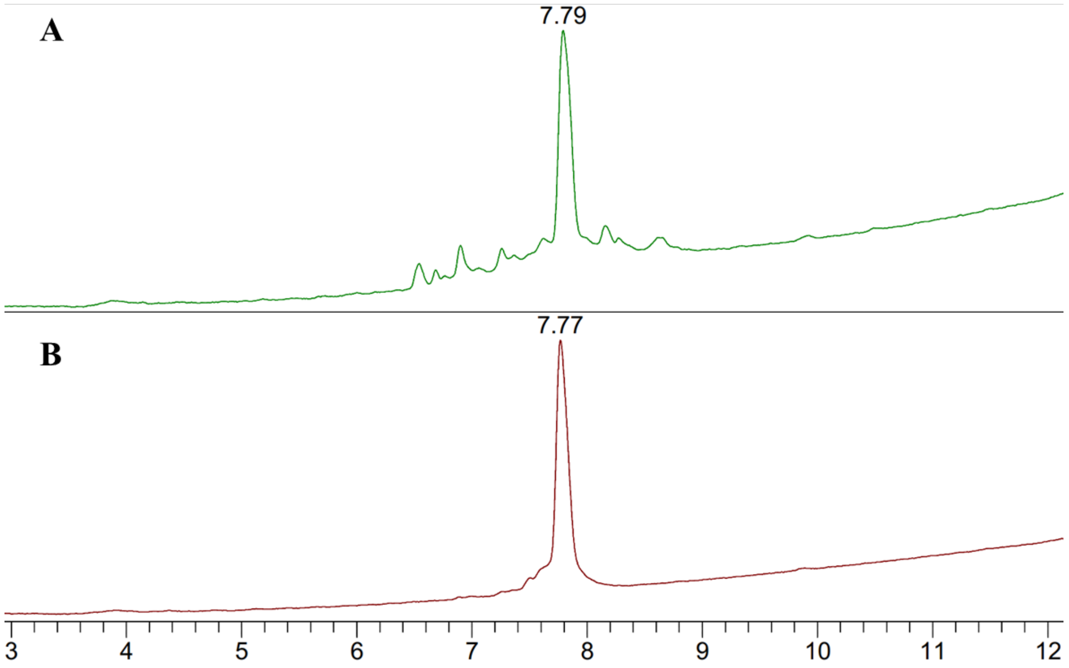

Supplementary Fig. 43. A) Crude UPLC trace of cyclization reaction. **B)** LC trace of purified **56** (rt =7.77 min, λ = 254 nm).

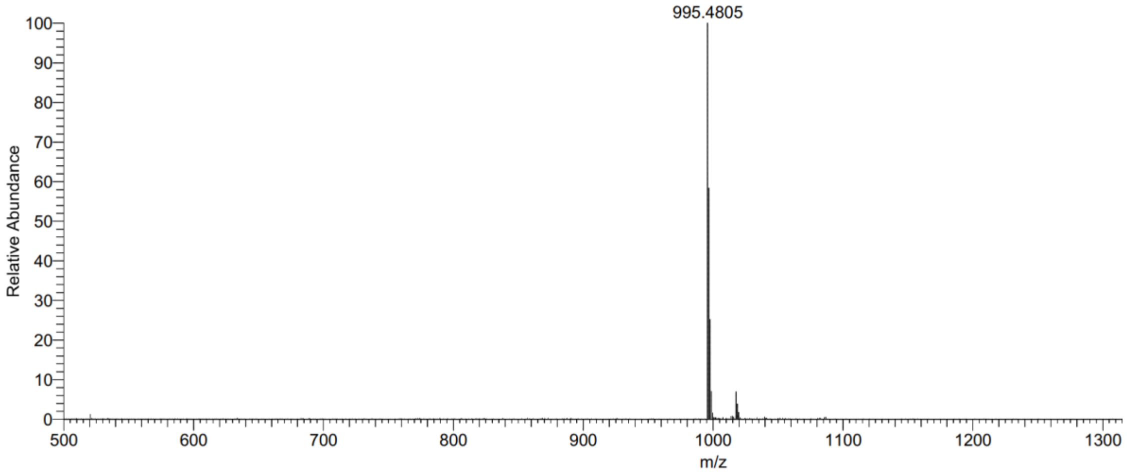

HRMS (ESI): calcd. for $C_{51}H_{67}N_{10}O_9S$ $[M+H]^+$ = 995.4808; found 995.4805.

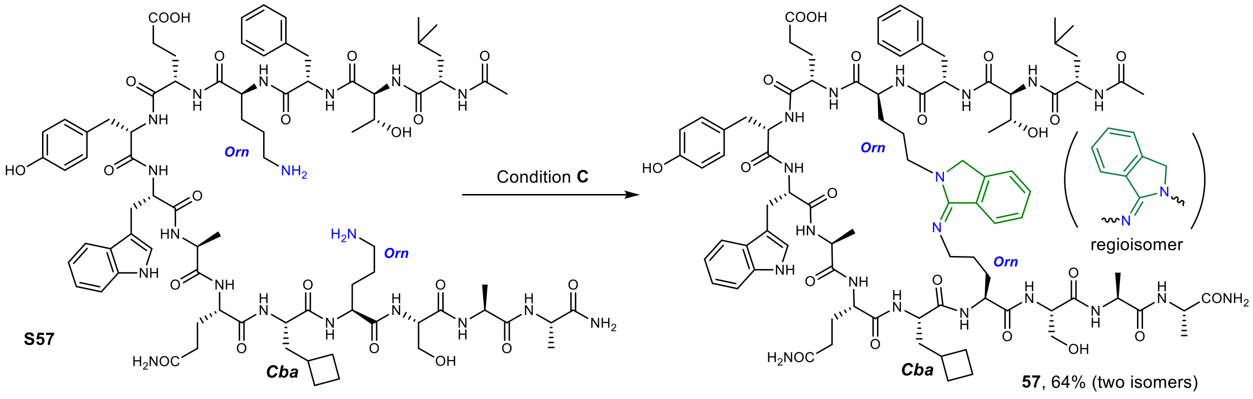

Cyclic peptide **57** (HCOOH salt, 0.0064 mmol, 11.7 mg) as two inseparable isomers were prepared in 64% yield from the linear precursor **S57** (TFA salt, 0.01 mmol, 18.8 mg) under the condition **C**. The 14-mer sequence derived from stapled peptide ATSP-7041, among which two **Orn** replace the $\mathbf{R_8}$ and $\mathbf{S_5}$ as the anchors for stapling the α-helical peptide[5].

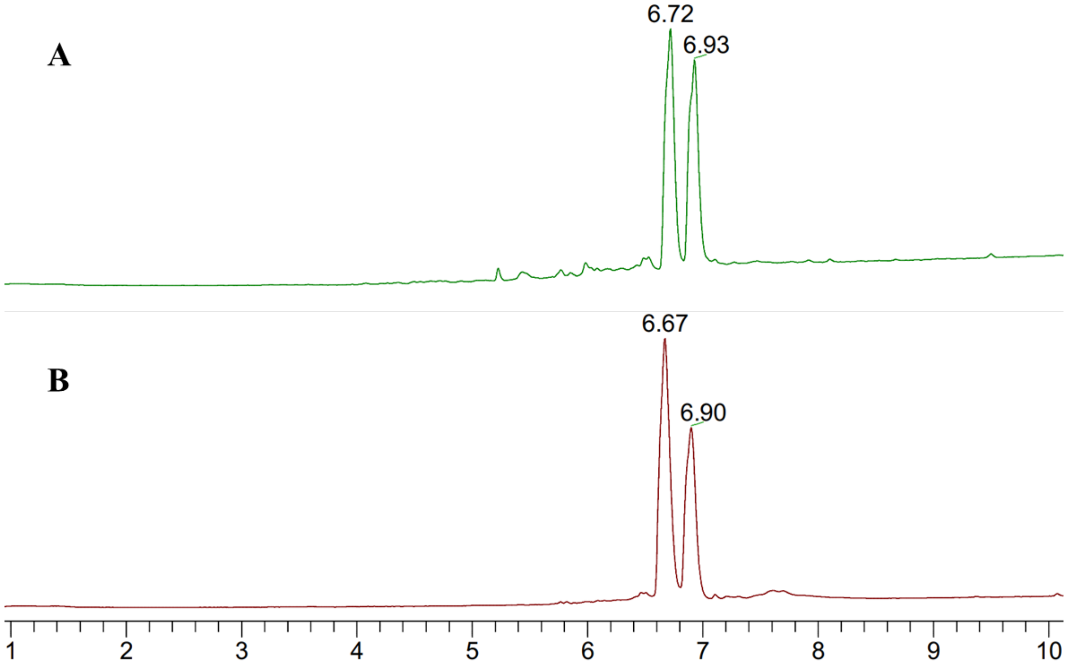

Supplementary Fig. 44. A) Crude UPLC trace of cyclization reaction (at 1 mM). **B)** LC trace of purified product **57** (rt =6.67 & 6.90 min, λ = 254 nm).

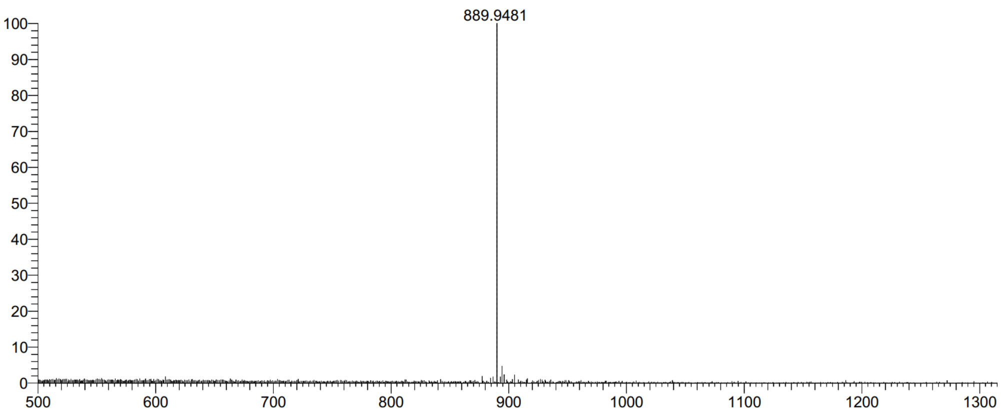

HRMS (ESI): calcd. for $C_{88}H_{121}N_{19}O_{21}$ $[M+2H]^{2+}$= 889.9487; found 889.9481.

Substrates with unblocked N-terminal NH_2

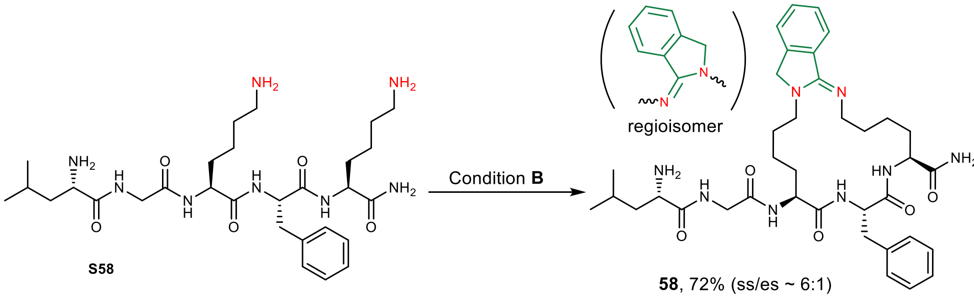

Cyclic peptide **58** (HCOOH salt, 0.0108 mmol, 8.4 mg) were prepared in 72% yield from the linear precursor **S58** (TFA salt, 0.015 mmol, 13.3 mg) under the condition **B**.

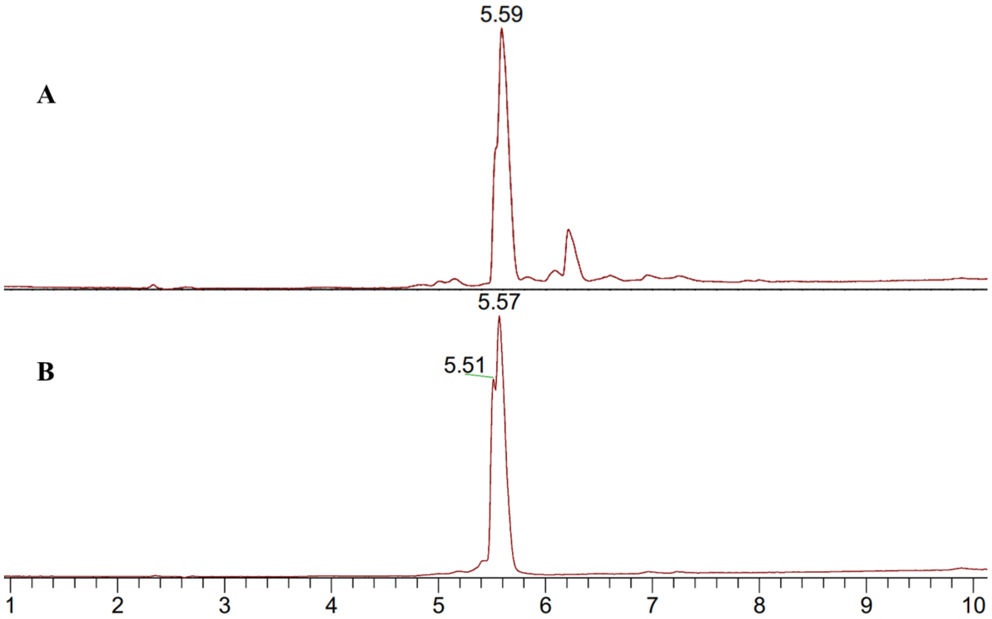

Supplementary Fig. 45. A) Crude UPLC trace of cyclization reaction. **B)** LC trace of purified product **58** (rt = 5.51 & 5.57 min, λ = 254 nm).

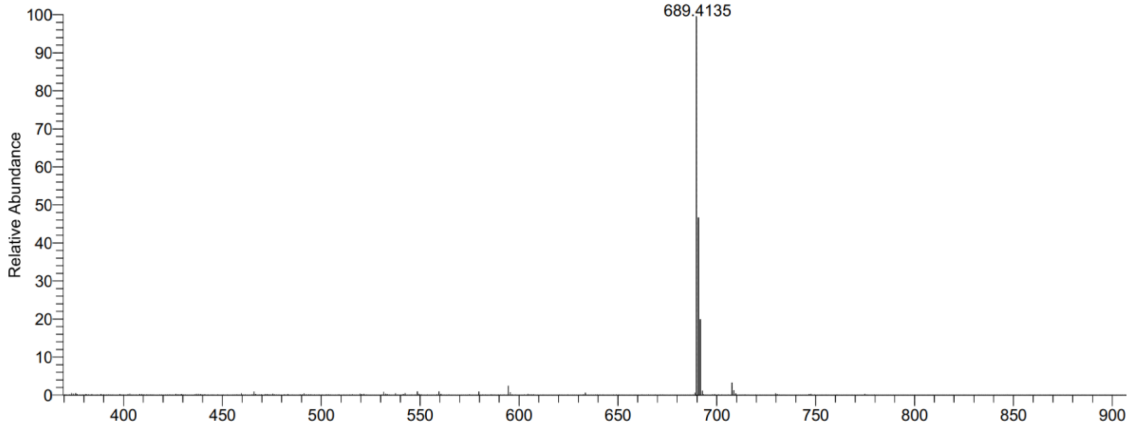

HRMS (ESI): calcd. for $C_{37}H_{53}N_8O_5$ $[M+H]^+$= 689.4133; found 689.4135.

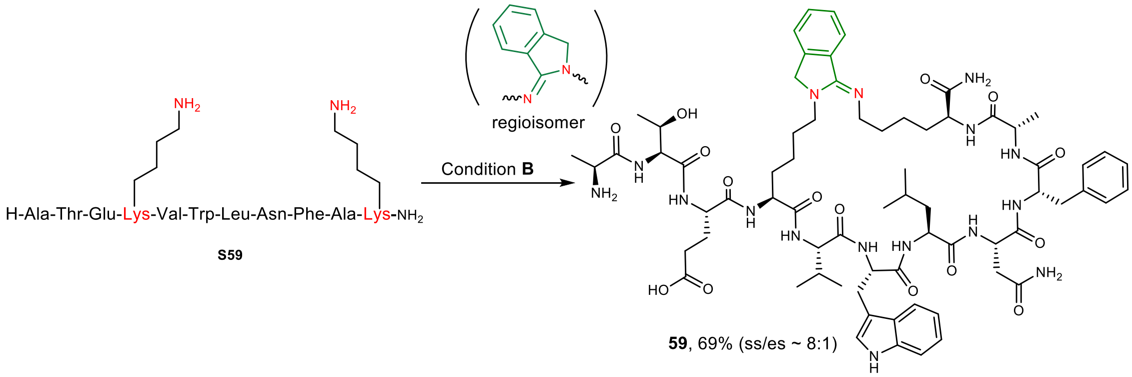

Cyclic peptide **59** (HCOOH salt, 0.0069 mmol, 10.3 mg) were prepared in 69% yield from the linear precursor **S59** (TFA salt, 0.01 mmol, 16.0 mg) under the condition **B**.

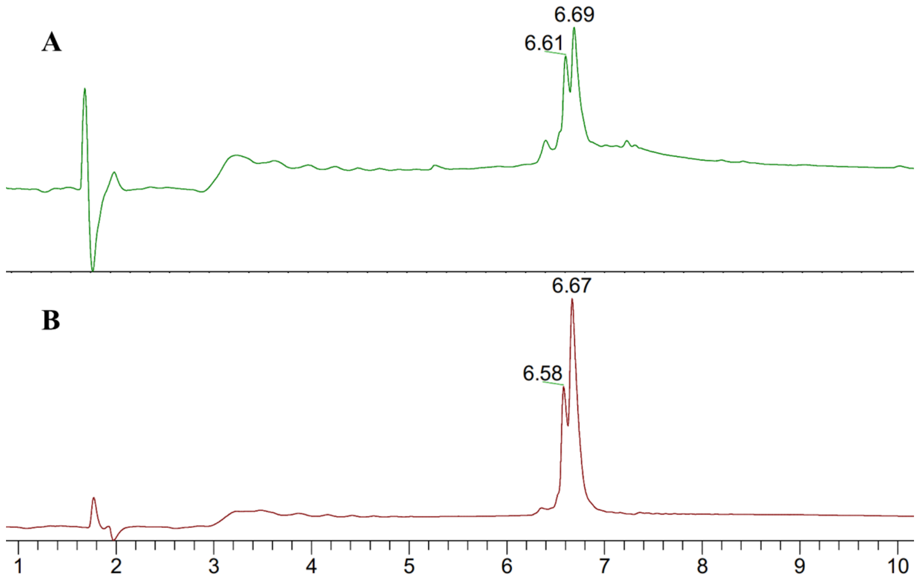

Supplementary Fig. 46. A) Crude UPLC trace of cyclization reaction. **B)** LC trace of purified product **59** (rt = 6.58 & 6.67 min, λ = 254 nm).

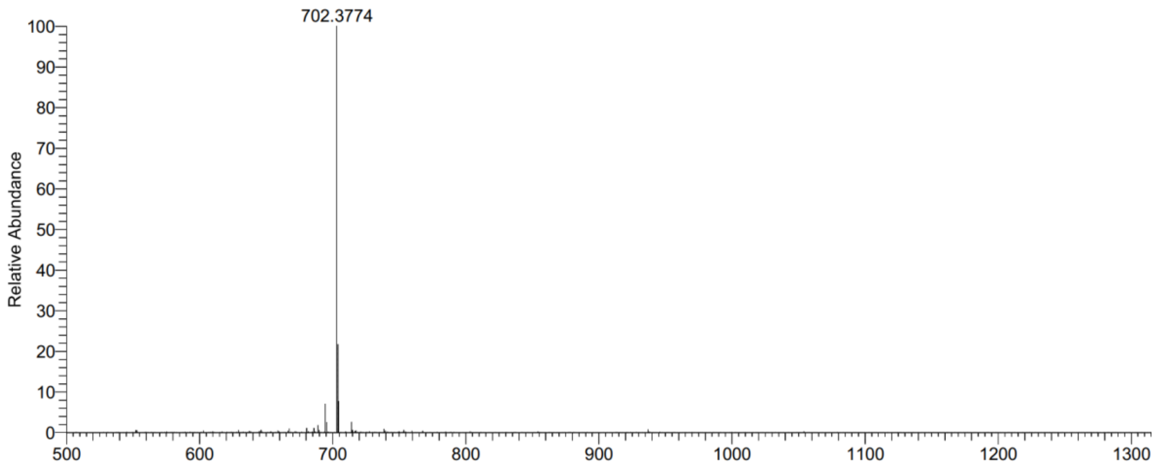

HRMS (ESI): calcd. for $C_{70}H_{100}N_{16}O_{15}$ $[M+2H]^{2+}$= 702.3772; found 702.3774.

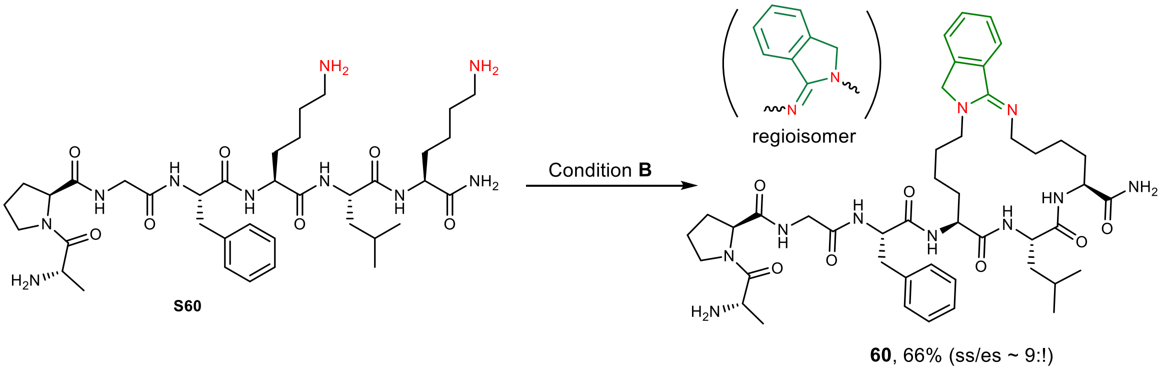

Cyclic peptide **60** (HCOOH salt, 0.0066 mmol, 6.3 mg) were prepared in 66% yield from the linear precursor **S60** (TFA salt, 0.01 mmol, 10.5 mg) under the condition **B**.

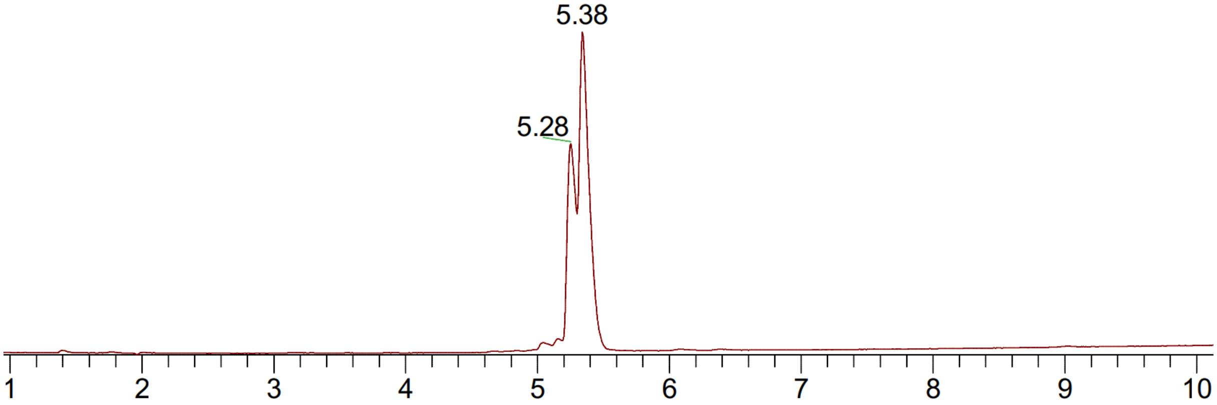

Supplementary Fig. 47. LC of purified product **60** (rt = 5.28 & 5.38 min, λ = 254 nm). Crude HPLC see LC trace below

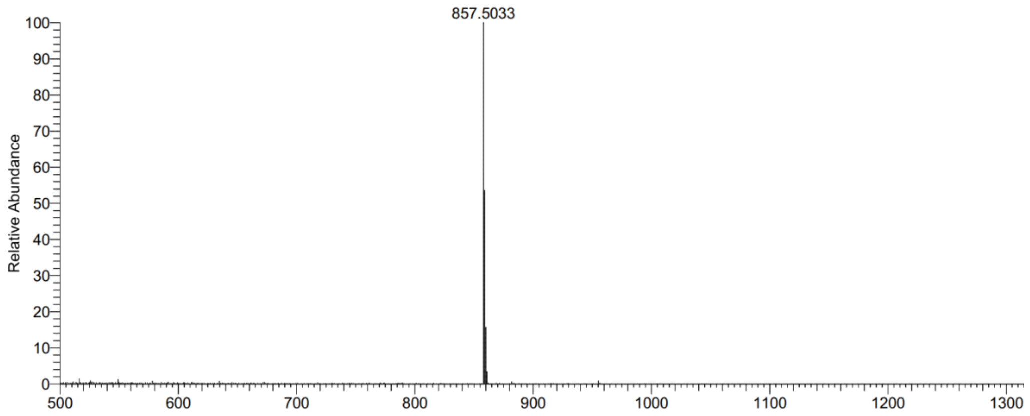

HRMS (ESI): calcd. for $C_{45}H_{65}N_{10}O_7$ $[M+H]^+$= 857.5032; found 857.5033.

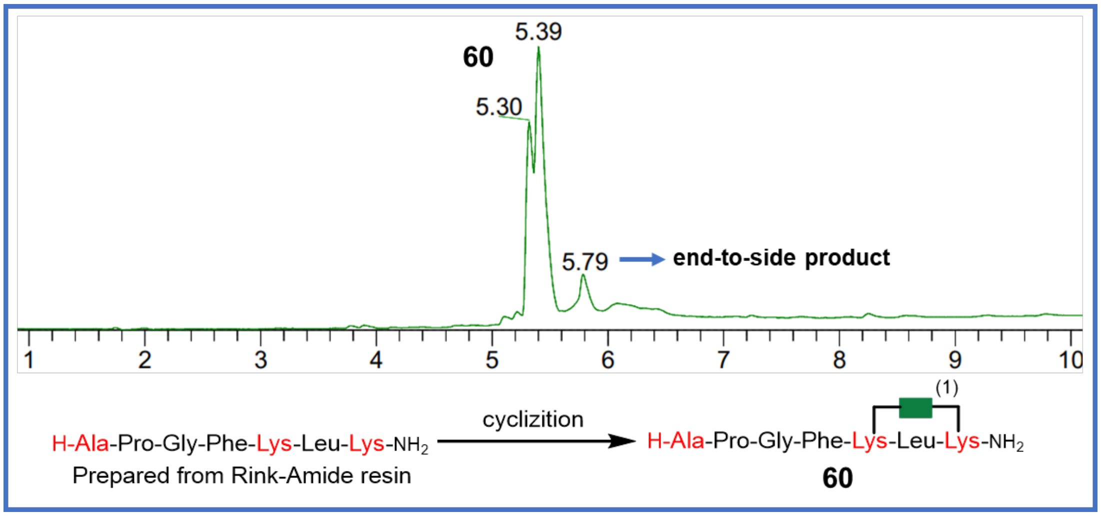

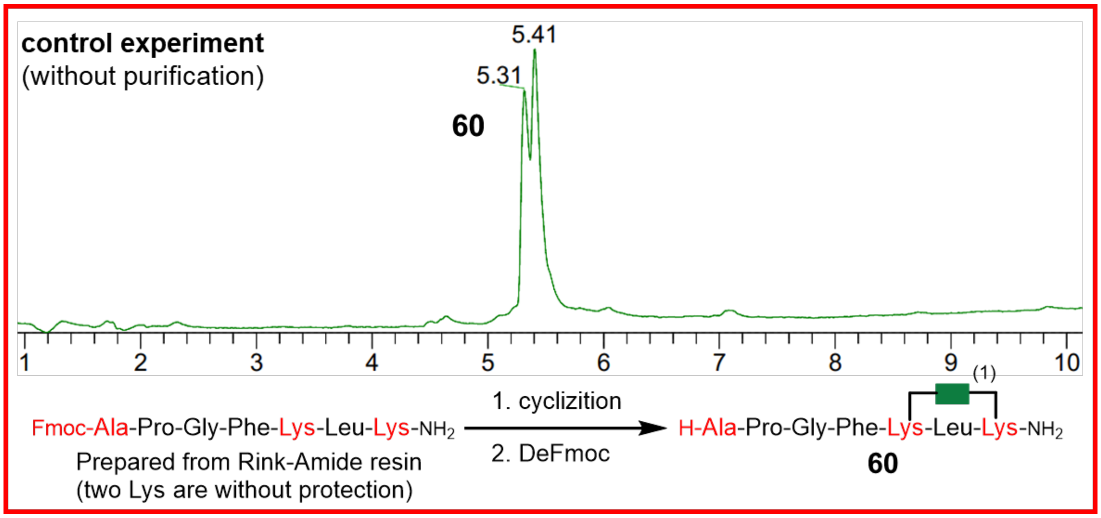

Supplementary Fig. 48. Control experiments. The structures of the side-to-side cyclization product **60** was confirmed by comparing LC of the samples prepared from N-terminus protected substrate.

Fmoc-Ala-Pro-Gly-Phe-Lys-Leu-Lys-NH_2 (TFA salt) was prepared from Rink-Amide resin bearing two unprotected Lys and protected N-terminal Ala. In the first step, cyclization via oPA should only go through the side-to-side fashion (Lys-Lys). Then, deprotection of the Fmoc gave the products which having the very similar peaks, retention times (5.31 min & 5.41 min) and identical molecular weight in comparison of **60**. Thereby, the structures of the major side-to-side stapled products can be confirmed.

The structure of next two products **58** and **59** were also determined by the same strategy.

2.2 Facile extension with electron deficient π electrophiles

Most of reactions were conducted at 0.01 mmol scale (except for **71**) from the corresponding linear peptide with multi-step in one pot, and the progress of reactions were monitored by UPLC-MS.

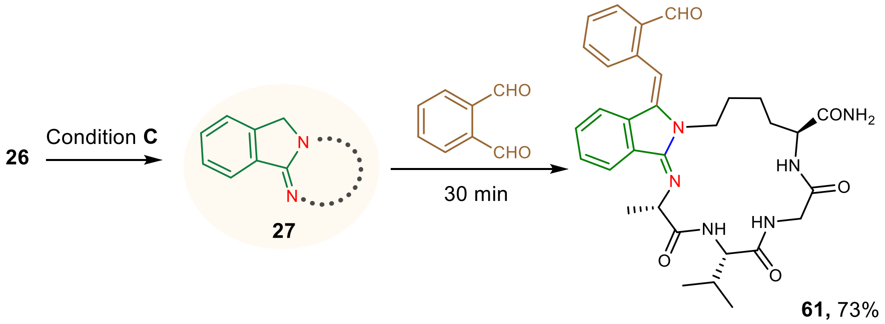

Cyclic peptide **27** which was prepared from linear peptide **26** under condition **C** was subsequently treated with 3.0 equiv of OPA in 30 min to give product **61** (HCOOH salt, 0.0073 mmol, 4.6 mg) in 73% yield over two steps in one pot.

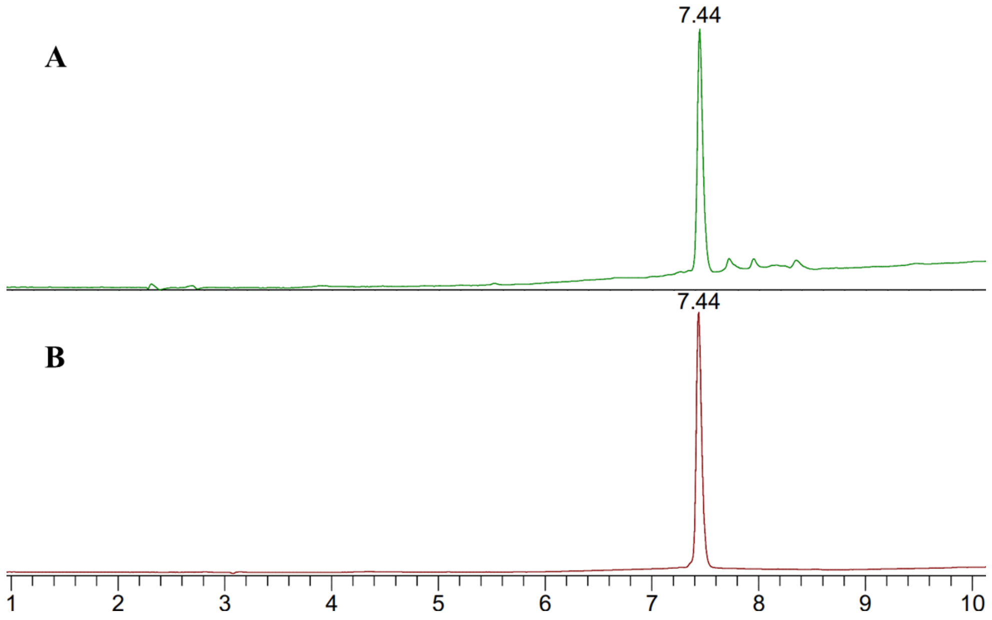

Supplementary Fig. 49. A) Crude UPLC trace of cyclization reaction. **B)** UPLC trace of purified product **61** (rt =7.44 min, λ = 254 nm).

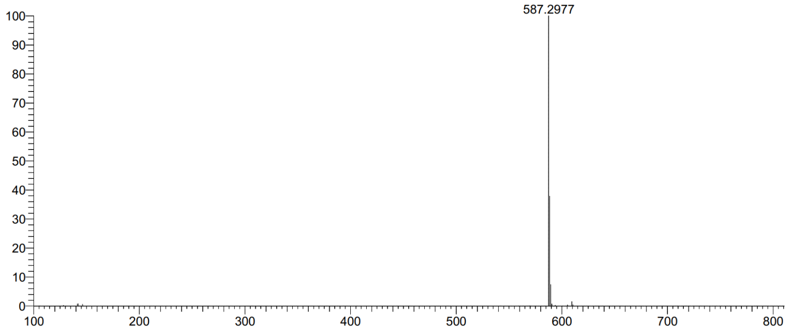

HRMS (ESI): calcd. for $C_{32}H_{39}N_6O_5$ $[M+H]^+$= 587.2976; found 587.2977.

Unsuccessful condensation reaction of 27 with other aldehydes

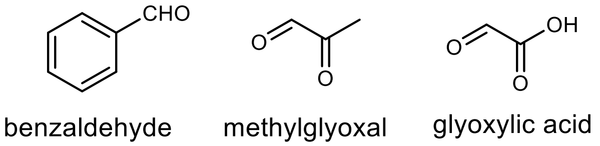

27 prepared from **26** under condition **C** was subsequently treated with 3.0 equiv of benzaldehyde, methylglyoxal or glyoxylic in one-pot fashion in 30 min respectively.

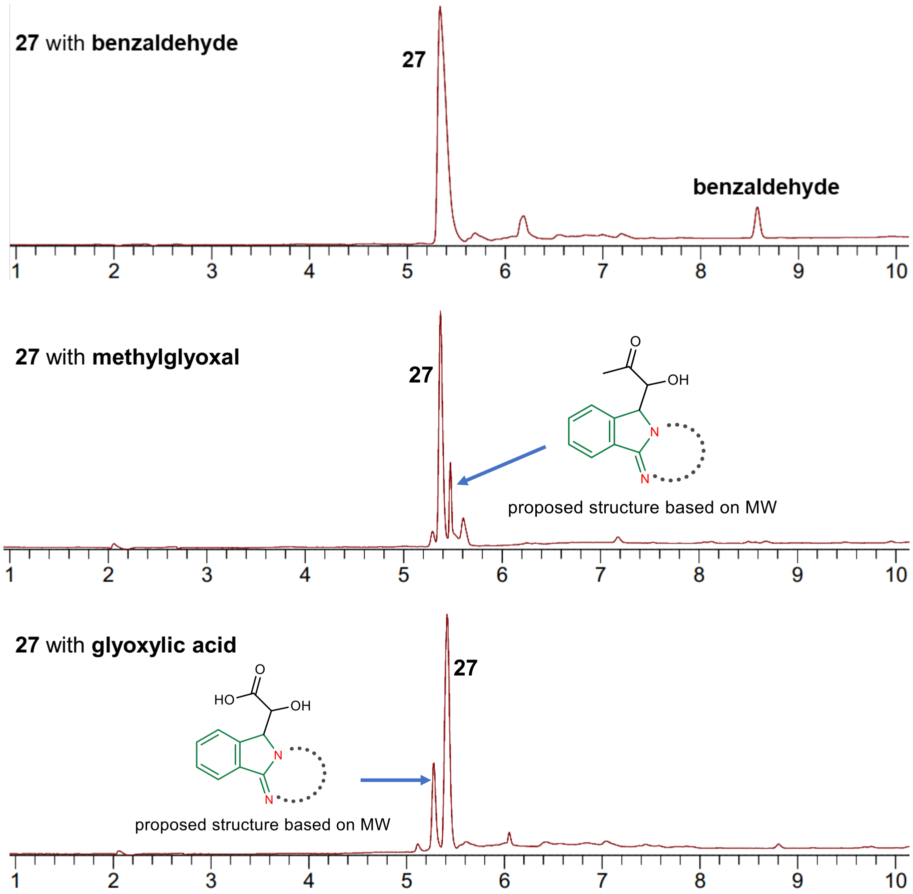

Supplementary Fig. 50. LC trace of further reactions of **27** with benzaldehyde, methylglyoxal, glyoxylic acid.

While a small amount of 1,2-addition product at **C3** was formed via the reaction of **27** with methylglyoxal and glyoxylic acid, there were little **61**-like condensation product.

Reaction of model substrate 68 with 62

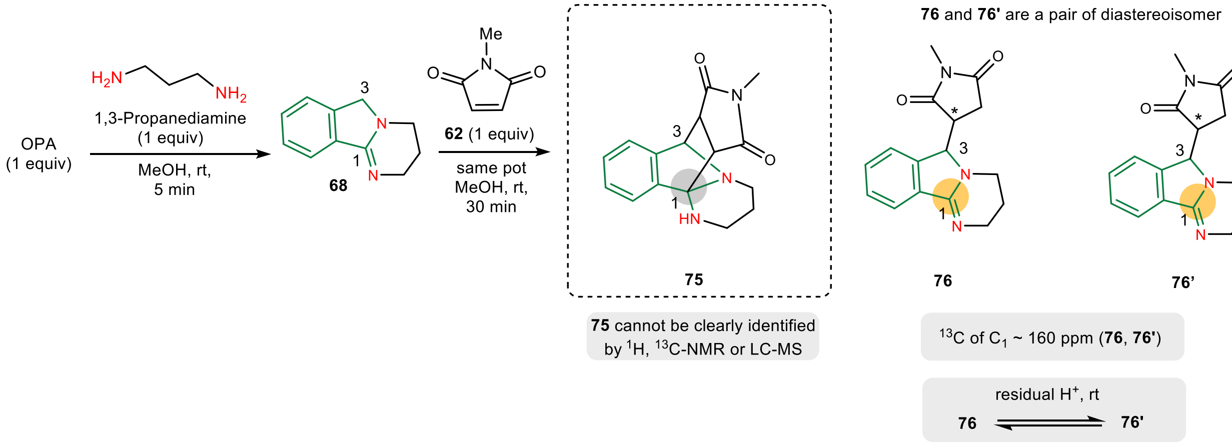

Supplementary Fig. 51. Reaction of model substrate **68** with **62**.

To a clear solution of 1,3-propanediamine (1.0 equiv, 0.05 mmol) in 1.0 mL of MeOH, OPA (1.0 equiv, 0.05 mmol) was added and stirred at room temperature for 5 min. LC-MS analysis indicated that **68** was formed cleanly. Reagent **62** (1.0 equiv) was then added, the resulting mixture was stirred for another 30 min. The reaction mixture was analyzed by LC-MS. The final product was obtained as a mixture of two regioisomers **76** and **76'**. The two isomers are difficult to be fully separated by HPLC. Only one isomer can be obtained in high purity (retention time: 5.15 min) by semi-prep HPLC. A mixture of the two isomers can be obtained by preparative normal phase silica gel TLC. The NMR analysis of the reaction mixture in Methanol-D4 did not clearly identify the cycloadduct **75** in our hands.

Compound **68**: **^{1}H NMR (400 MHz, Acetic Acid-*d*$_4$)** δ 8.05 (d, *J* = 7.8 Hz, 1H), 7.74 (t, *J* = 7.4 Hz, 1H), 7.68 (d, *J* = 7.6 Hz, 1H), 7.62 (t, *J* = 7.4 Hz, 1H), 4.87 (s, 2H), 3.85 (t, *J* = 5.8 Hz, 2H), 3.67 (t, *J* = 5.8 Hz, 2H), 2.23 (p, *J* = 5.8 Hz, 2H). **^{13}C NMR (101 MHz, Acetic)** δ 158.86, 141.79, 133.35, 128.70, 127.77, 123.27, 122.88, 56.69, 42.38, 39.44, 38.05.

Compound **76 (one of the isomers)**: **^{1}H NMR (400 MHz, Acetic Acid-*d*$_4$)** δ 8.14 (d, *J* = 6.8 Hz, 1H), 7.76–7.71 (m, 1H), 7.68 (t, *J* = 7.4 Hz, 1H), 7.31 (d, *J* = 7.4 Hz, 1H), 5.62 (d, *J* = 4.2 Hz, 1H), 4.09 (m, 1H), 3.90 (m, 1H), 3.84–3.74 (m, 2H), 3.72–3.64 (m, 1H), 3.04 (s, 3H), 2.79 (dd, *J* = 18.4, 9.0 Hz, 1H), 2.27 (m, 2H), 1.68 (dd, *J* = 18.4, 4.6 Hz, 1H). **^{13}C NMR (101 MHz, Acetic)** δ 177.37, 176.42, 158.75, 141.08, 134.12, 130.14, 128.37, 123.92, 122.66, 65.93, 40.53, 40.33, 38.33, 27.96, 24.50, 24.36.

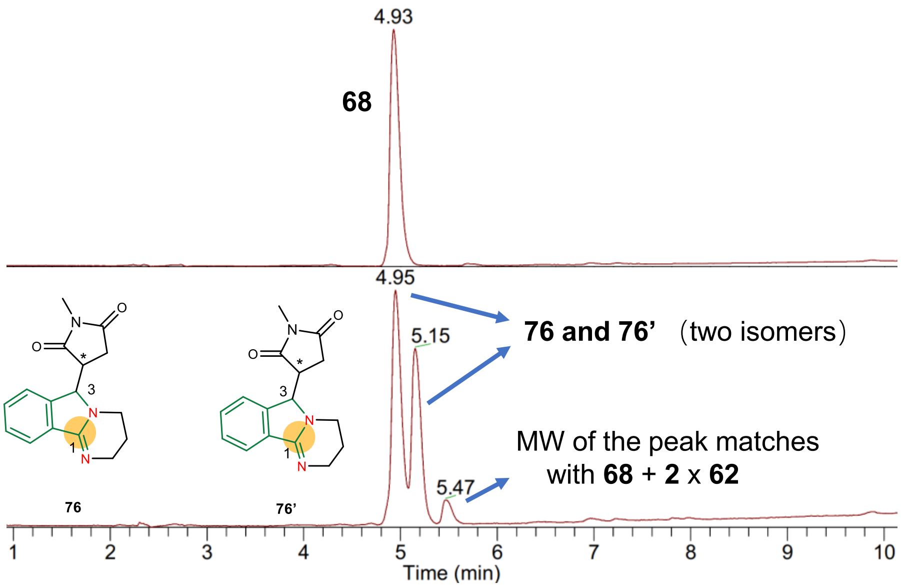

Supplementary Fig. 52. LC trace of the reaction of **68** and **62** (**76** overlaps with **68**).

LC-MS analysis showed that reaction of **68** with **62** readily gave products **76** and **76'** as a pair of diastereoisomers in MeOH at rt. A minor side product was also observed. LC-MS analysis cannot tell whether cycloadduct intermediate **75** was formed.

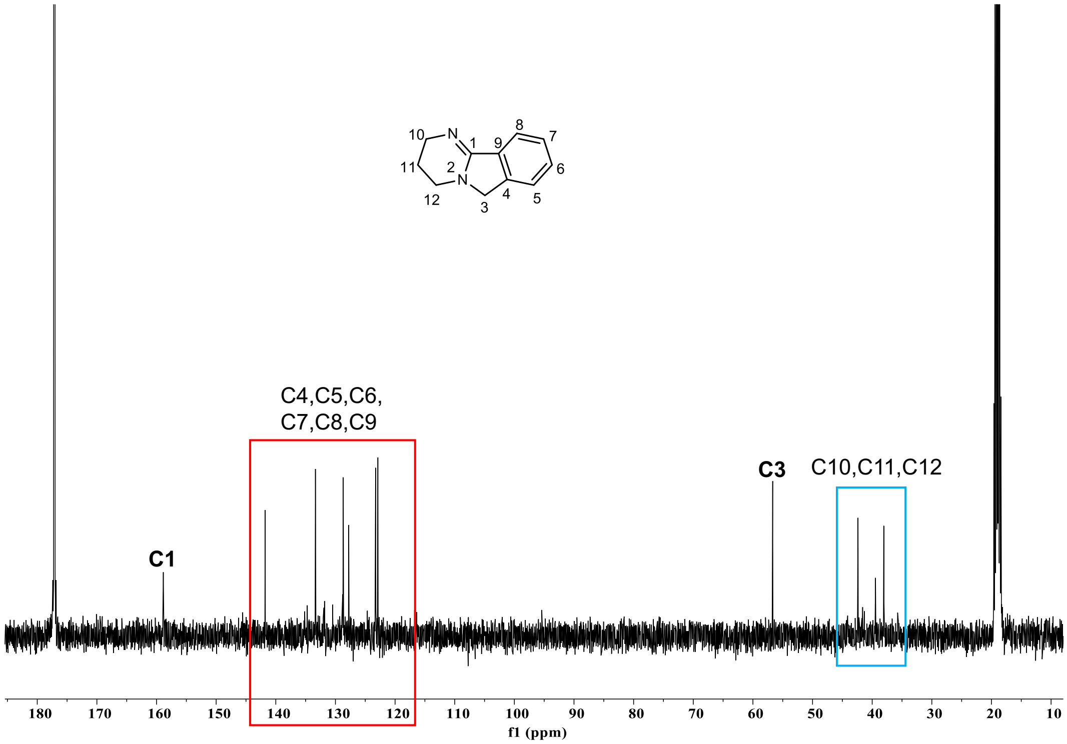

Supplementary Fig. 53. ^{13}C NMR spectrum of compound **68**.

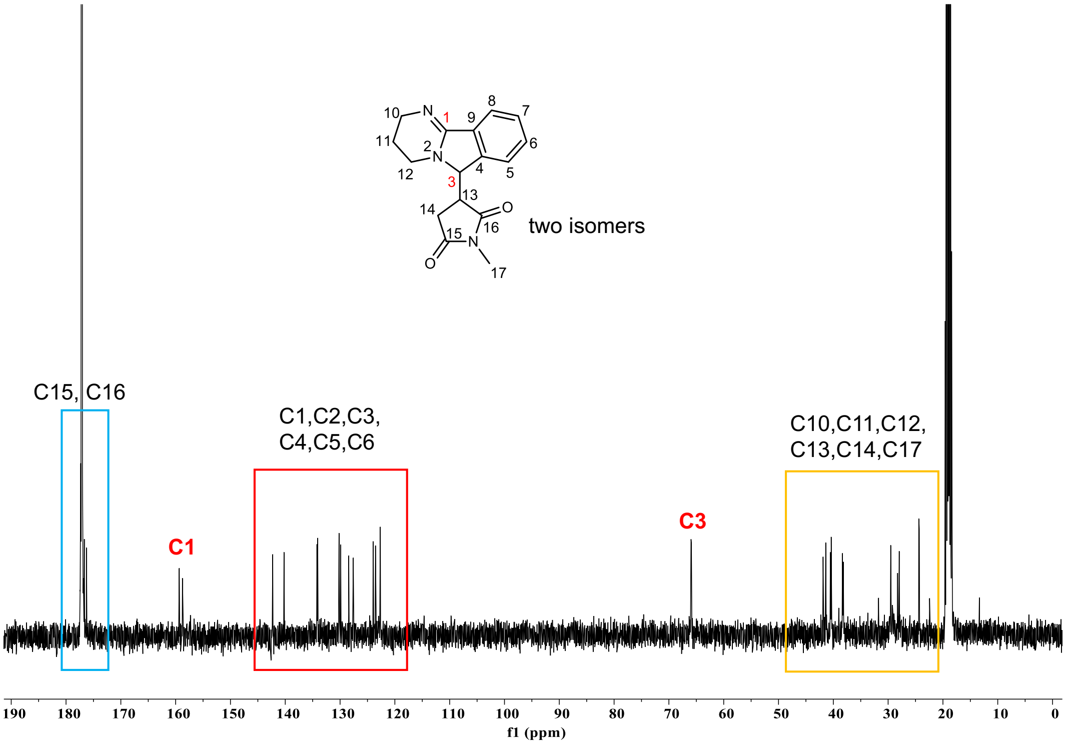

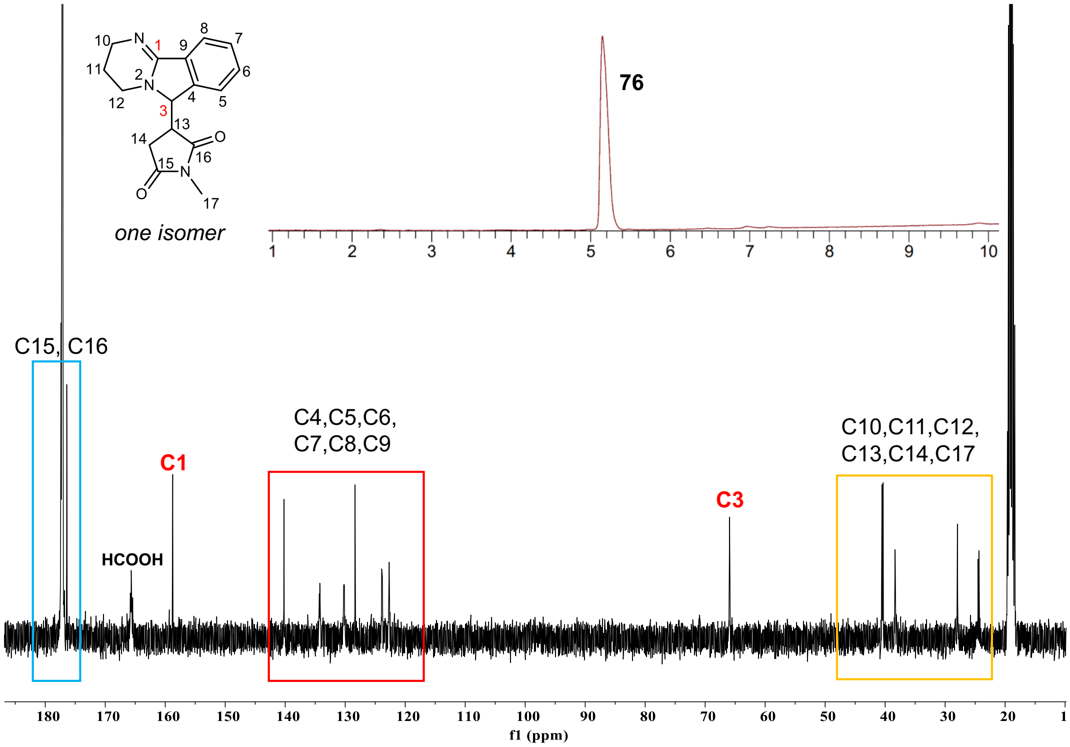

Supplementary Fig. 54. ^{13}C NMR spectra of compound **76**.

Based on the analysis of ^{13}C NMR spectra, compound **76** matches with the C3-substituted product. (The chemical shift of its C1 is around 160 ppm vs 95 ppm of C1 in **75** based on the prediction of Chemdraw).

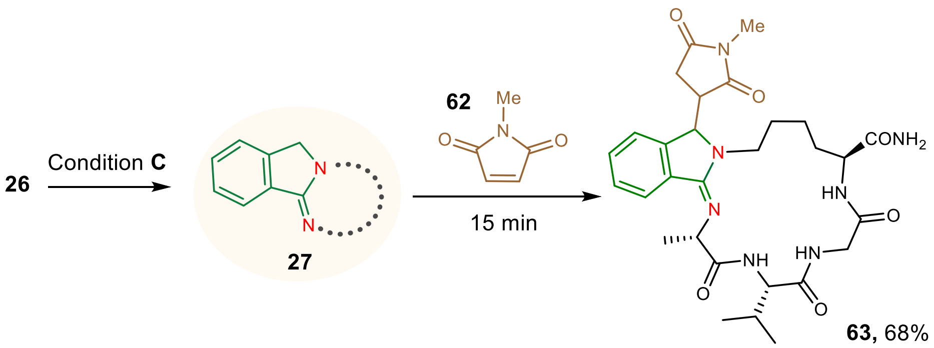

Cyclic peptide **27** which was prepared from linear peptide **26** under condition **C** was subsequently treated with 3.0 equiv of **62** in 15 min to give product **63** (HCOOH salt, 0.0068 mmol, 4.3 mg) in 68% yield over two steps in one pot.

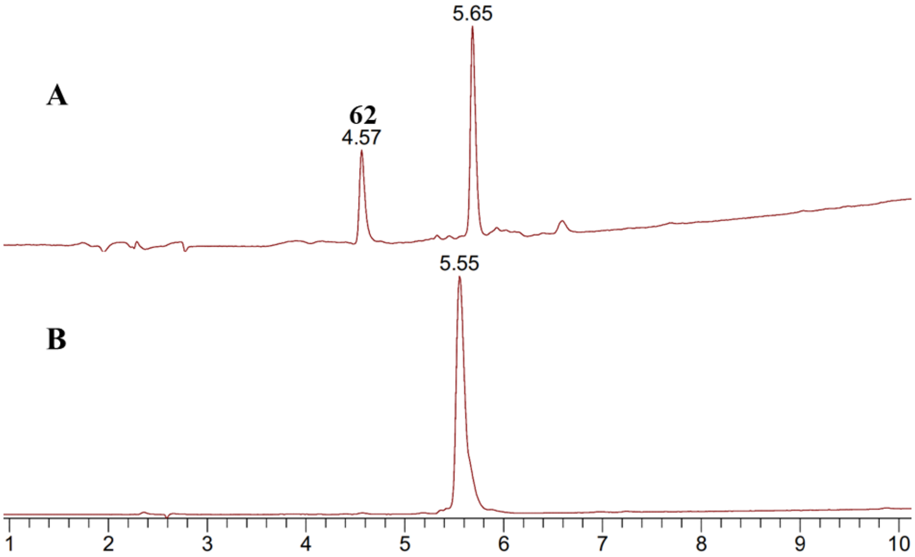

Supplementary Fig. 55. A) Crude UPLC trace of cyclization reaction. **B)** UPLC trace of purified **63** (rt =5.55 min, λ = 254 nm).

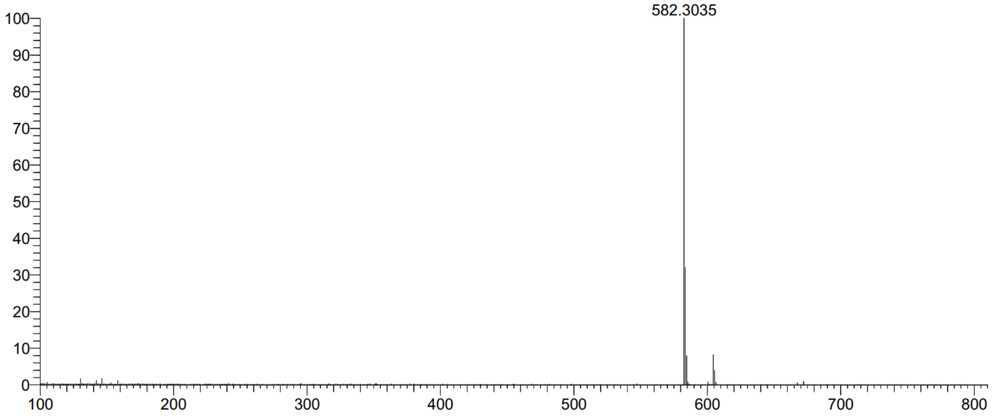

HRMS (ESI): calcd. for $C_{29}H_{40}N_7O_6$ $[M+H]^+$= 582.3035; found 582.3035.

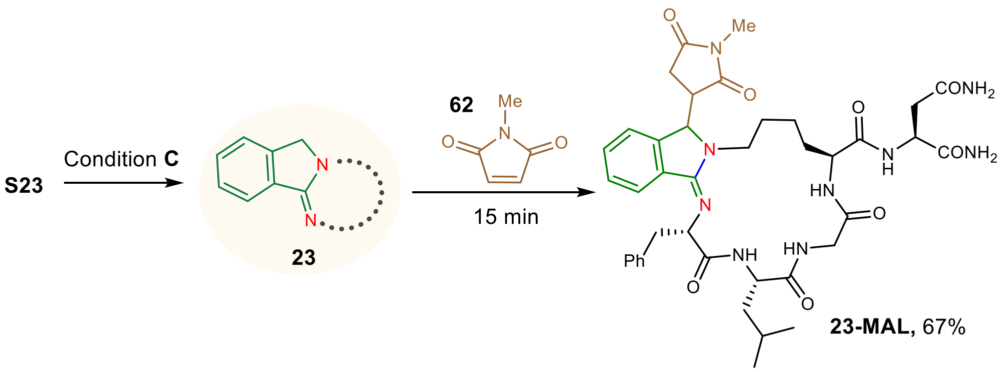

Cyclic peptide **23** which was prepared from linear peptide **S23** under condition **C** was subsequently treated with 3.0 equiv of **62** in 15 min to give product **23-MAL** (HCOOH salt, 0.0067 mmol, 5.5 mg, inseparable isomers) in 67% yield over two steps in one pot.

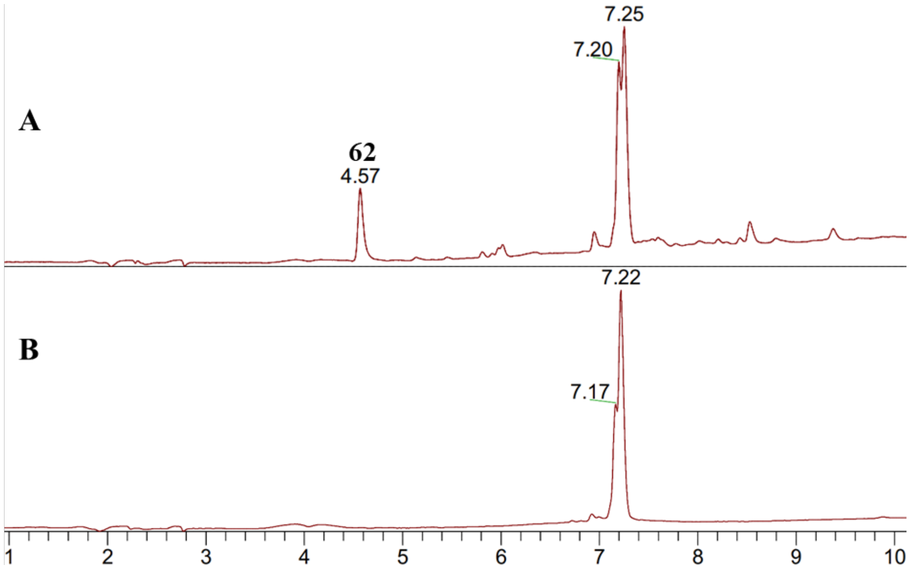

Supplementary Fig. 56. A) Crude UPLC trace of cyclization reaction. **B)** UPLC trace of purified product **23-MAL** (rt =7.17 & 7.22 min, λ = 254 nm).

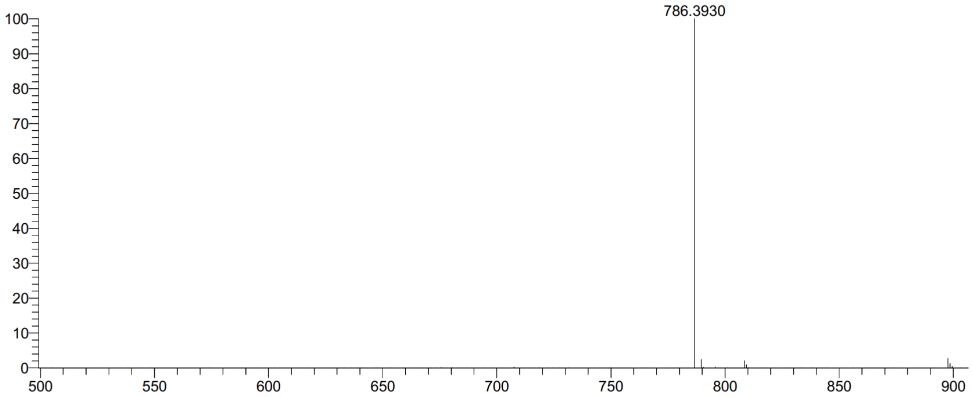

HRMS (ESI): calcd. for $C_{40}H_{52}N_9O_8$ $[M+H]^+$= 786.3933; found 786.3930.

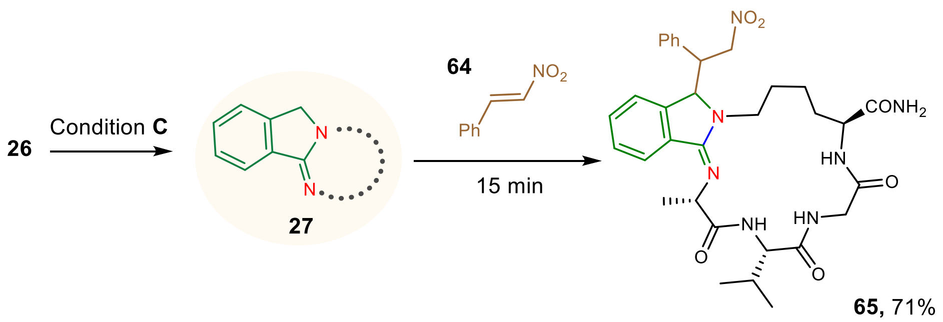

Cyclic peptide **27** which was prepared from linear peptide **26** under condition **C** was subsequently treated with 3.0 equiv of **64** in 15 min to give product **65** (HCOOH salt, 0.0071 mmol, 4.7 mg) in 71% yield over two steps in one pot.

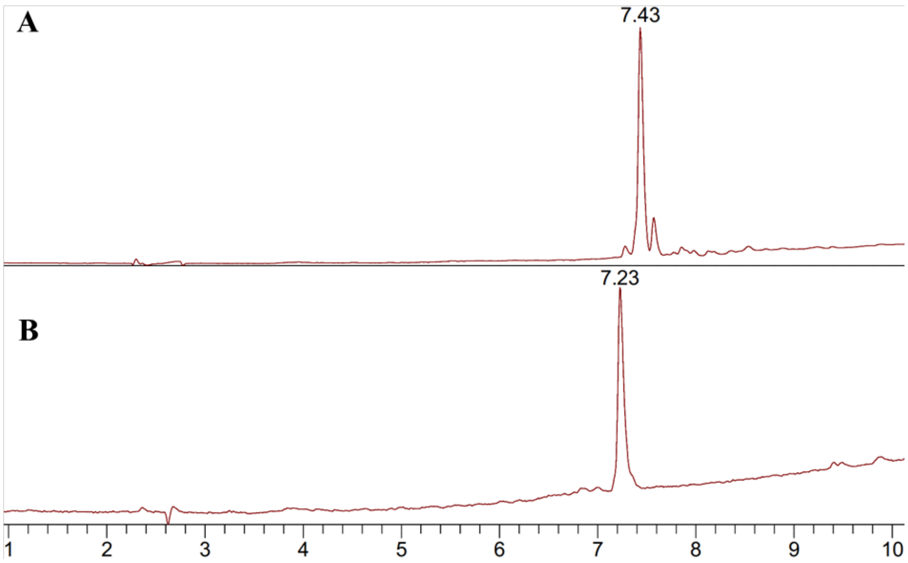

Supplementary Fig. 57. A) Crude UPLC trace of cyclization reaction. **B)** UPLC trace of purified product **65** (rt =7.23 min, λ = 254 nm).

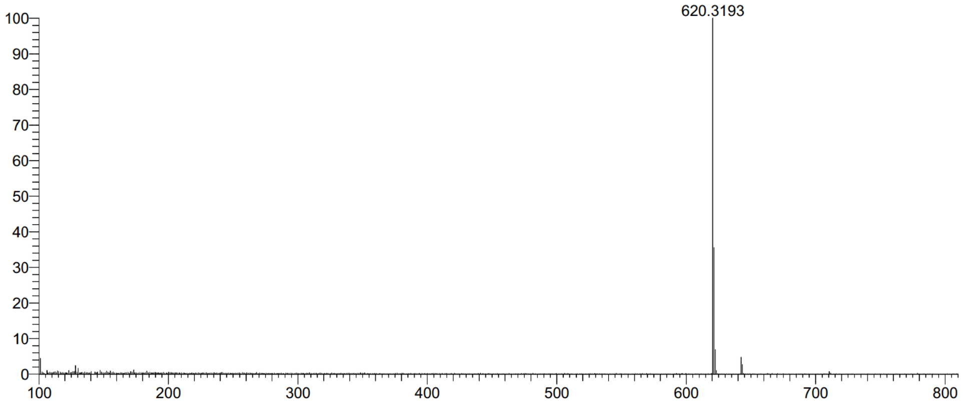

HRMS (ESI): calcd. for $C_{32}H_{42}N_7O_6$ $[M+H]^+$ = 620.3191; found 620.3193.

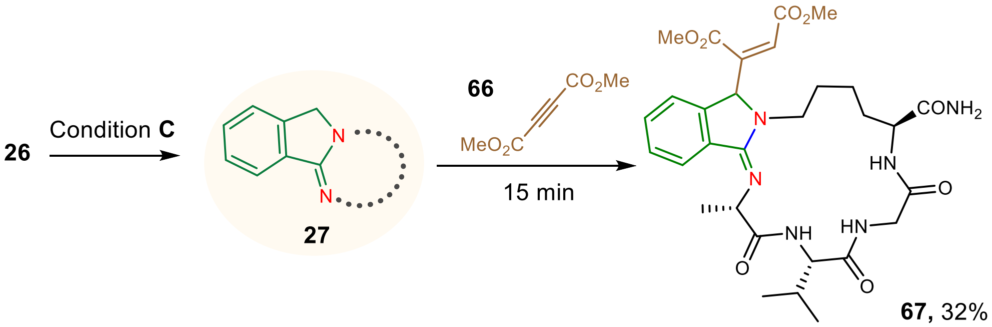

Cyclic peptide **27** which was prepared from linear peptide **26** under condition **C** was subsequently treated with 5.0 equiv of **66** in 1 h to give product **67** (HCOOH salt, 0.0032 mmol, 2.1 mg) in 32% yield over two steps in one pot.

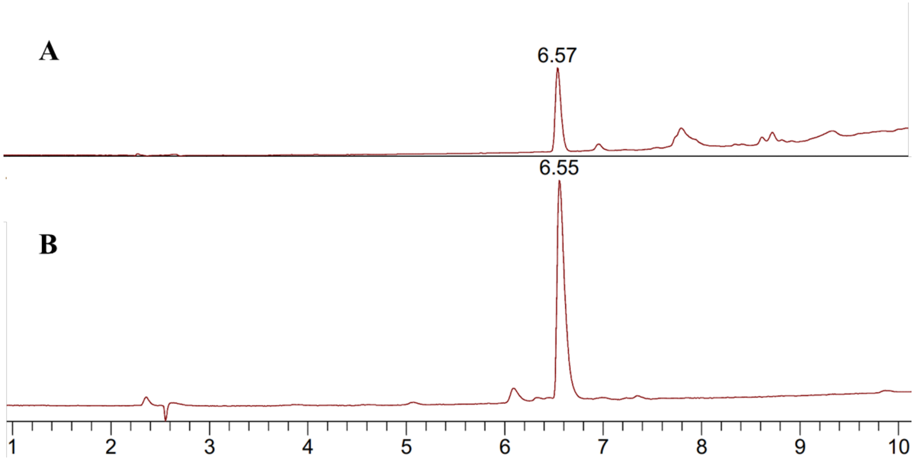

Supplementary Fig. 58. A) Crude UPLC trace of cyclization reaction. **B)** UPLC trace of purified **67** (rt =6.55 min, λ = 254 nm).

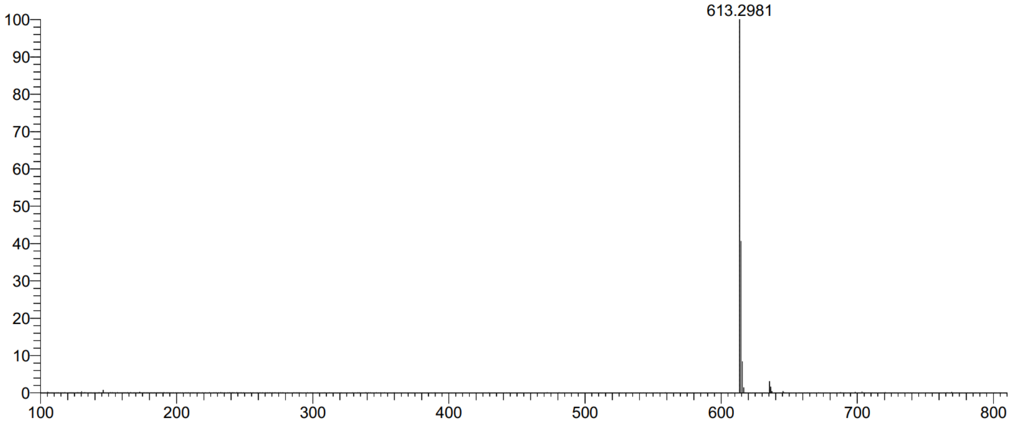

HRMS (ESI): calcd. for $C_{30}H_{41}N_6O_8$ $[M+H]^+$= 613.2980; found 613.2981.

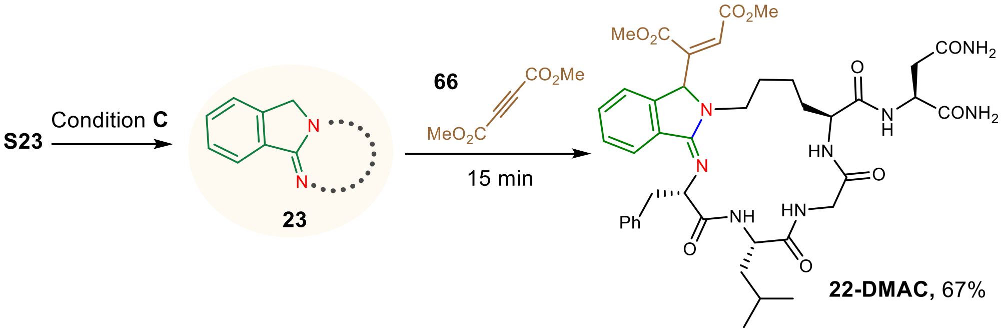

Cyclic peptide **23** which was prepared from linear peptide **S23** under condition **C** was subsequently treated with 5.0 equiv of **66** in 1 h to give product **23-DMAC** (HCOOH salt, 0.0067 mmol, 5.8 mg) in 67% yield over two steps in one pot.

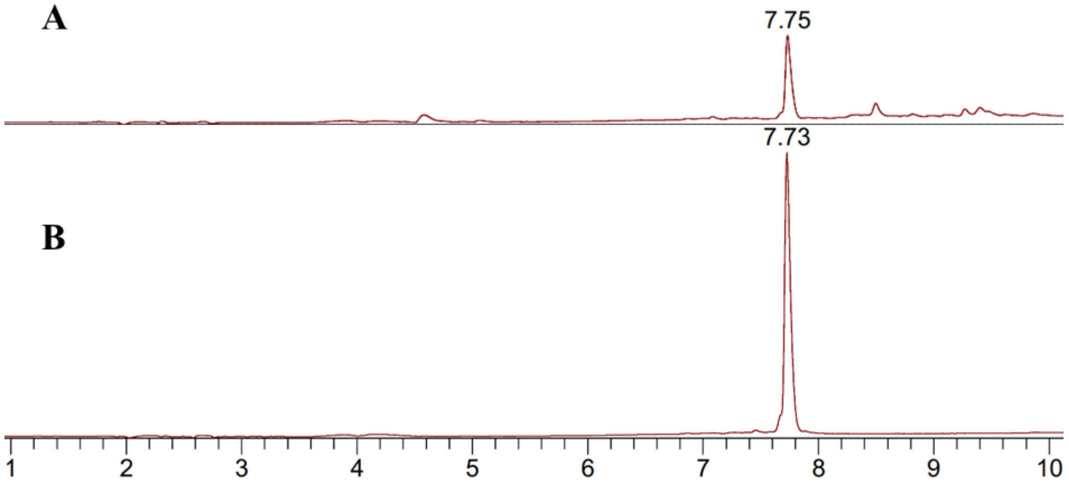

Supplementary Fig. 59. A) Crude UPLC trace of cyclization reaction. **B)** UPLC trace of purified product **23-DMAC** (rt =7.73 min, λ = 254 nm).

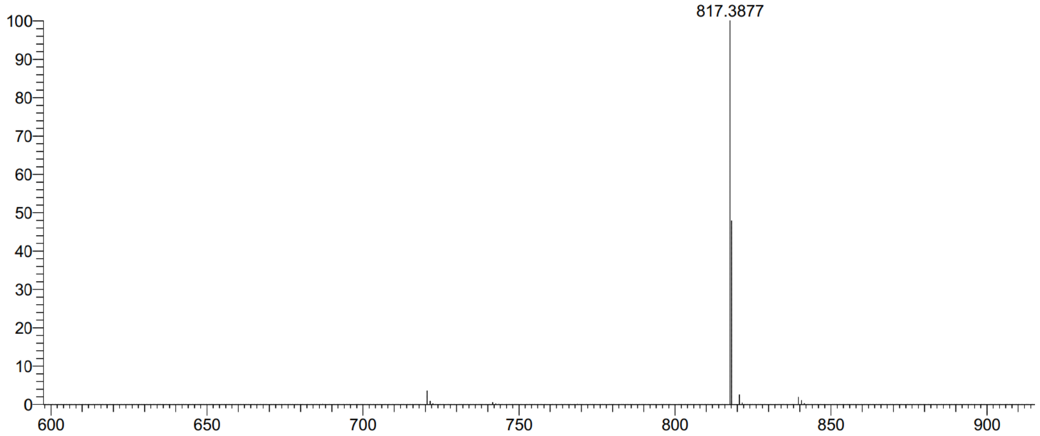

HRMS (ESI): calcd. for $C_{41}H_{53}N_8O_{10}$ $[M+H]^+$= 817.3879; found 817.3877.

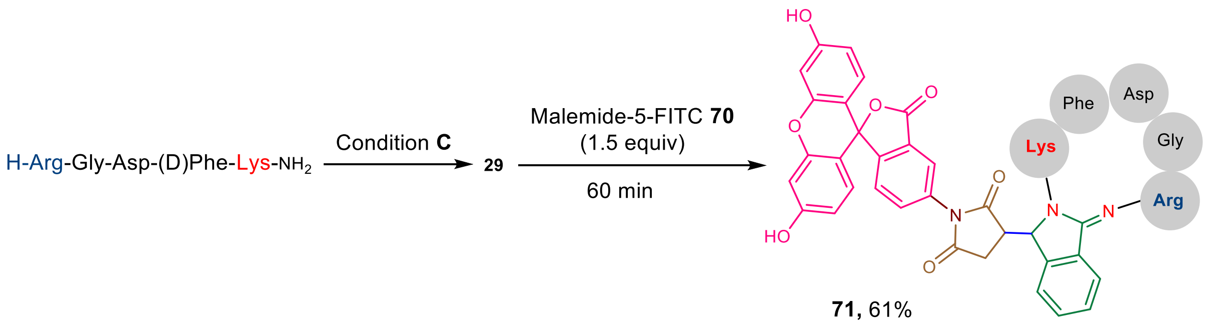

Cyclic peptide **29** which was prepared from linear peptide **S29** under condition **C** was subsequently treated with 1.5 equiv of **70** in 60 min to give product **71** (HCOOH salt, 0.0061 mmol, 7.5 mg, inseparable isomers) in 61% yield over two steps in one pot. (The reaction was conducted at 0.005 mmol scale). (see **Figure 5c**. for the detailed course of reaction).

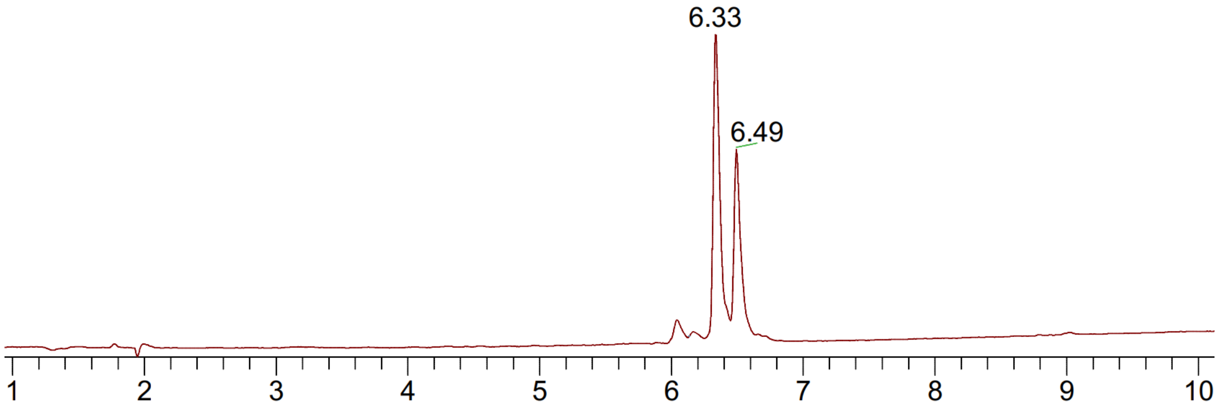

Supplementary Fig. 60. UPLC trace of purified product **71** (rt =6.33 & 6.49 min, λ = 254 nm).

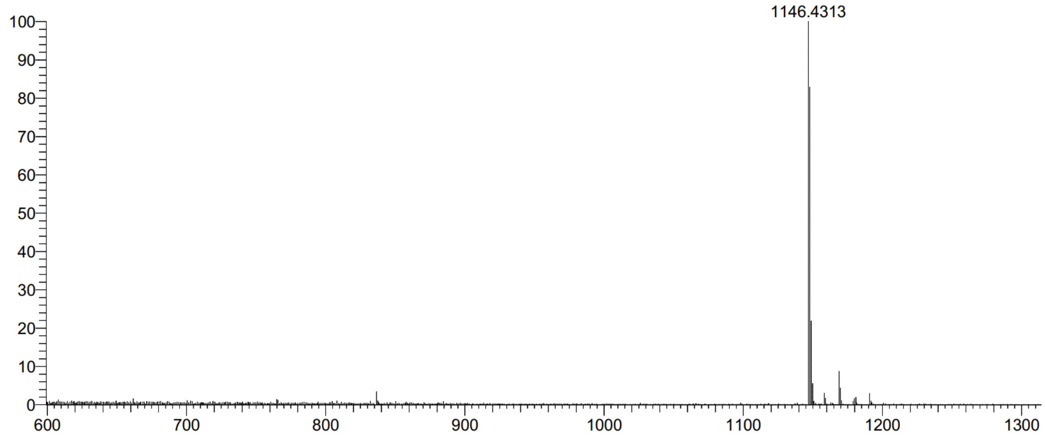

HRMS (ESI): calcd. for $C_{59}H_{60}N_{11}O_{14}$ $[M+H]^+$= 1146.4316; found 1146.4313.

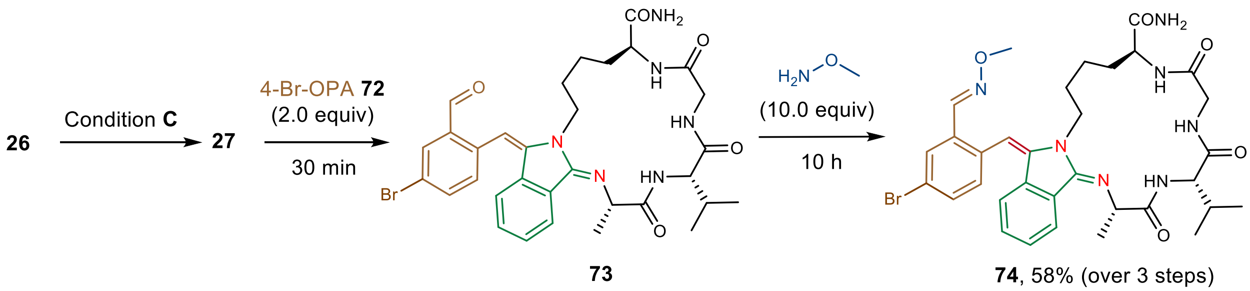

Cyclic peptide **27** which was prepared from linear peptide **26** under condition **C** was sequentially treated with 4-Br-OPA **72** (2.0 equiv, 30 min, rt) and methoxyamine (10.0 equiv, 10 h, rt) to give product **74** (HCOOH salt, 0.0058 mmol, 4.3 mg) in 58% yield over three steps in one pot.

(see **Figure 5d**. for the detailed course of reaction).

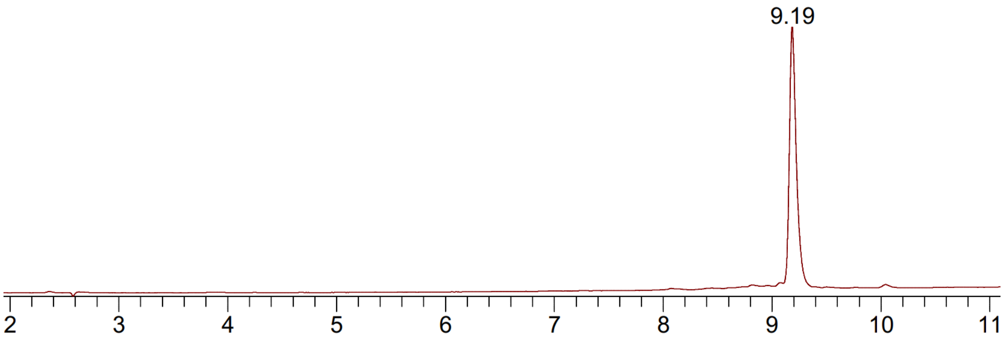

Supplementary Fig. 61. UPLC trace of purified product **74** (rt =9.19 min, λ = 254 nm).

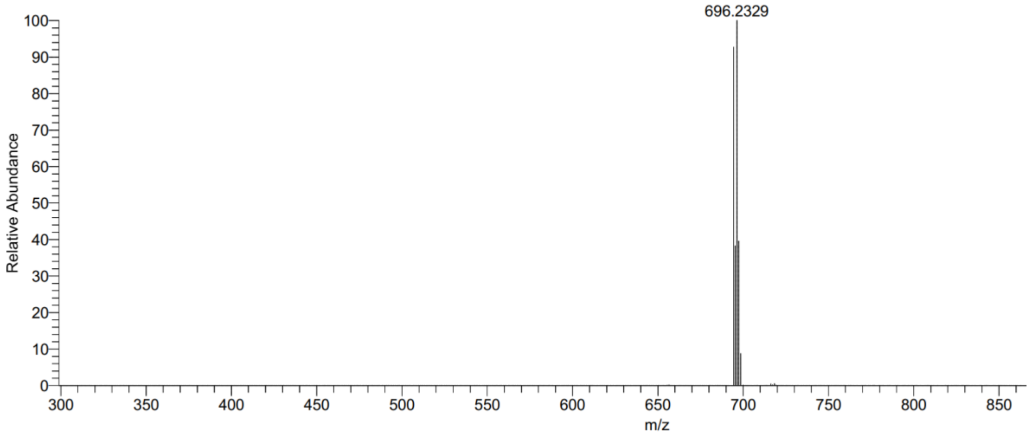

HRMS (ESI): calcd. for $C_{33}H_{41}BrN_7O_5$ $[M+H]^+$= 694.2347; found 696.2329.

Unsuccessful further reaction of 27 with electron-deficient electrophiles

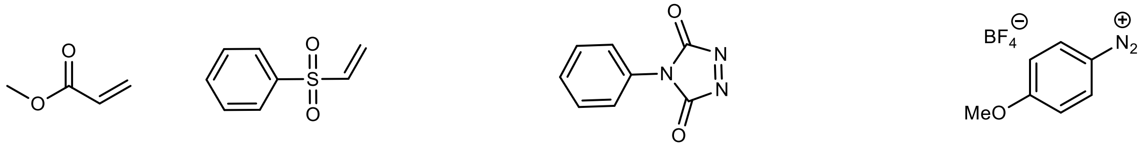

methyl acrylate   vinyl sulfones   4-phenyl-3H-1,2,4-triazoline-3,5(4H)-diones   aryl diazonium salts

27 prepared from **26** under condition **C** was subsequently treated with 3.0 equiv of methyl acrylate, vinyl sulfones, 4-phenyl-3H-1,2,4-triazoline-3,5(4H)-diones and aryl diazonium salts in one-pot fashion in 30 min respectively.

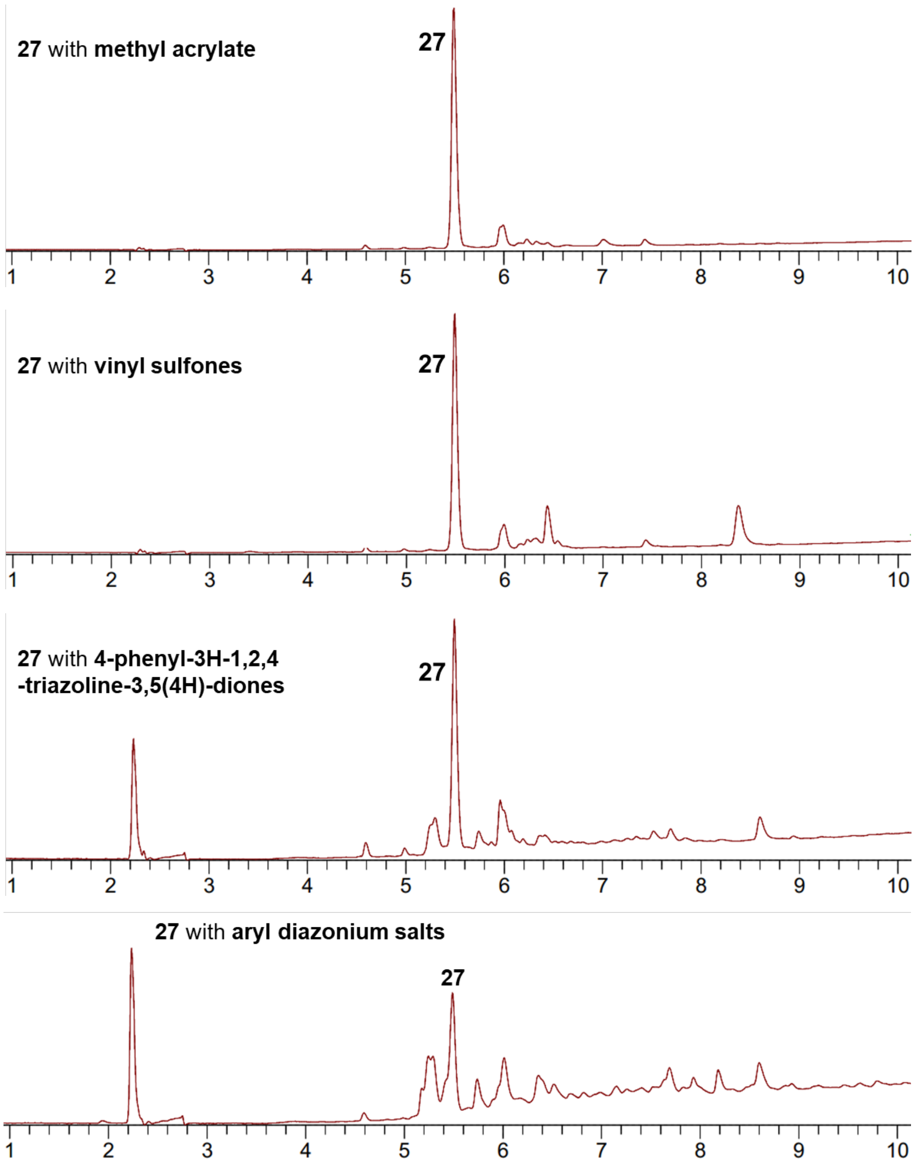

Supplementary Fig. 62. LC trace of further reactions of **27** with electron-deficient electrophiles.

2.3 Evaluation of organic cosolvents, concentration and pH

i) The effect of organic cosolvents

Except for alcohol co-solvents of MeOH and TFE, we also tested other organic co-solvents for the cyclization. Reaction with EtOH as co-solvents was still effective for promoting the macrocyclization, but other co-solvents could make the reaction complicated in varying degrees.

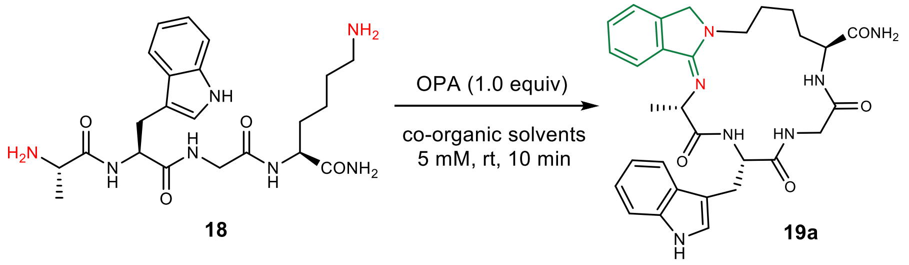

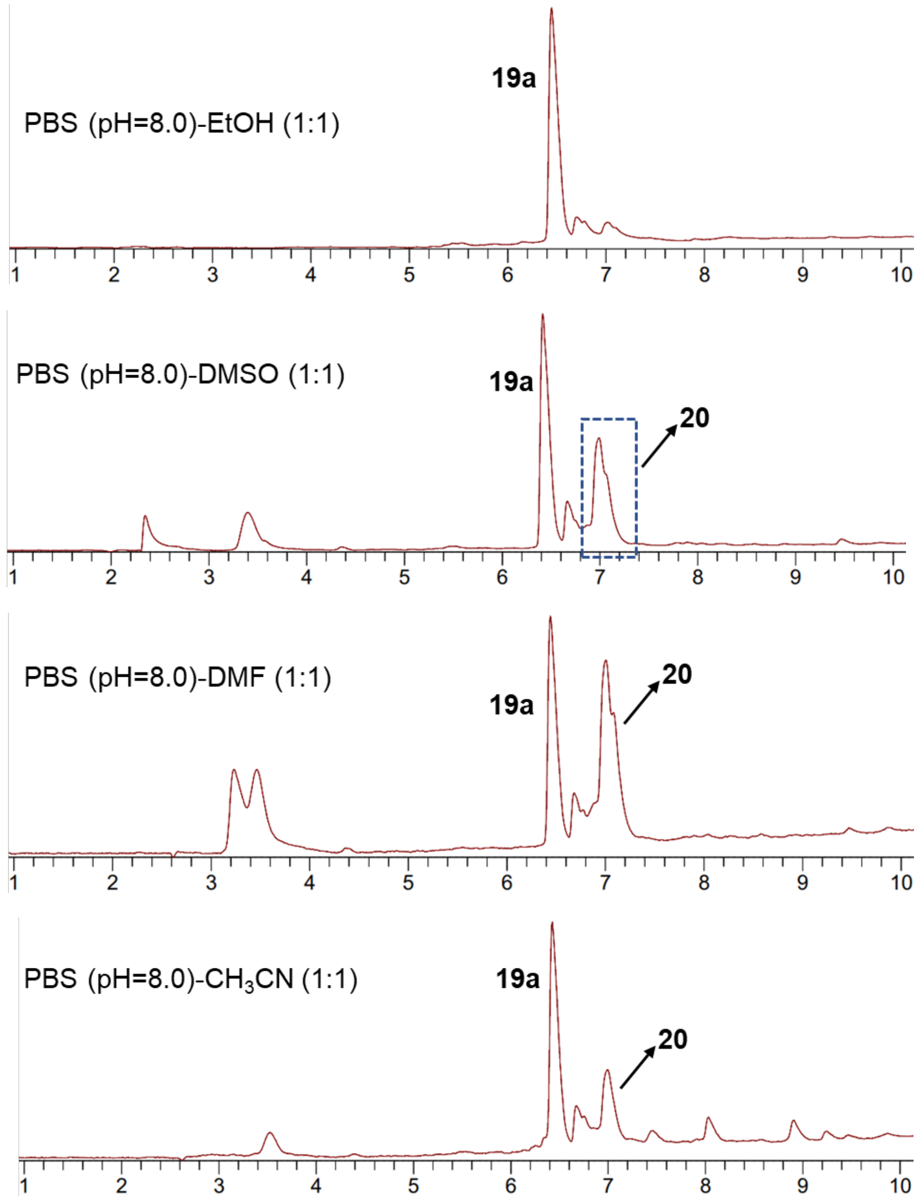

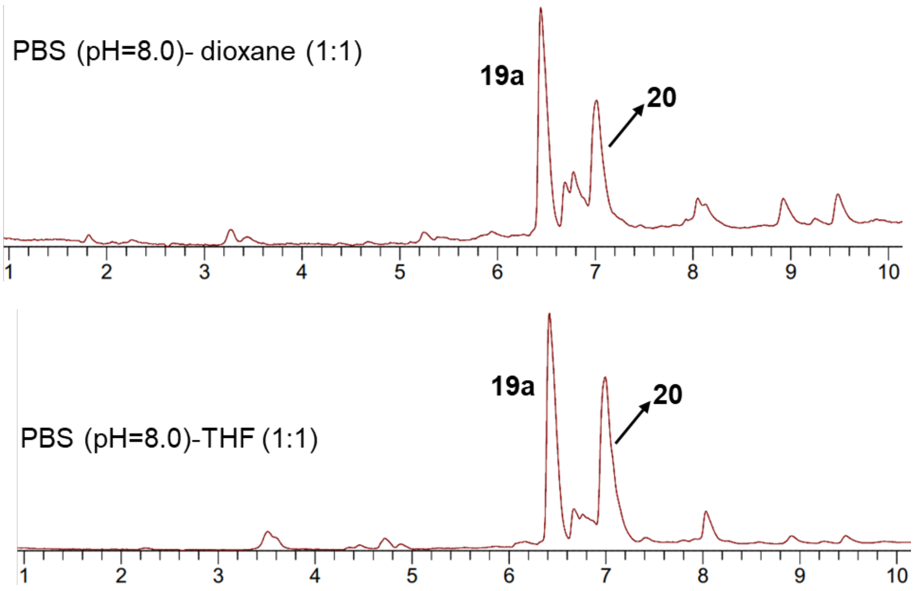

Supplementary Fig. 63. LC traces of cyclization reaction of compound **18** in selected co-solvents.

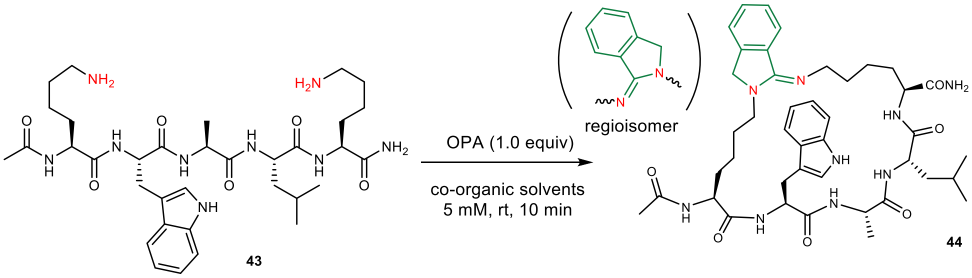

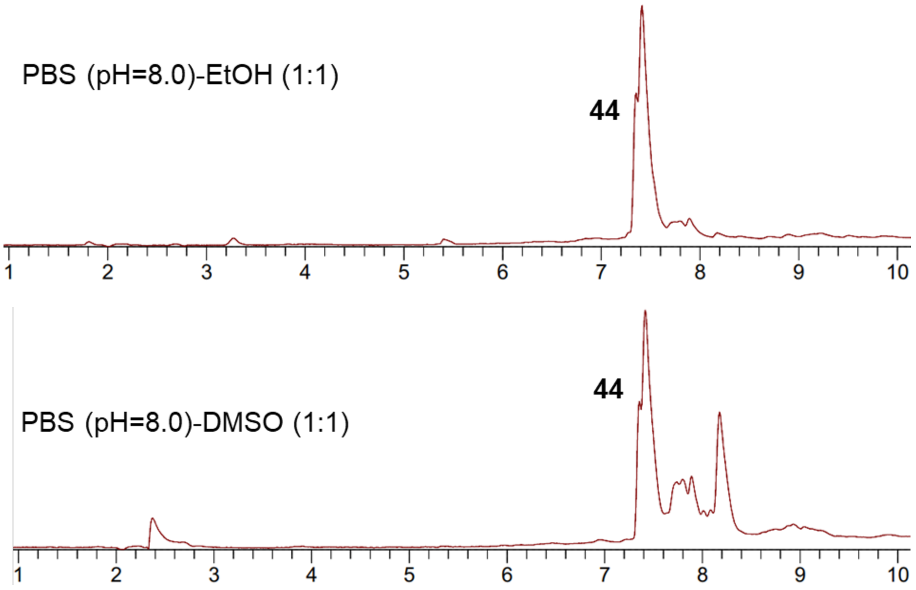

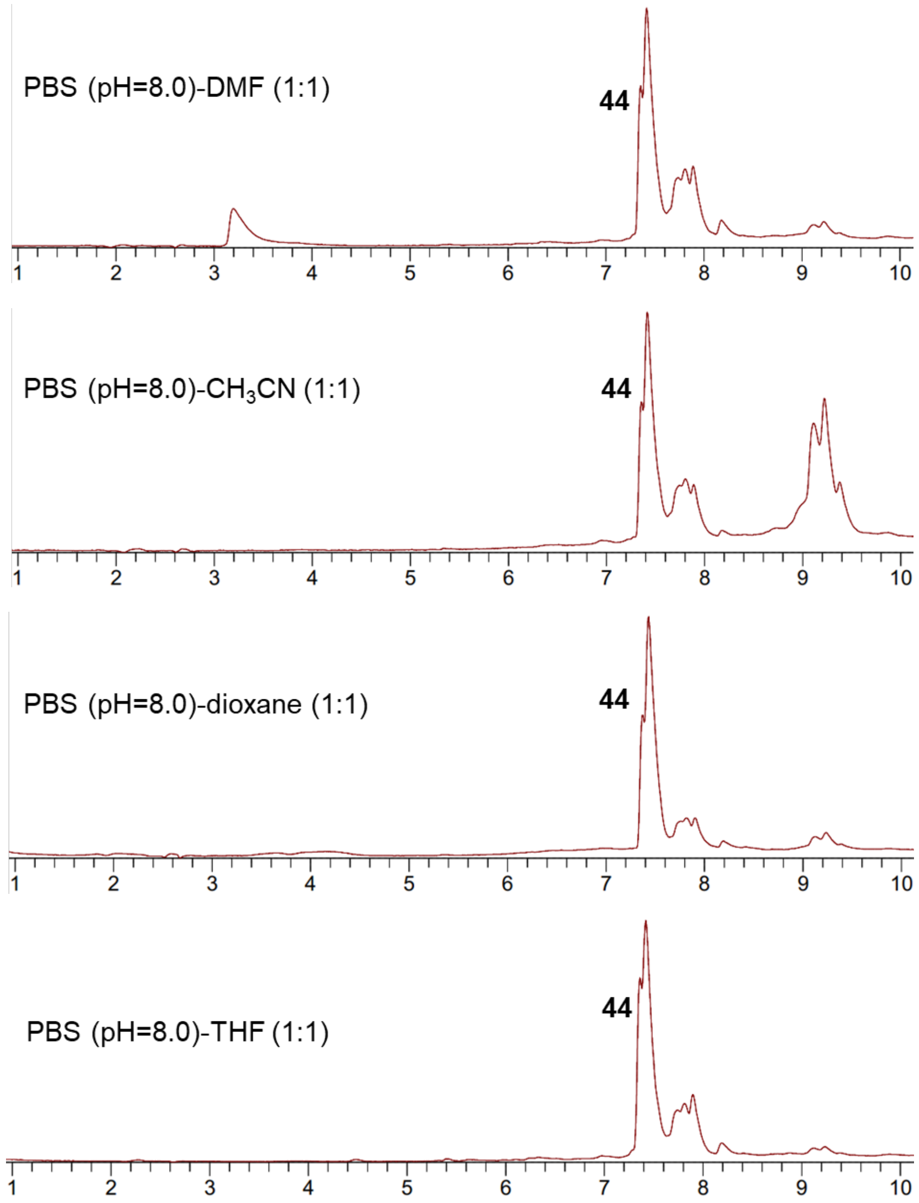

Supplementary Fig. 64. LC traces of cyclization reaction of compound **43** in selected co-solvents.

Based on the above results, we proposed that alcohol solvents like MeOH, TFE and EtOH could play important roles in dehydration to form the isoindolinimine linkage.

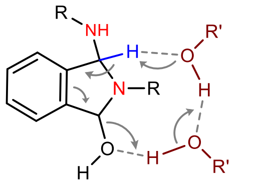

transition state for dehydration

* Number of R'OH can vary
* H_2O could be part of H-bonded network

Supplementary Fig. 65. Proposed role of alcohol solvent.

ii) The effect of reaction concentration

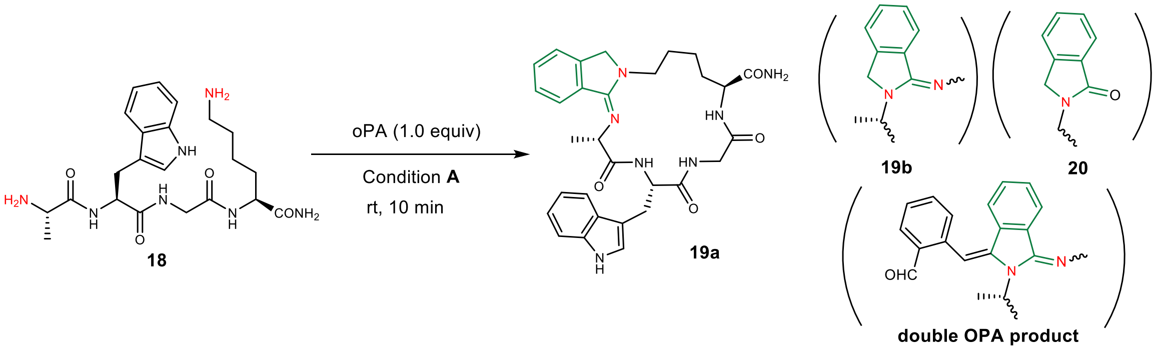

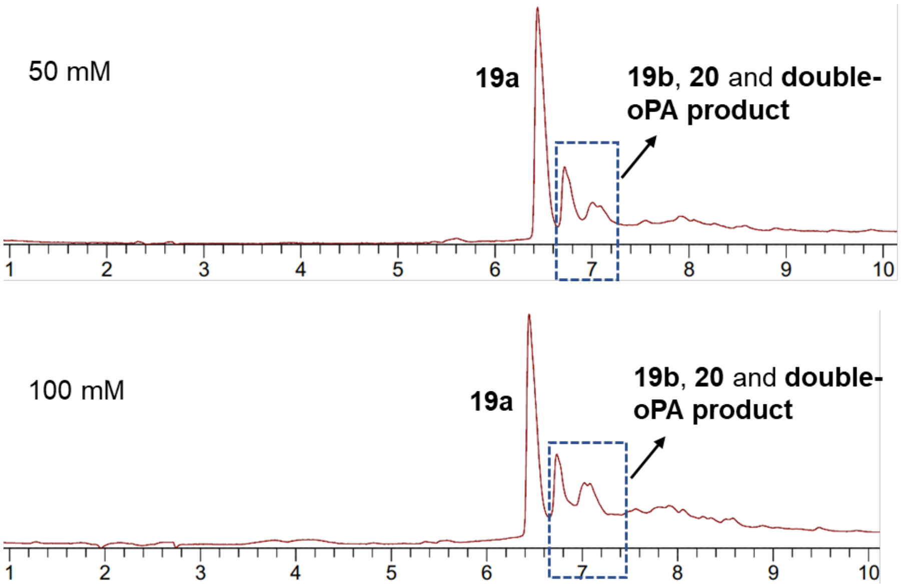

Supplementary Fig. 66. LC trace of compound **18** at concentrations of 50 mM and 100 mM. Cyclization of **18** at 50 mM and 100 mM concentration were monitored at 10 min via LC-MS analysis. The amount of byproducts (**20** and **double-oPA product**) were increased when the reaction was carried out at 50 mM. When the concentration was increased to 50 mM and 100 mM, the reaction still gave little **dimmer** product.

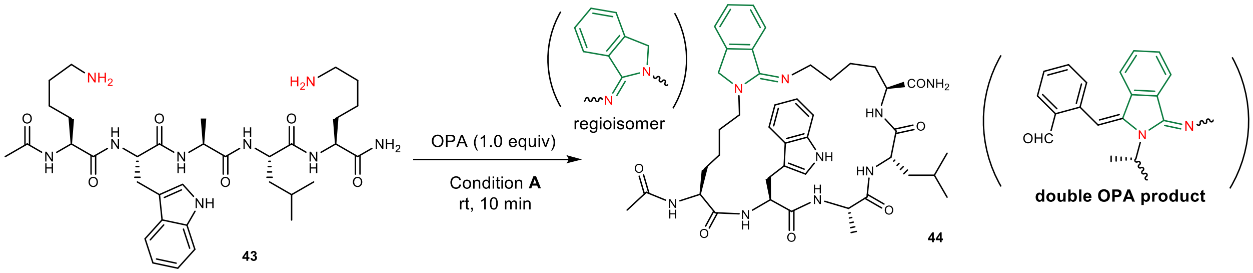

The **double OPA product** was formed when the reaction was performed at 100 mM. Moreover, **little intermolecular dimerization products** were formed for stapling of **43** even if the reaction concentration was increased to 100 mM.

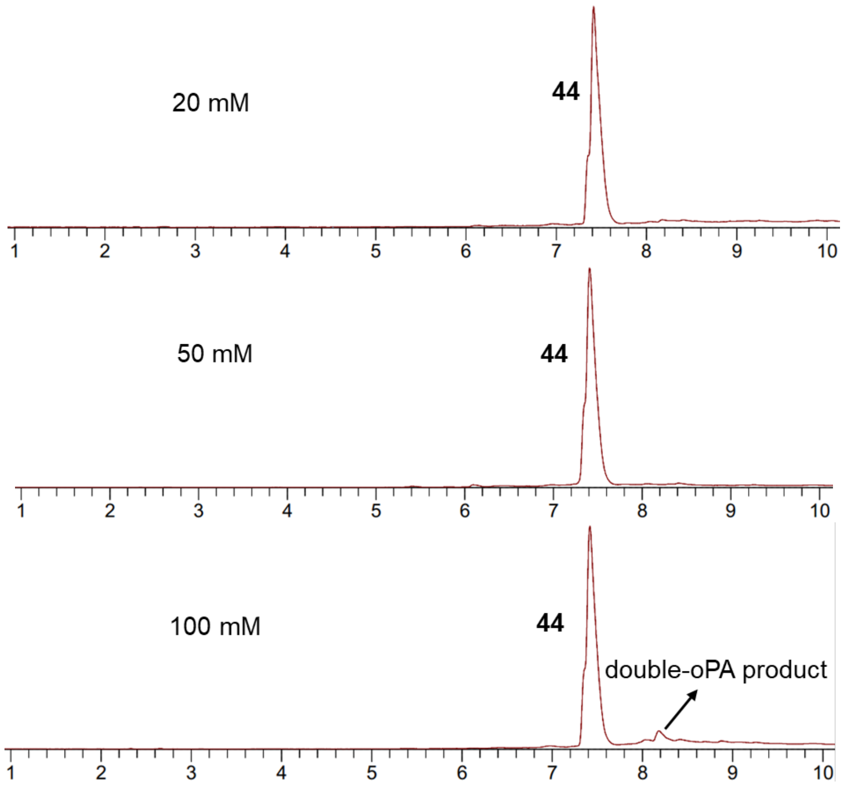

Supplementary Fig. 67. LC traces of compound **43** at concentration of 20 mM, 50 mM and 100 mM.

iii) The effect of pH

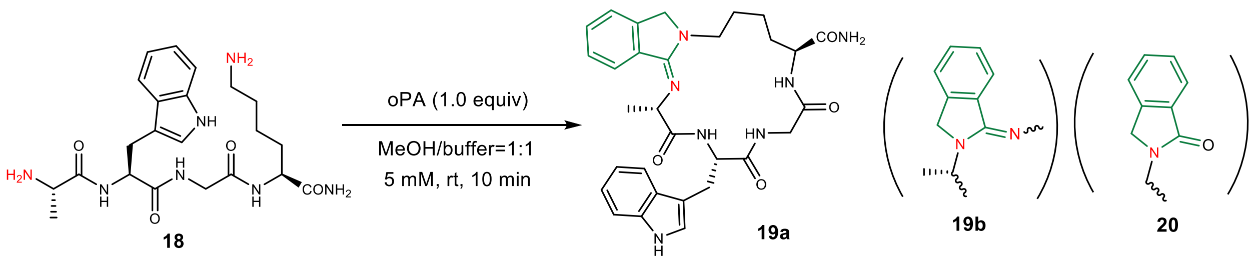

Cyclization of **18** at various pH were monitored via LC-MS. (pH 2.0-3.0→MeOH/H_2O/HCO_2H (50/50/1); pH 5.0-9.0→PBS buffer; pH 10.0→carbonate buffer).

Although the pH values between 8.0 and 10.0 had little influence on the cyclization efficiency, the yield of **19a** started to drop below pH 7.0 along with an increasing amount of byproduct **20**.

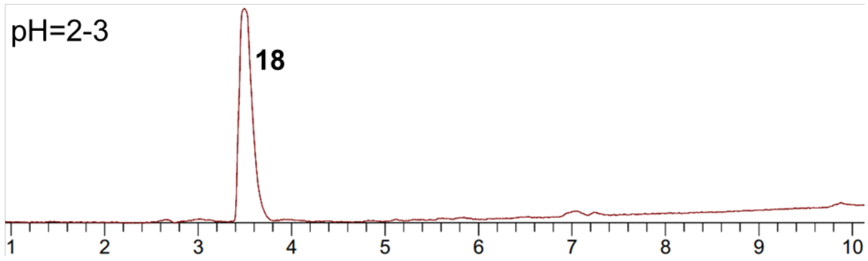

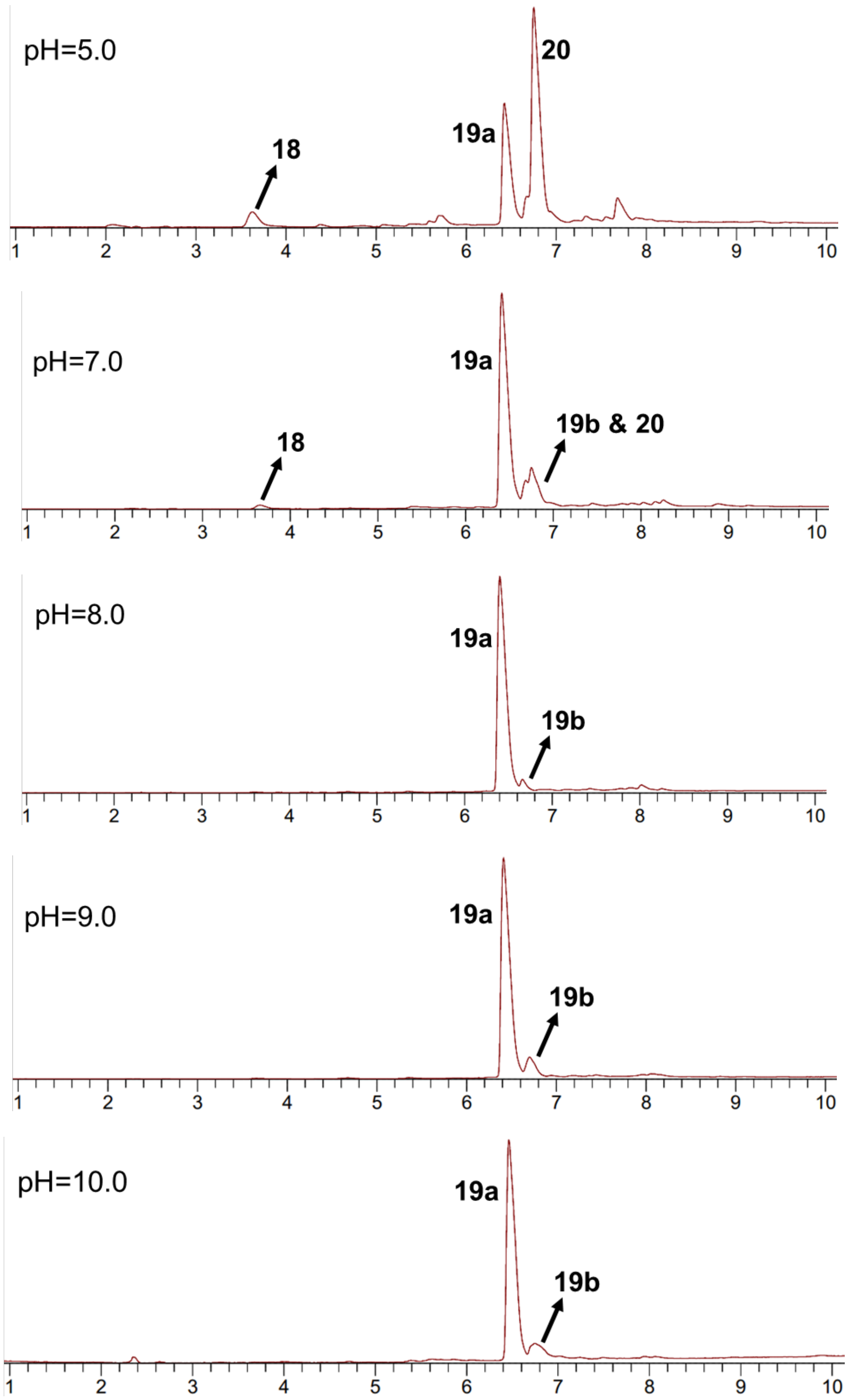

Supplementary Fig. 68. LC traces of compound **18** at different pH.

2.4 Stability test of the cyclic peptides via OPA-2amines reaction

Selected compounds via head-to-side or side-to-side cyclization were treated with PBS (**pH=7.3**), 0.01 M NaOH (**pH=12.0**) and 0.1 M HCl (**pH=1.0**) at room temperature for 12 hours to test the stability.

pH=1.0

**Compounds**	**12 h**
**19a**	Stable
**29**	Stable
**44**	Stable
**47**	Stable
**50**	Stable

pH=7.3

**Compounds**	**12 h**
**19a**	Stable
**29**	Stable
**44**	Stable
**47**	Stable
**50**	Stable

pH=12.0

**Compounds**	**12 h**
**19a**	Stable
**29**	Stable
**44**	Stable
**47**	Stable
**50**	Stable

Heating the selected compounds in PBS (**pH=7.3**) at 60 ^{o}C and 100 ^{o}C to test the stability.

60 ^{o}C in PBS (**pH=7.3**)

**Compounds**	**12 h**
**19a**	Stable
**29**	<10%[a]
**44**	Stable
**47**	Stable
**50**	<10%[a]

100 ^{o}C in PBS (**pH=7.3**)

**Compounds**	**2 h[a]**	**12 h[a]**
**19a**	~30%	>90%
**29**	~70%	>90%
**44**	Stable	<10%
**47**	Stable	~50%
**50**	Stable	~30%

a: decomposition

Supplementary Fig. 69. Stability test of the cyclic peptides via OPA-2amines reaction.

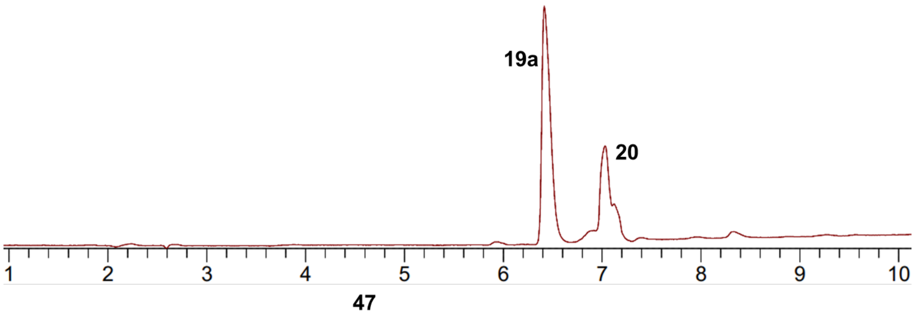

Supplementary Fig. 70. LC trace of the treatment of **19a** in PBS (**pH=7.3**) at 100 ºC for 2 h.

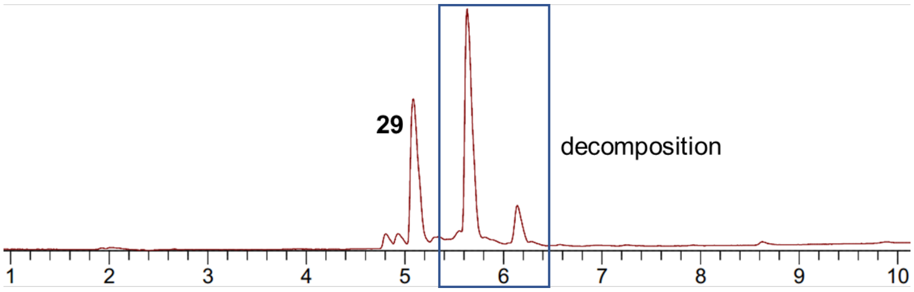

Supplementary Fig. 71. LC trace of the treatment of **29** in PBS (**pH=7.3**) at 100 ºC for 2 h.

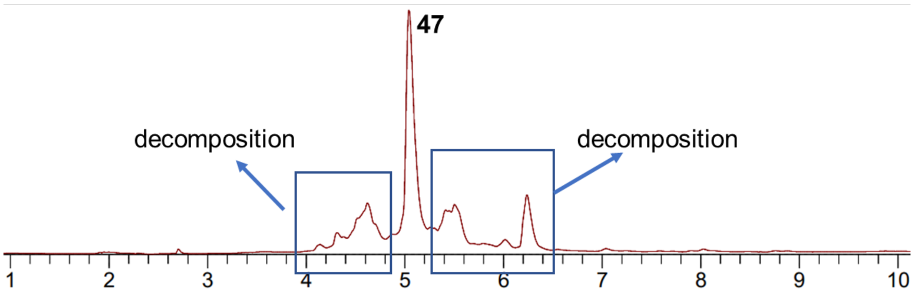

Supplementary Fig. 72. LC trace of the treatment of **47** in PBS (**pH=7.3**) at 100 ºC for 12 h.

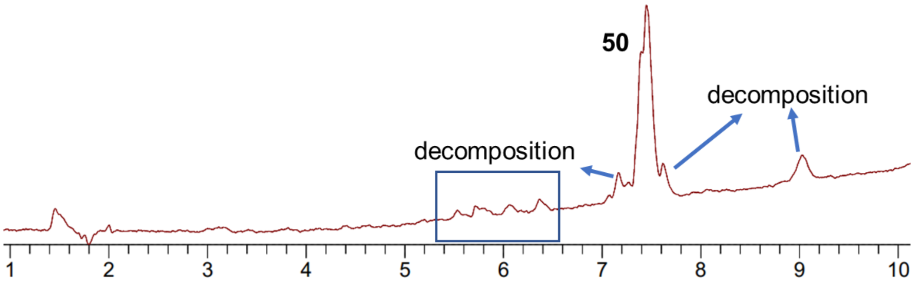

Supplementary Fig. 73. LC trace of the treatment of **50** in PBS (**pH=7.3**) at 100 ºC for 12 h.

3. NMR Spectra

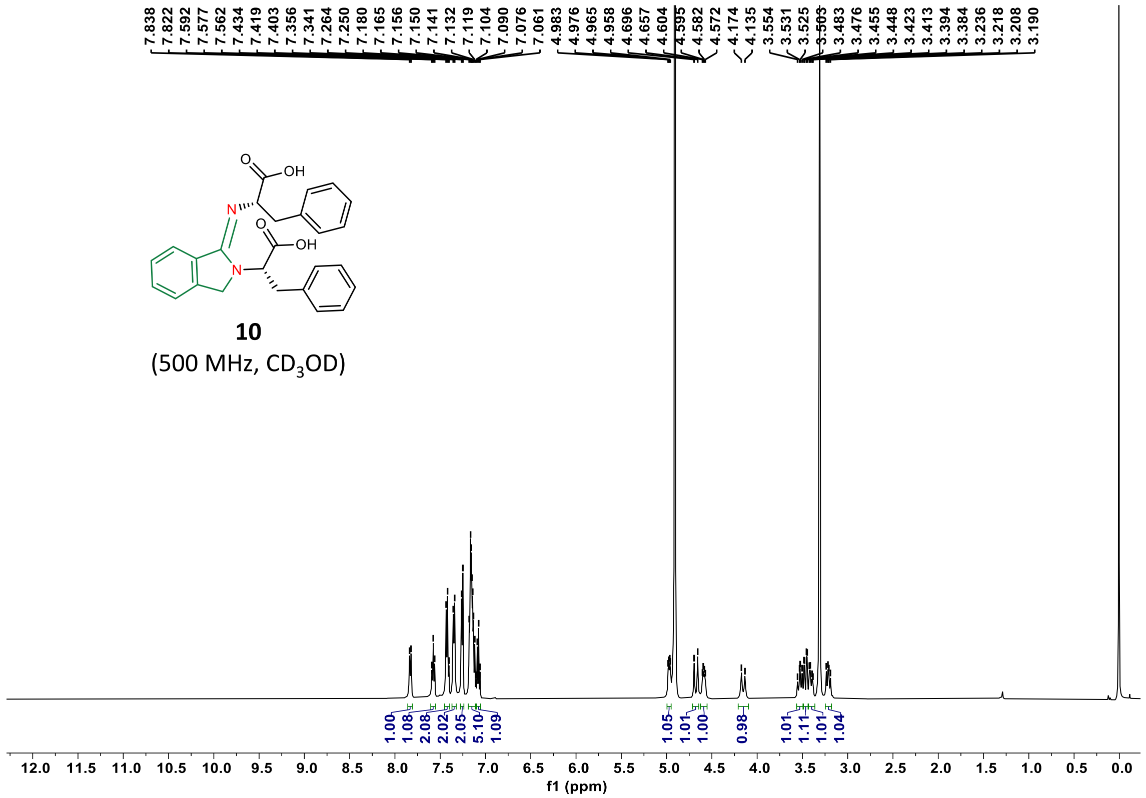

Supplementary Fig. 74. ^{1}H NMR spectrum of compound **10**.

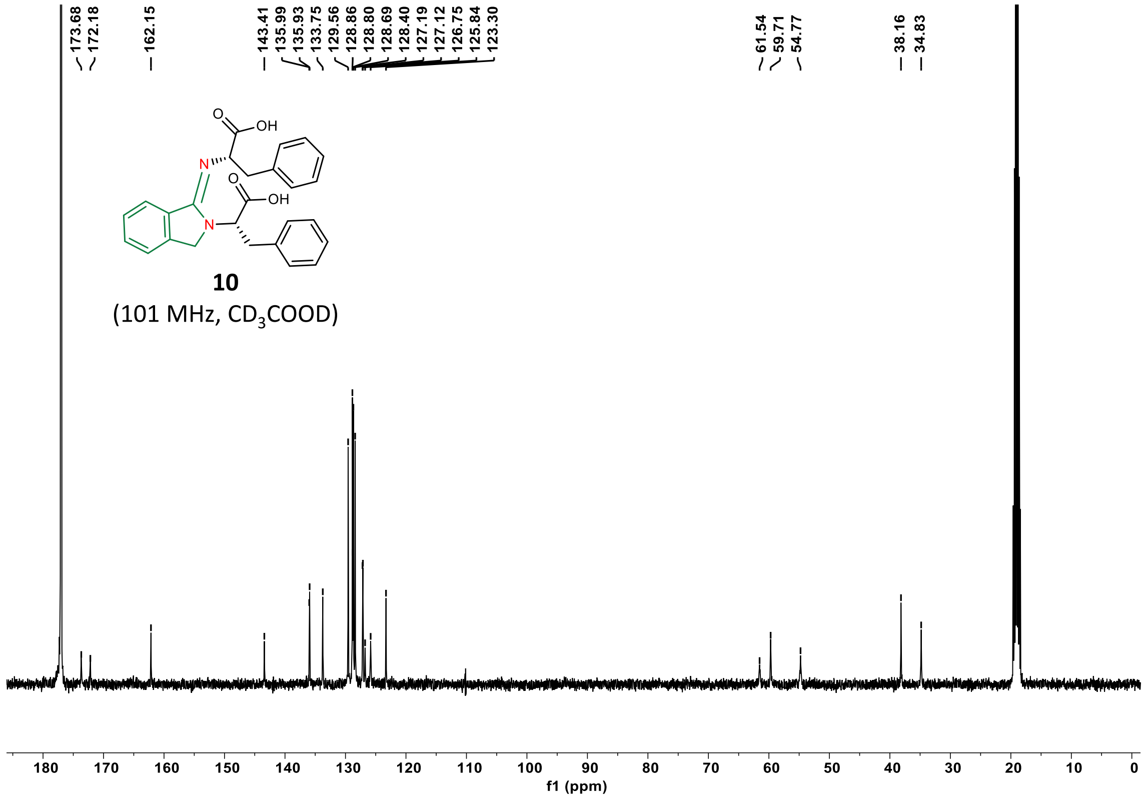

Supplementary Fig. 75. ^{1}C NMR spectrum of compound **10**.

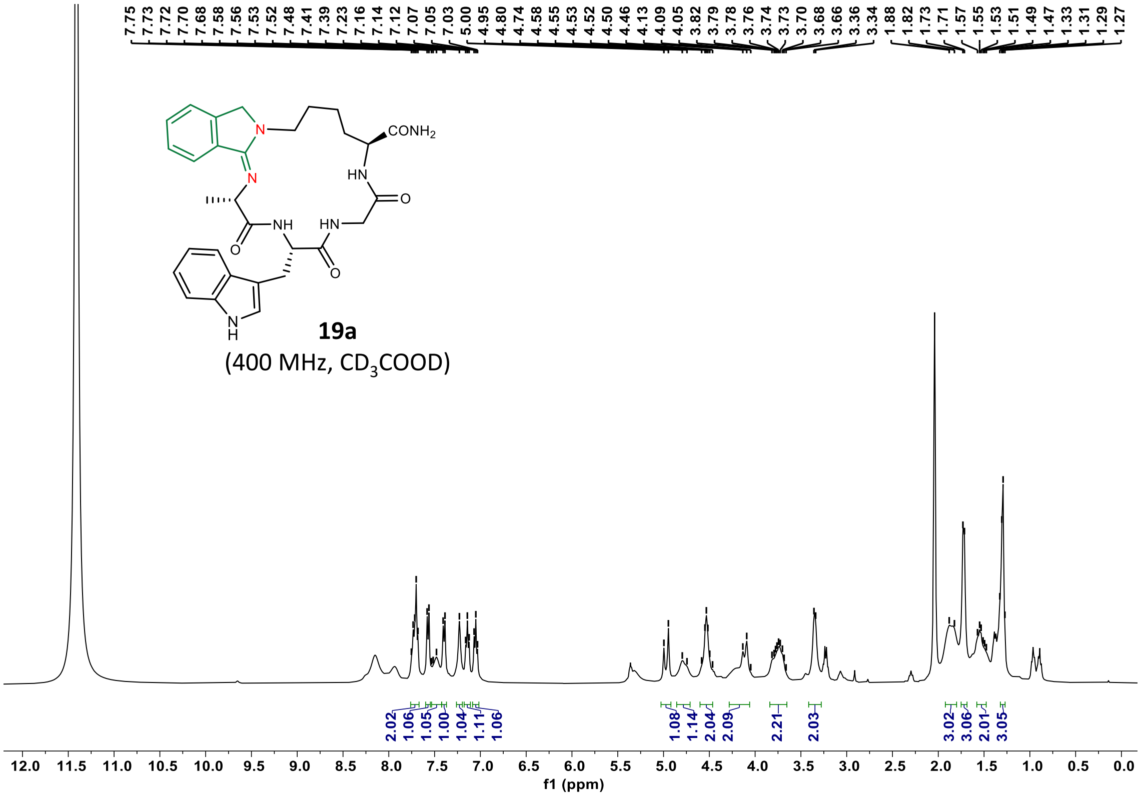

Supplementary Fig. 76. ^{1}H NMR spectrum of compound **19a**.

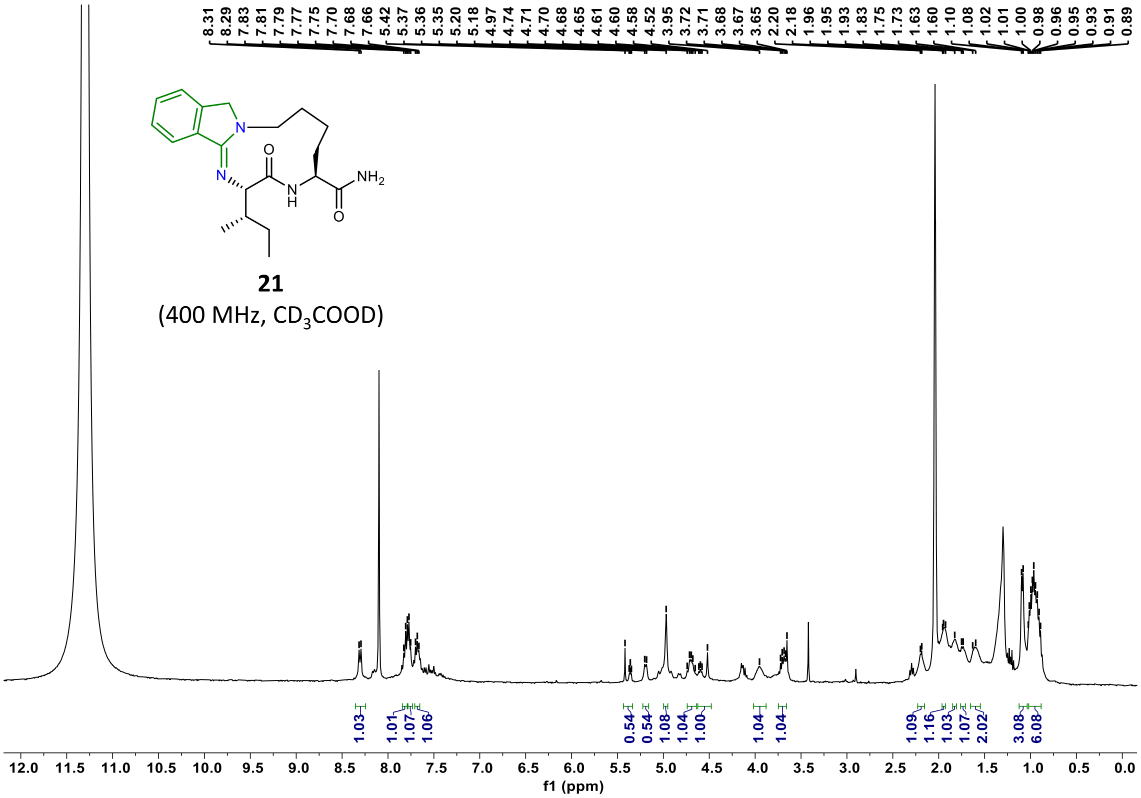

Supplementary Fig. 77. ^{1}H NMR spectrum of compound **21**.

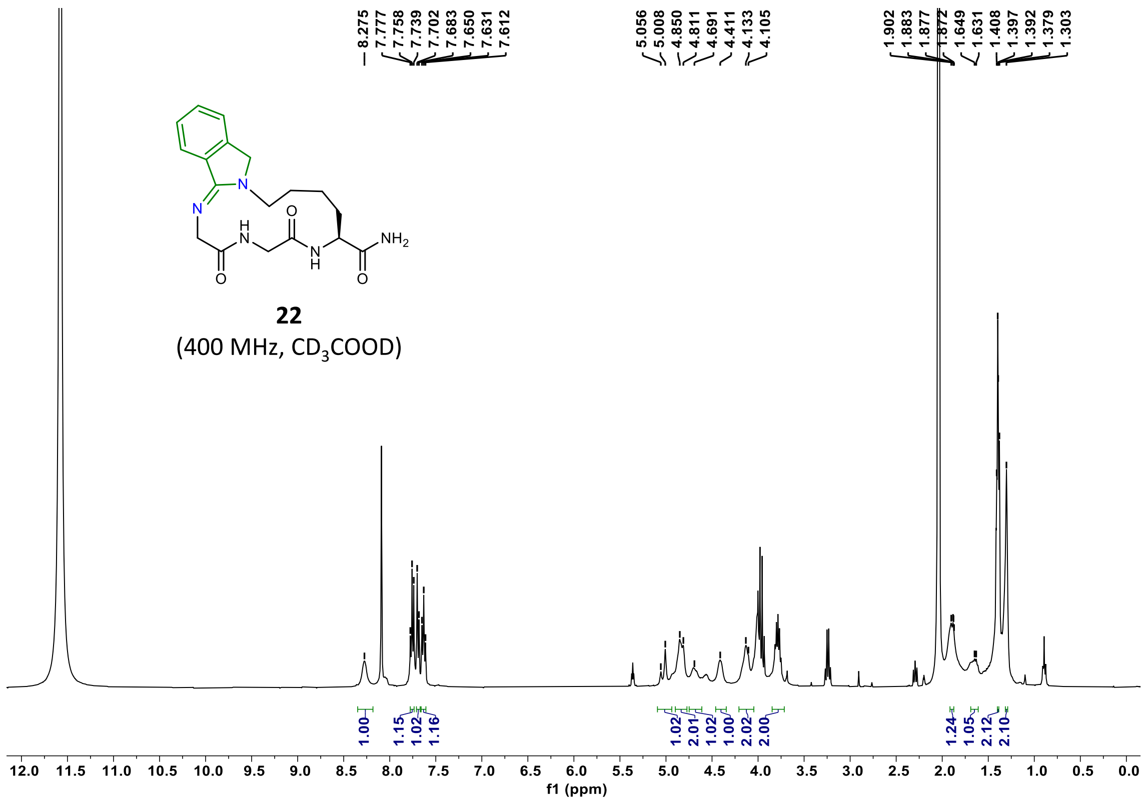

Supplementary Fig. 78. ^{1}H NMR spectrum of compound **22.**

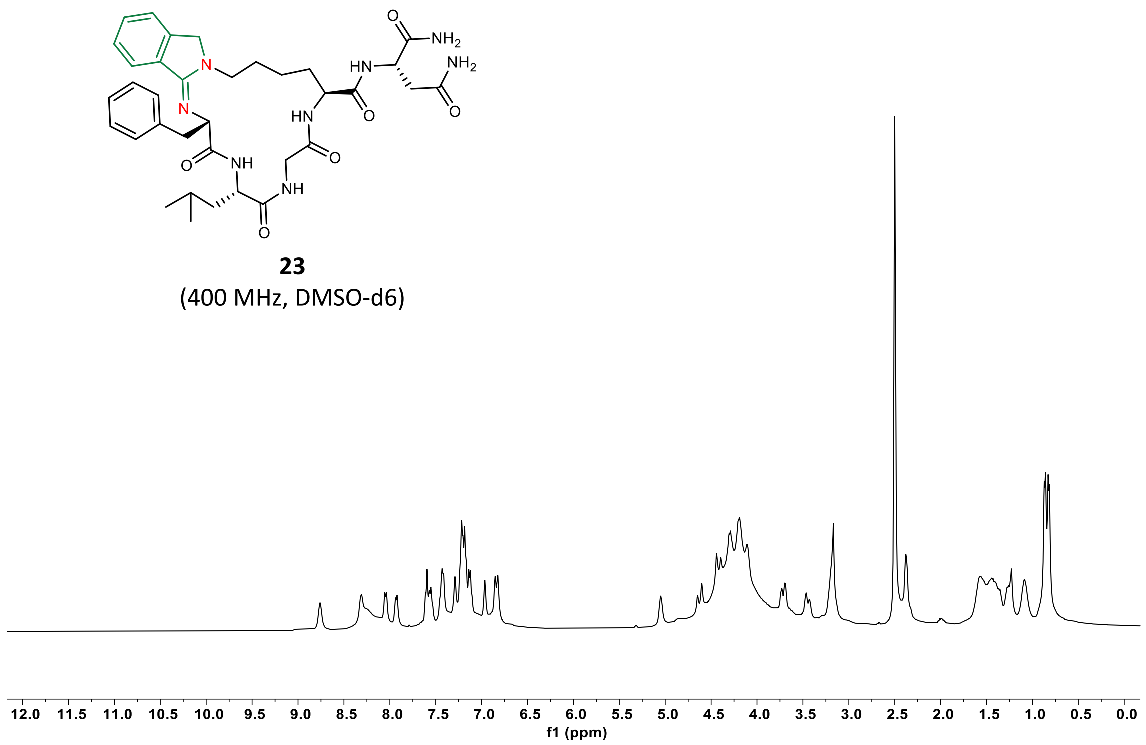

Supplementary Fig. 79. ^{1}H NMR spectrum of compound **23.**

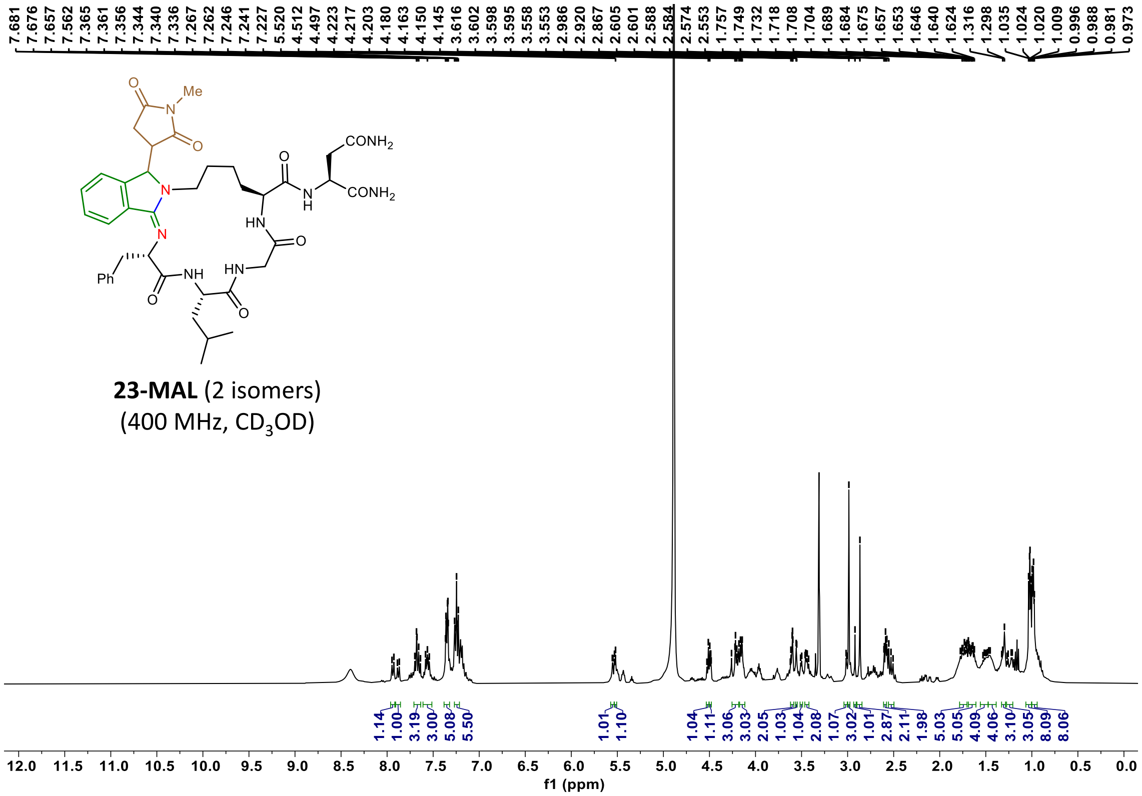

Supplementary Fig. 80. ^{1}H NMR spectrum of compound **23-MAL.**

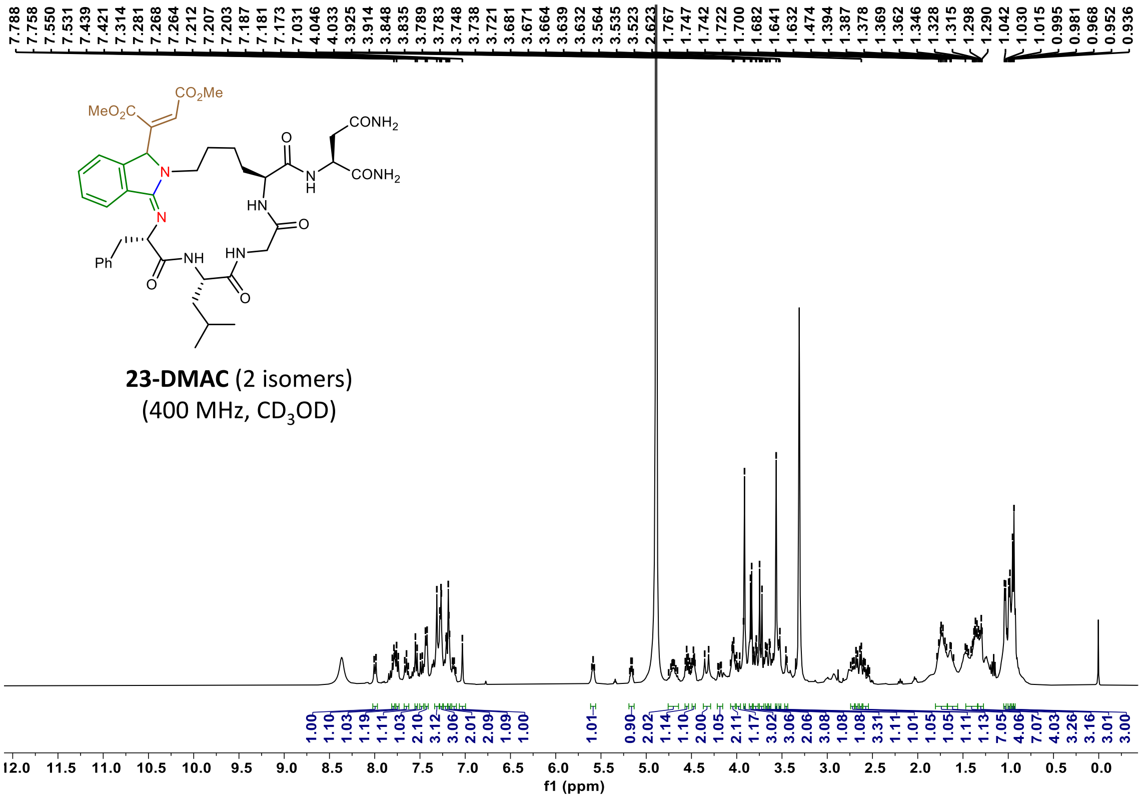

Supplementary Fig. 81. ^{1}H NMR spectrum of compound **23-DMAC.**

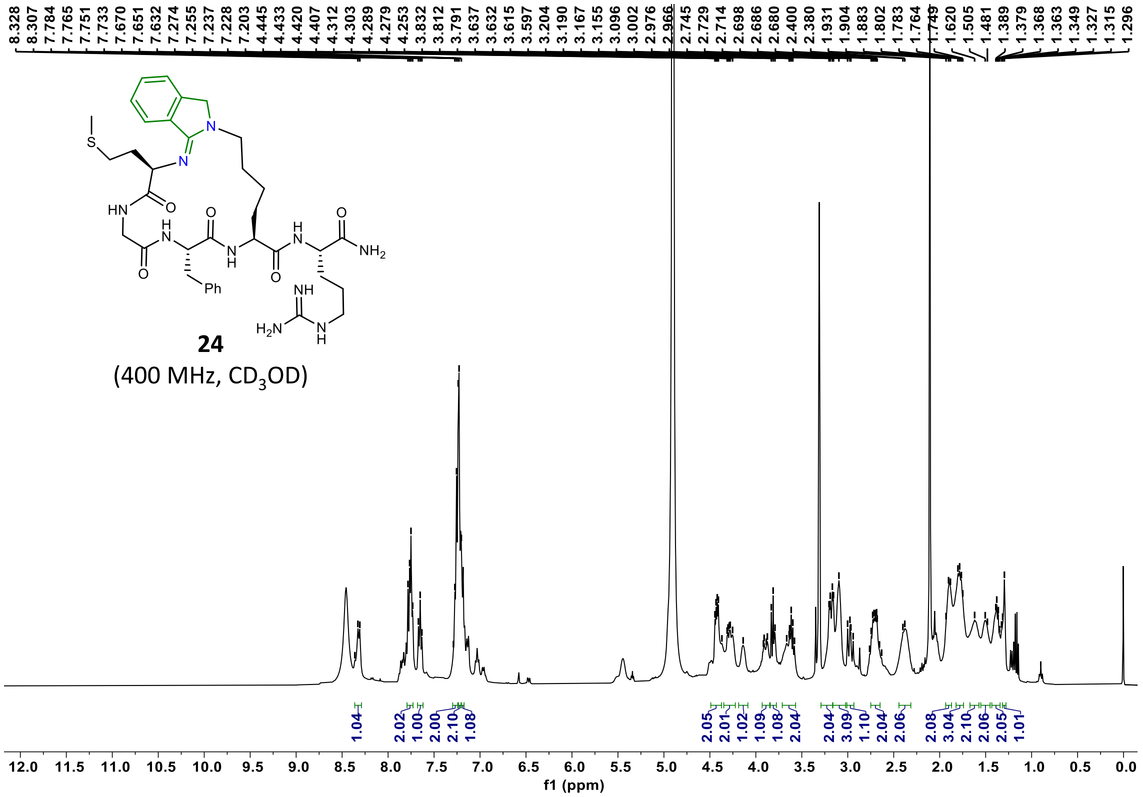

Supplementary Fig. 82. ^{1}H NMR spectrum of compound **24.**

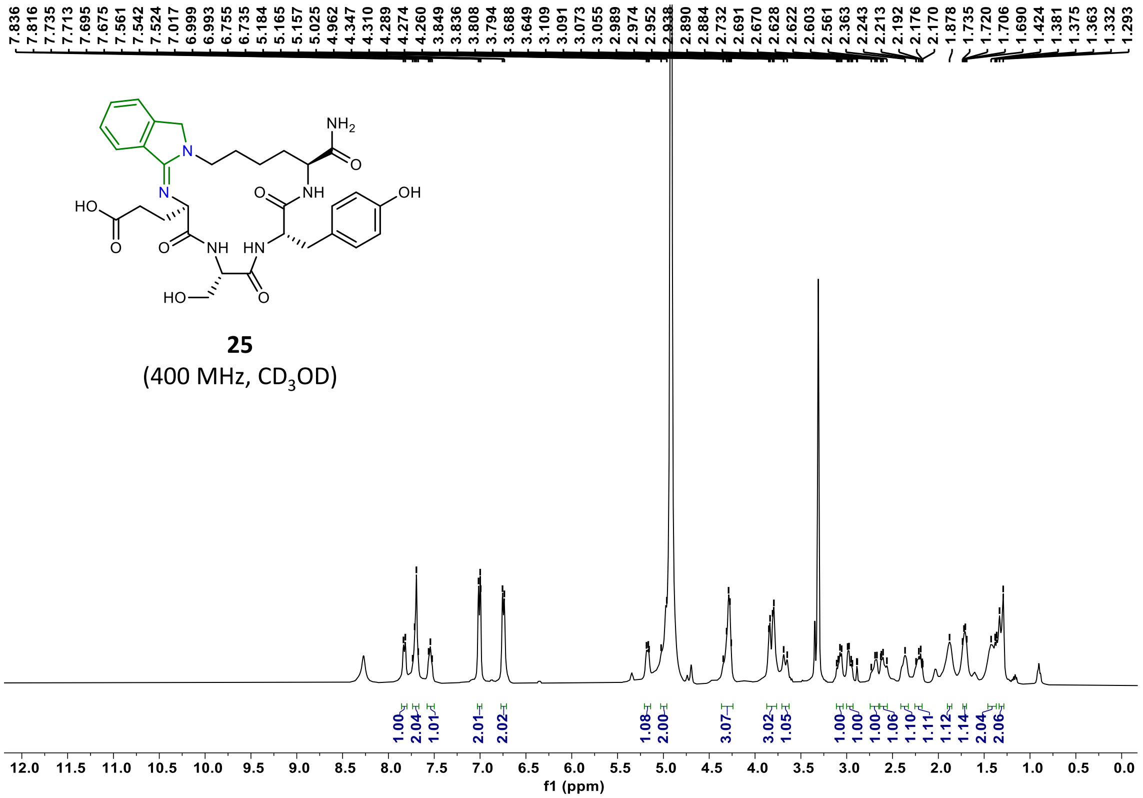

Supplementary Fig. 83. ^{1}H NMR spectrum of compound **25.**

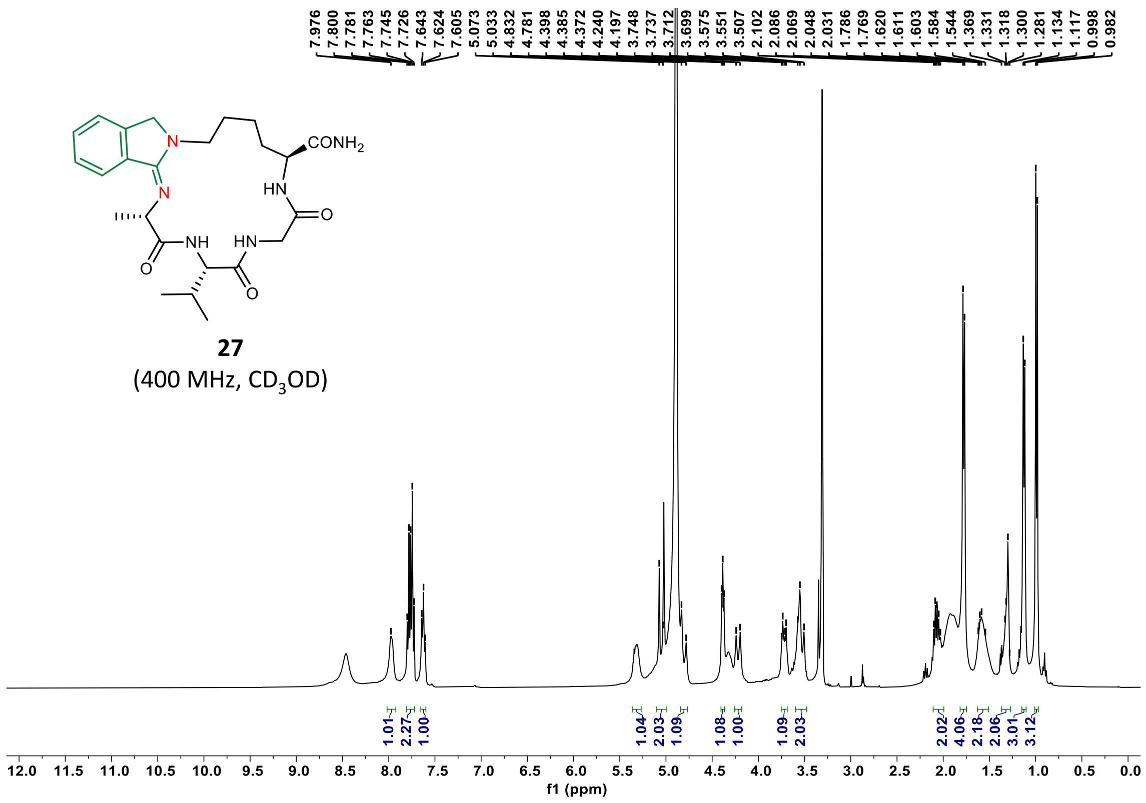

Supplementary Fig. 84. ^{1}H NMR spectrum of compound **27.**

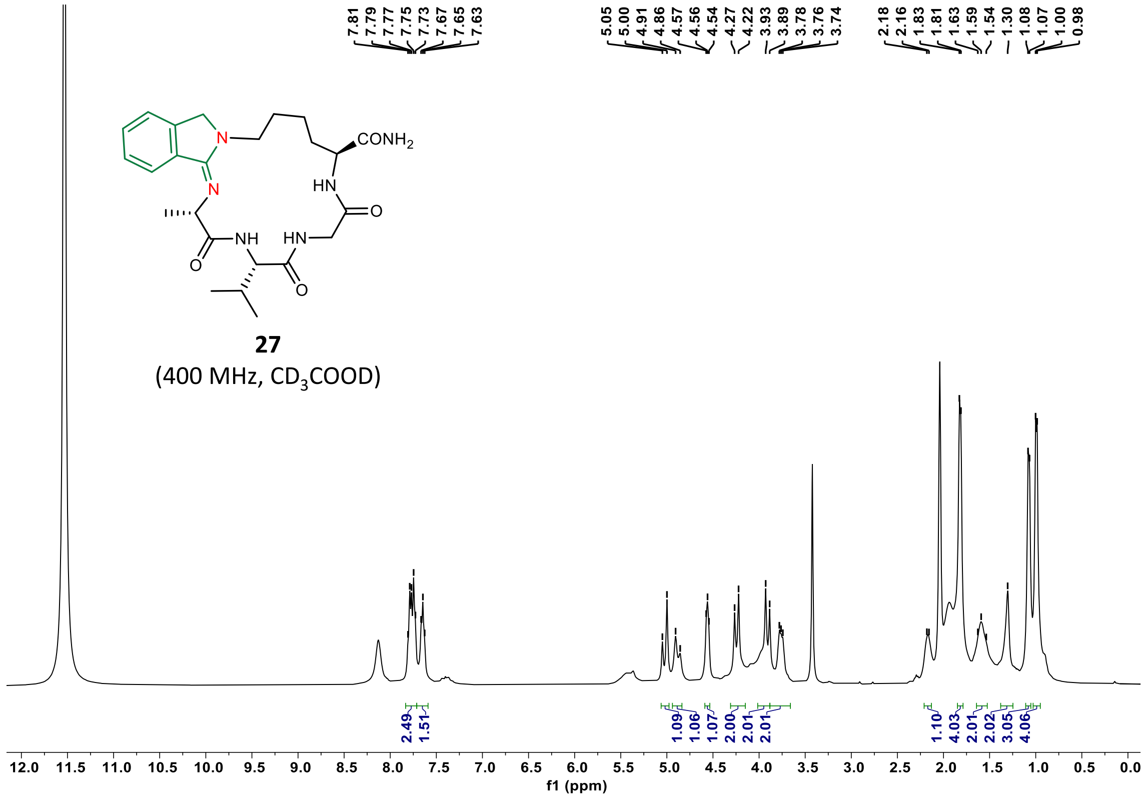

Supplementary Fig. 85. ^{1}H NMR spectrum of compound **27.**

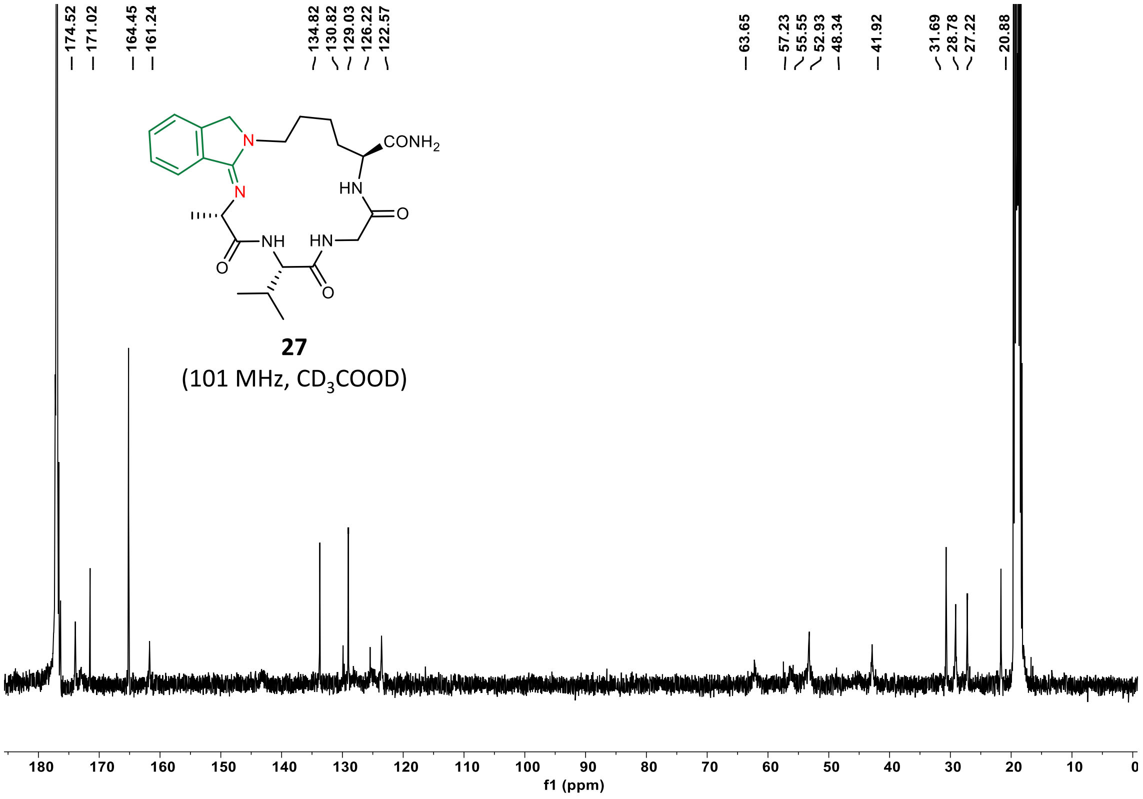

Supplementary Fig. 86. ^{13}C NMR spectrum of compound **27.**

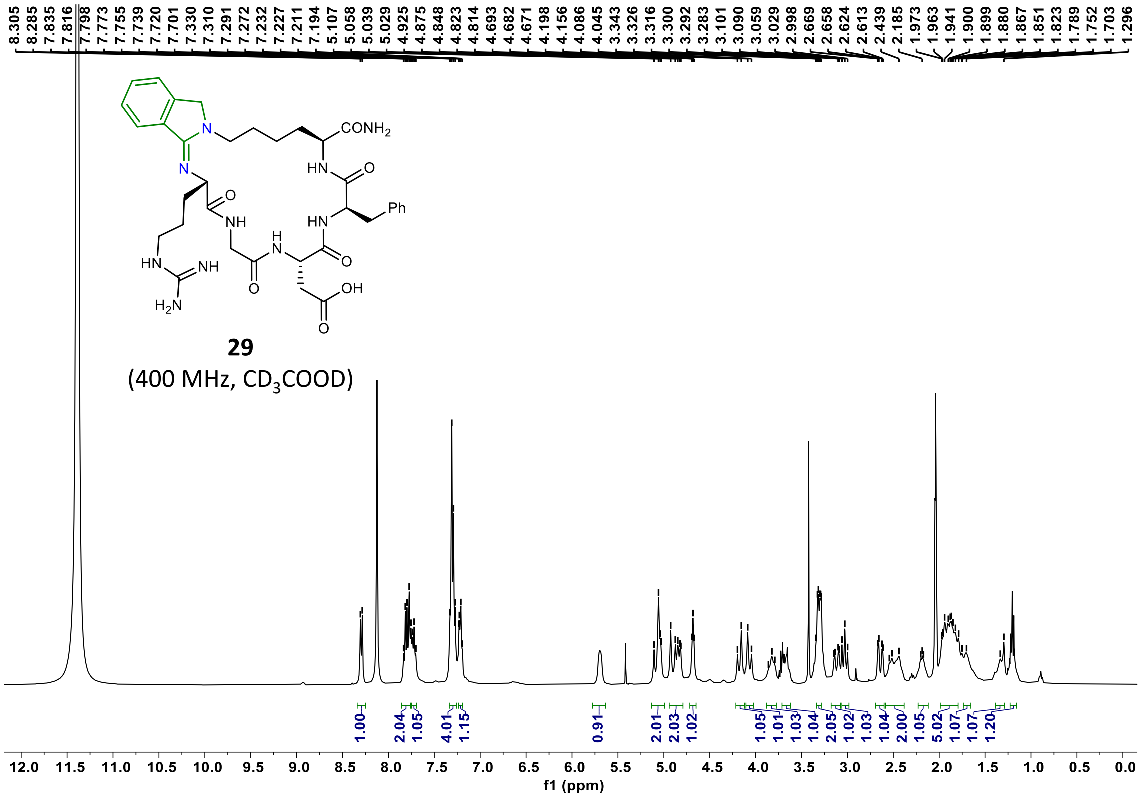

Supplementary Fig. 87. ^{1}H NMR spectrum of compound **29.**

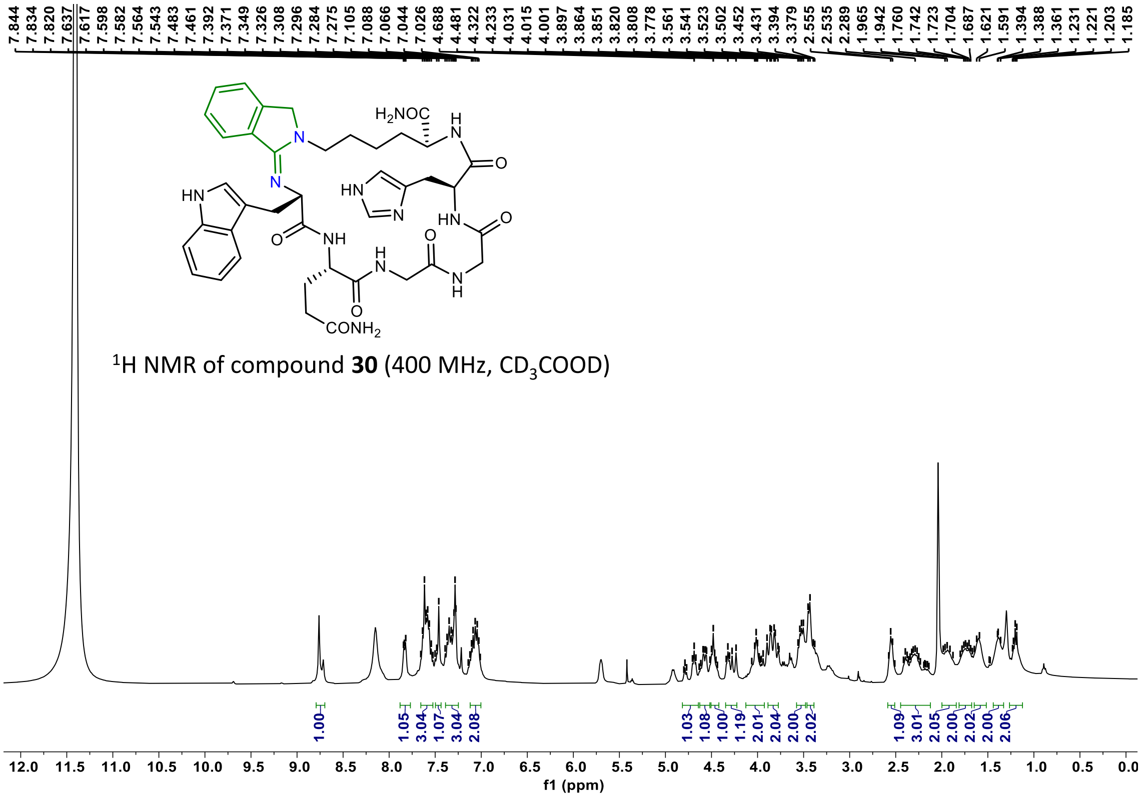

Supplementary Fig. 88. ^{1}H NMR spectrum of compound **30.**

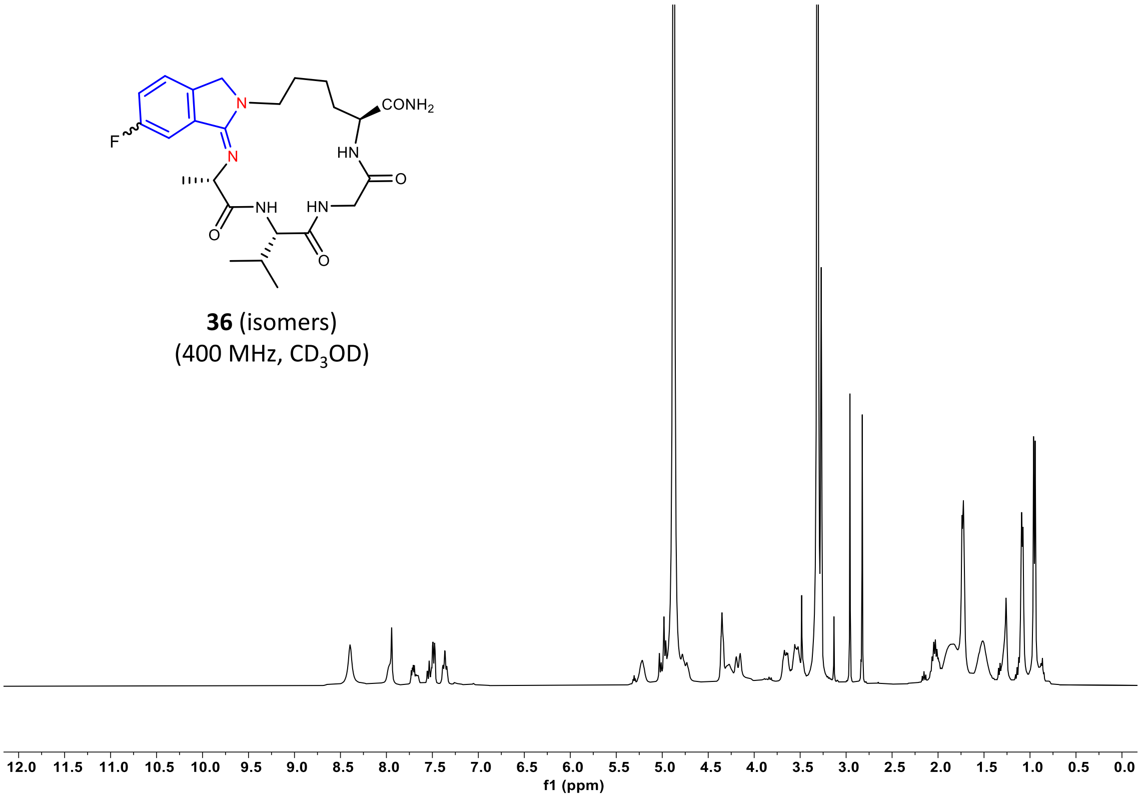

Supplementary Fig. 89. ^{1}H NMR spectrum of compound **36.**

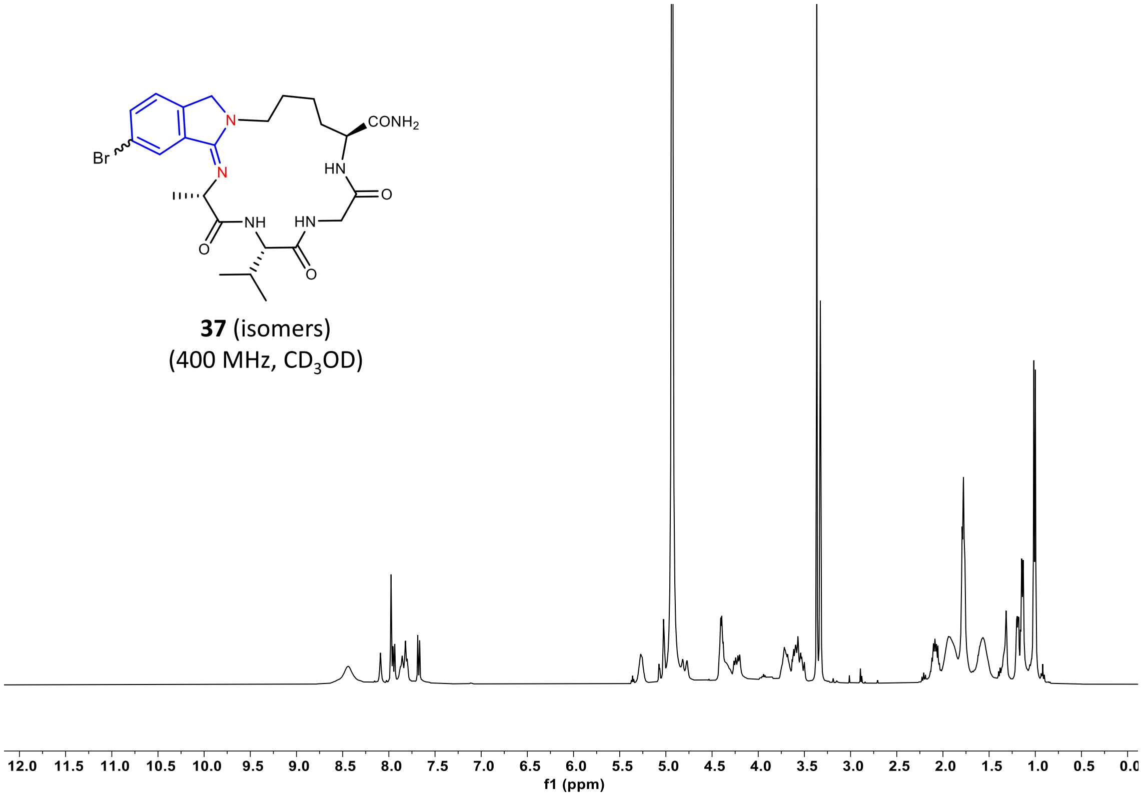

Supplementary Fig. 90. ^{1}H NMR spectrum of compound **37.**

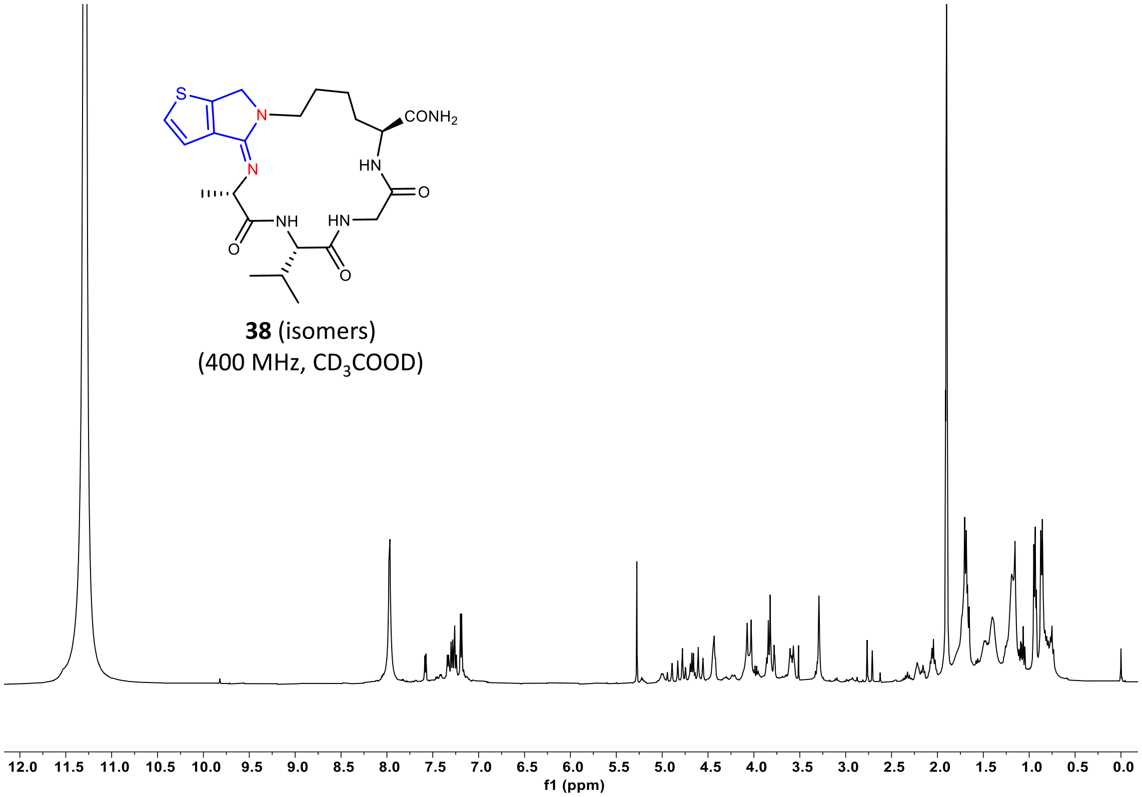

Supplementary Fig. 91. ^{1}H NMR spectrum of compound **38.**

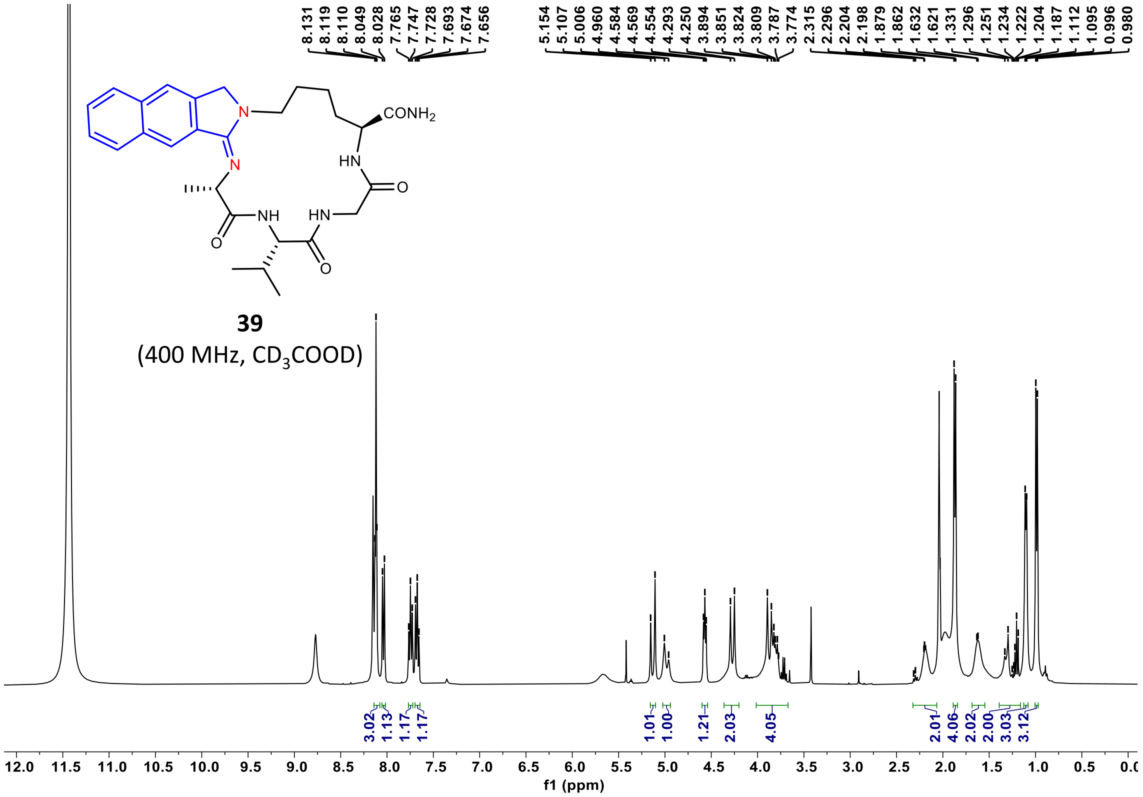

Supplementary Fig. 92. ^{1}H NMR spectrum of compound **39.**

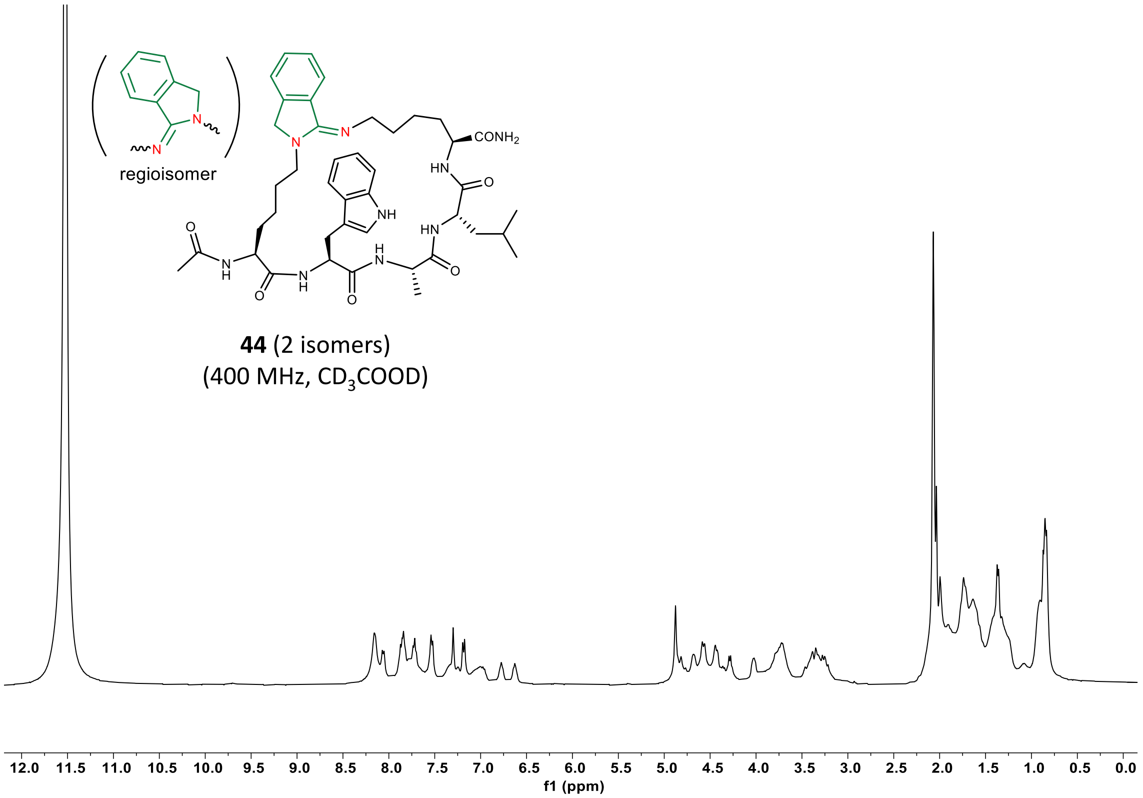

Supplementary Fig. 93. ^{1}H NMR spectrum of compound **44.**

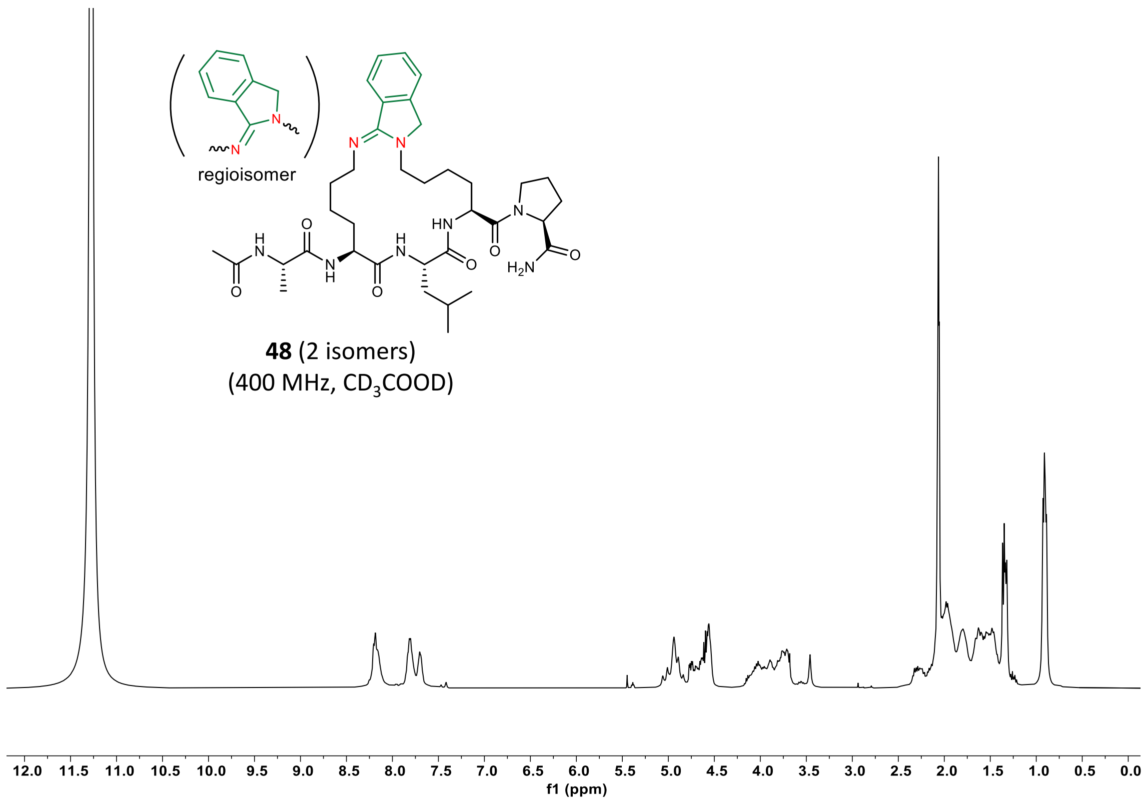

Supplementary Fig. 94. ^{1}H NMR spectrum of compound **48.**

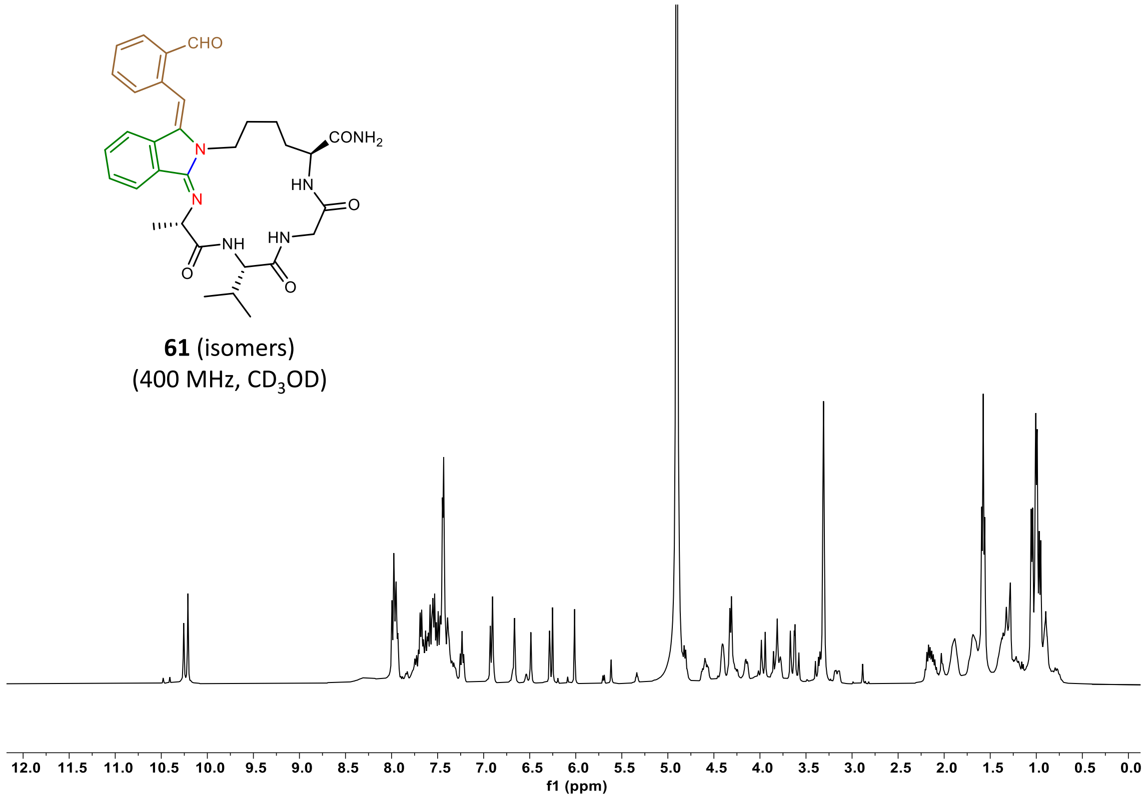

Supplementary Fig. 95. ^{1}H NMR spectrum of compound **61.**

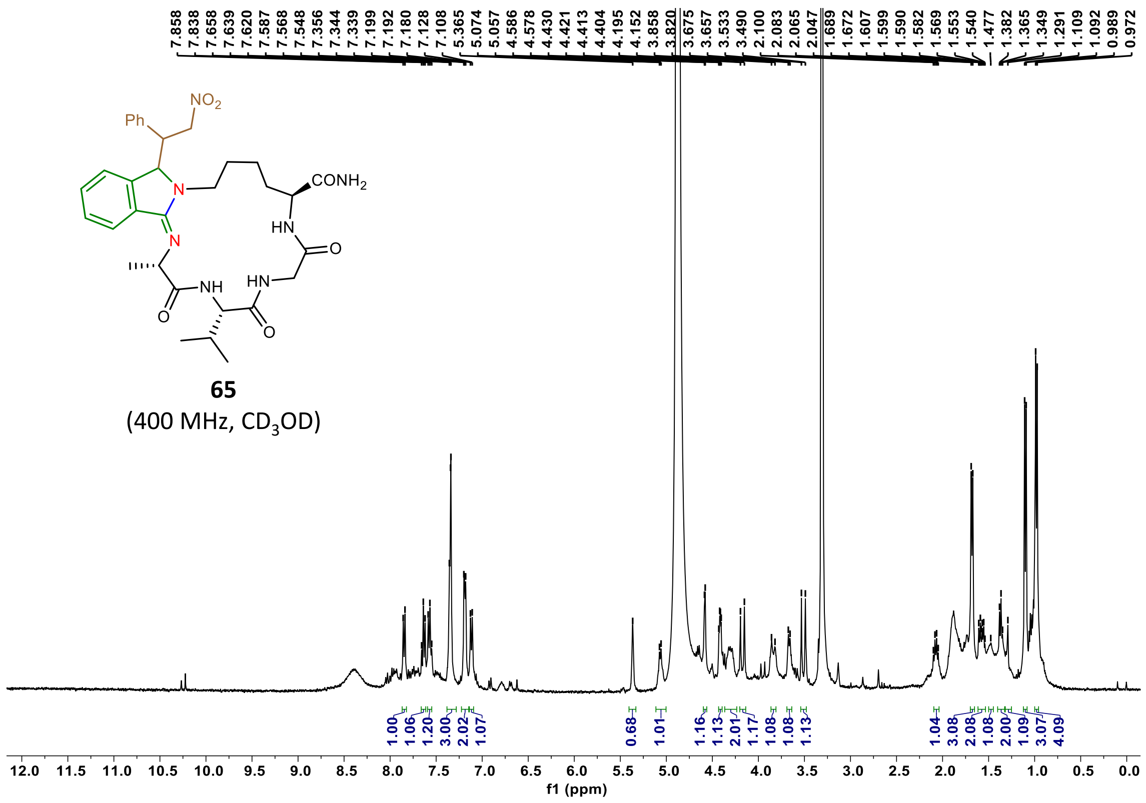

Supplementary Fig. 96. ^{1}H NMR of compound **65.**

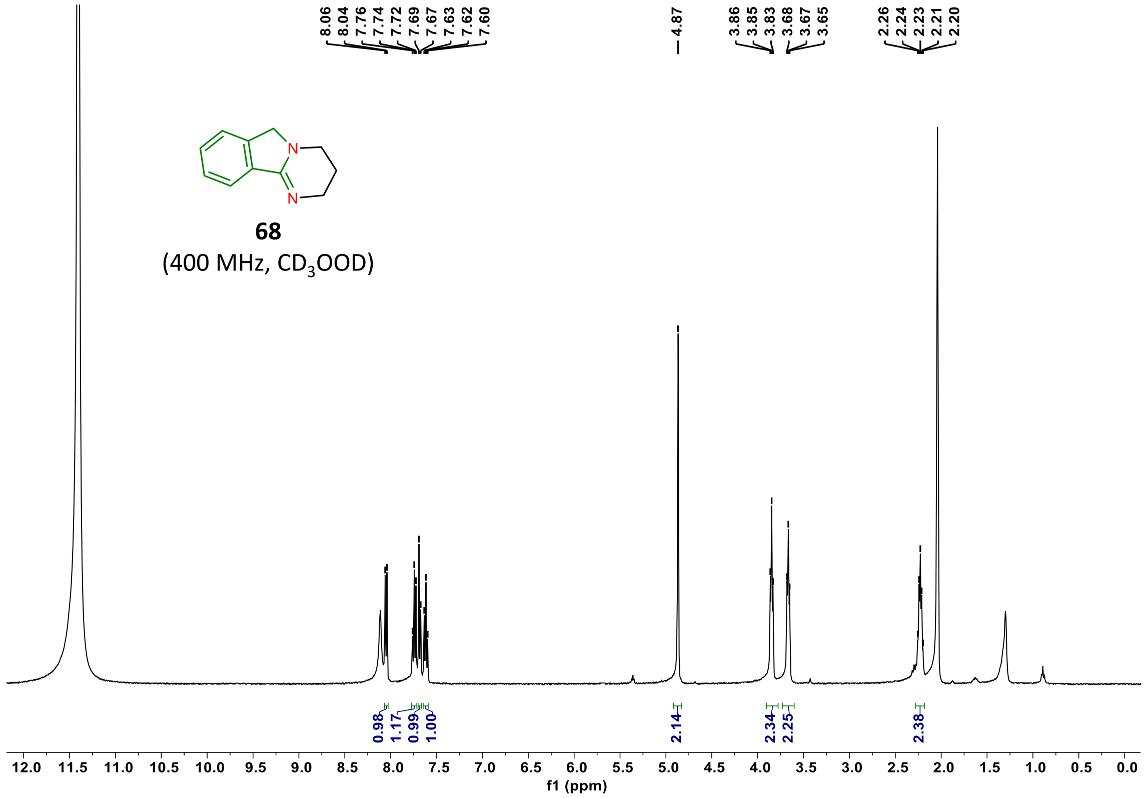

Supplementary Fig. 97. ^{1}H NMR of compound **68.**

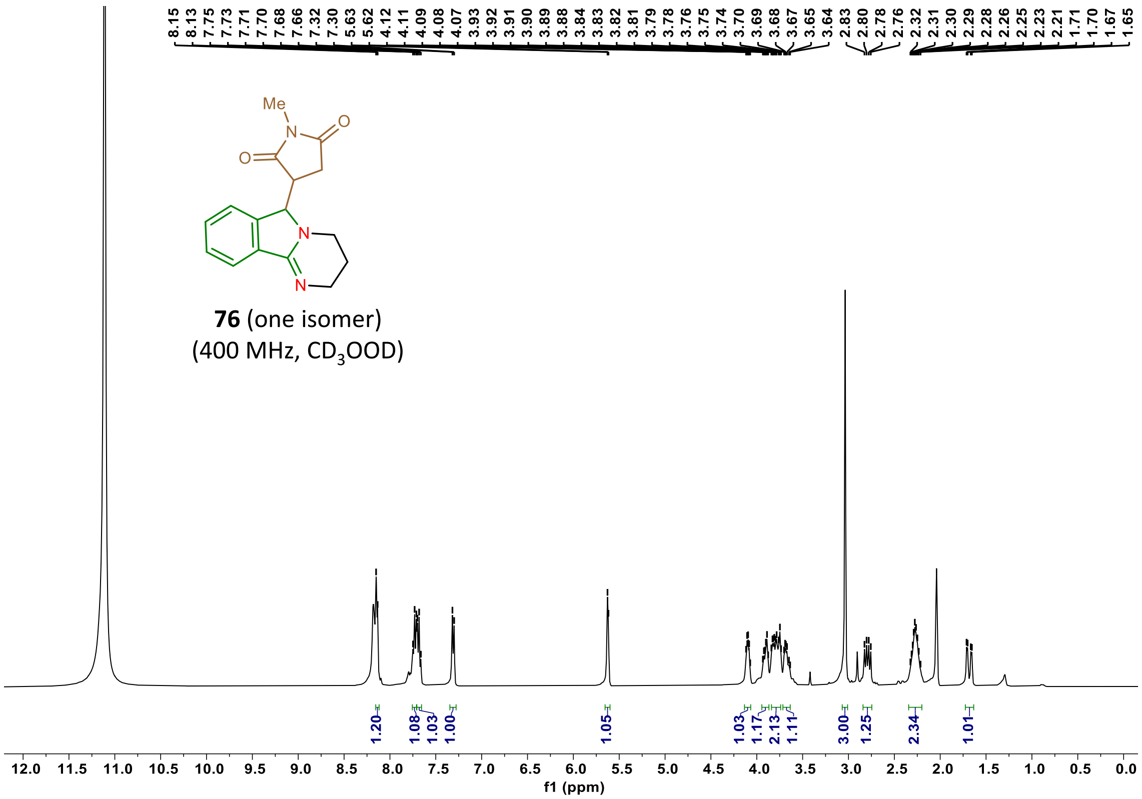

Supplementary Fig. 98. ^{1}H NMR spectrum of compound **76.**

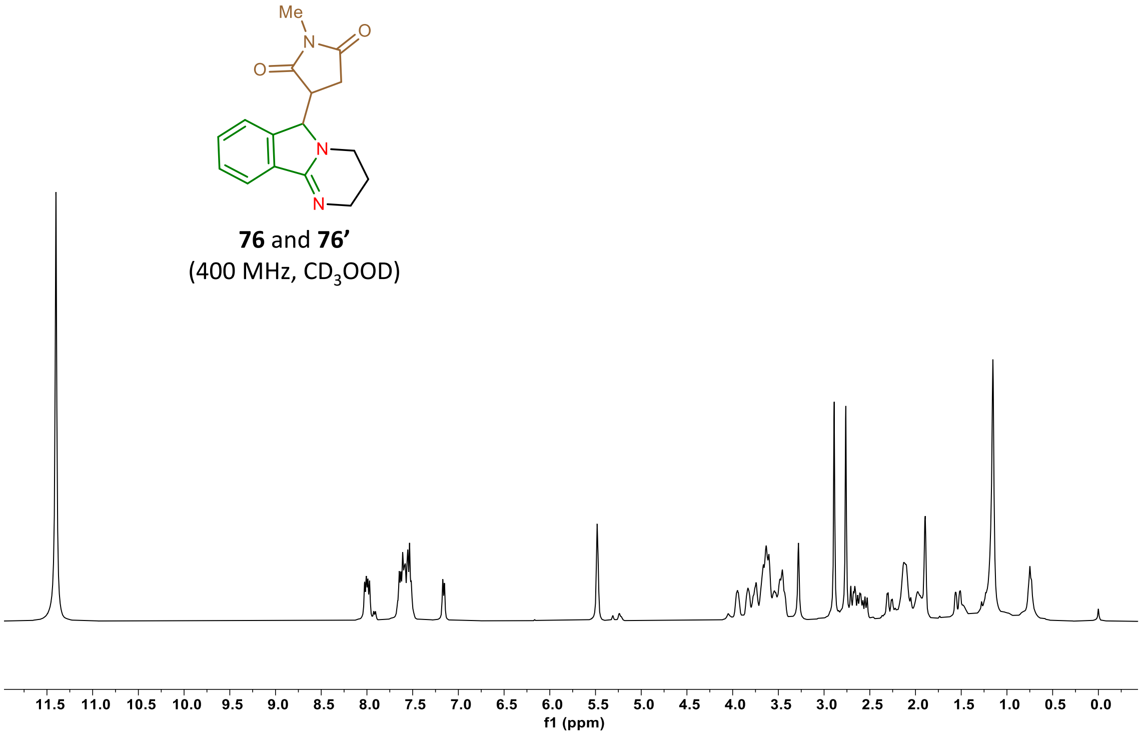

Supplementary Fig. 99. ^{1}H NMR spectrum of compound **76** and **76’.**

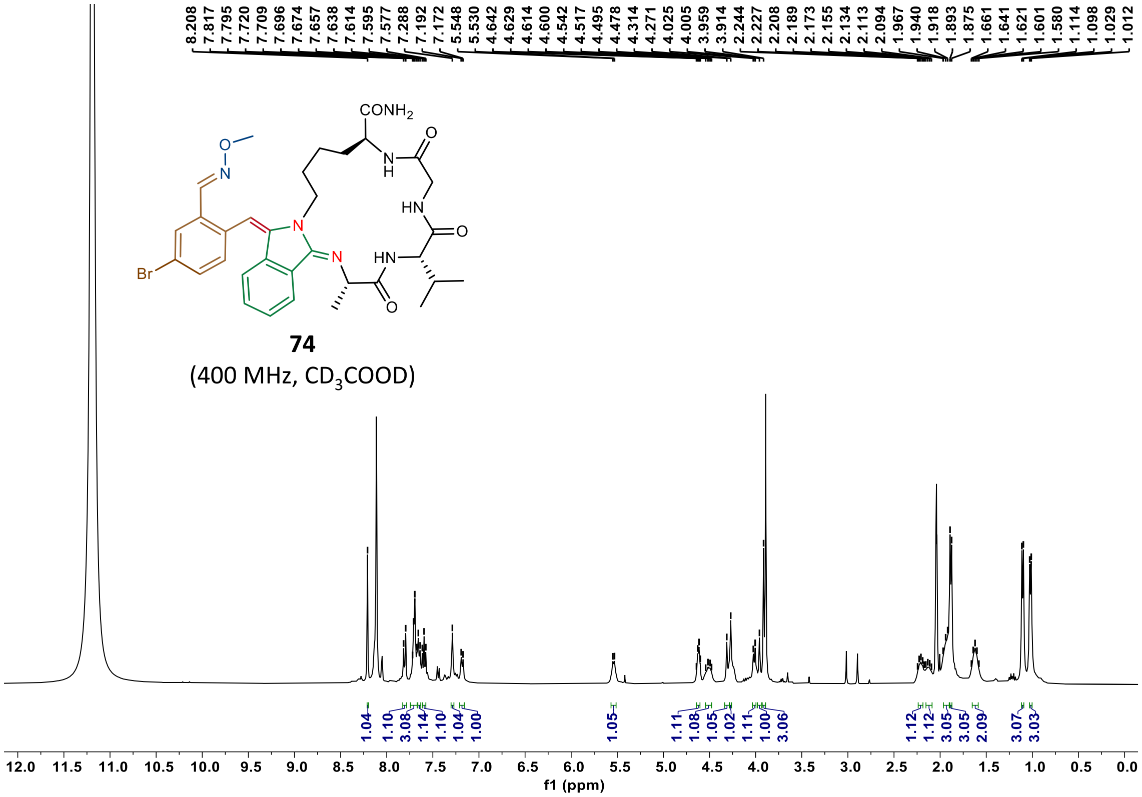

Supplementary Fig. 100. ^{1}H NMR spectrum of compound **74.**

4. Supplementary References

1. Todorovic, M., Schwab, D., Zeisler, J., Zhang, C., F Bénard, Perrin, M. *Angew. Chem. Int. Ed.* **2019**, *131*, 14258.
2. Zhang Y., Zhang Q., Wong C., Li X. *J. Am. Chem. Soc.* **2019**, *141*, 12274.
3. Kutchukian, S., Yang, S., Verdine, G., Shakhnovich, I. *J. Am. Chem. Soc.*, **2009**, *131*, 4622.
4. Kozaki I., Suzuki T., You S., Shimizu K., Honda H. *J. Pep. Sci.* **2021**, *27*, e3287.
5. Chang Y., Gravesb B., Guerlavaisa V., Tovarb C., Packmanb K., Tob K., Olson K., Kesavana K., Gangurdea P., Mukherjee A., Bakera T., Darlaka K., Elkina C., Filipovicb Z., Qureshib F., Cai H., Berryb P., Feyfanta E., Shi X., Horsticka J., Annis D., Manninga A., Fotouhib N., Nasha H., Vassilevb L.,Sawyera T. *Proc. Natl. Acad. Sci. U. S. A.* **2013**, *110*, E3445.
